# Psilocybin-assisted therapy for treatment-resistant major depressive disorder in a public healthcare setting: a randomized controlled trial

**DOI:** 10.1038/s41591-026-04541-0

**Published:** 2026-08-06

**Authors:** James J. Rucker, Tim Mantingh, Jess Kerr-Gaffney, Catherine Bird, Petrina Chu, Nadav Liam Modlin, Kete Campbell-Coker, Sadie Hambleton, Paige Seath, Rebecca Hignett, Diede Fennema, Elliot Hampsey, Dimosthenis Tsapekos, Rebecca J. Thomas, Joseph Cattell, Hassan Jafari, Camilla Day, Anya Borissova, Miranda Lloyd, Theo Boardman-Pretty, Mathieu Seynaeve, Famia Askari, Michael Creed, Luke Baxter, Raphael Rifkin-Zybutz, Matt Butler, Luke A. Jelen, Ben Carter, Allan H. Young

**Affiliations:** 1 Department of Psychological Medicine, Institute of Psychiatry, Psychology & Neuroscience, King’s College London, London, UK; 2 South London and Maudsley NHS Foundation Trust, London, UK; 3 Department of Biostatistics & Health Informatics, Institute of Psychiatry, Psychology & Neuroscience, King’s College London, London, UK; 4 King’s Clinical Trials Unit, Institute of Psychiatry, Psychology & Neuroscience, King’s College London, London, UK; 5 Division of Psychiatry, Imperial College London, London, UK

**Keywords:** Drug therapy, Depression

## Abstract

Psilocybin-assisted therapy may be a promising new treatment for treatment-resistant depression. We examined the feasibility of administering a single 25-mg dose of psilocybin or placebo with psychological support in a randomized controlled trial design with 6 weeks of follow-up. A two-arm, double-blind, randomized, placebo-controlled feasibility trial was conducted at one National Health Service (NHS) site in England. Eligible participants met *Diagnostic and Statistical Manual of Mental Disorders*, Fifth Edition (DSM-5) criteria for major depressive disorder and had an inadequate response to ≥2 antidepressant treatments or ≥1 antidepressant plus ≥1 psychotherapy. Participants received 25-mg psilocybin or placebo with preparation, dosing support and integration. Primary outcomes were recruitment, retention and estimation of the Montgomery−Åsberg Depression Rating Scale (MADRS) variance. A multilevel regression analysis with an intention-to-treat population was used. Sixty participants were randomized (1:1), balanced by age, sex and prior psilocybin exposure, and 59 of 60 participants completed the MADRS at all follow-up visits. The adjusted between-group difference at week 3 on the MADRS was −10.41 (95% confidence interval: −14.86 to −5.95; Cohen’s *d* = −1.70), favoring psilocybin, which was sustained at week 6. In total, 123 and 164 nonserious adverse events occurred in the placebo and psilocybin arms, respectively. Findings support a future confirmatory trial. EudraCT no.: 2018-003573-97.

## Abstract (web summary)

In a publicly funded trial undertaken in the NHS in England, 60 adults with treatment-resistant major depression received psilocybin or placebo alongside standardized psychological support, with psilocybin showing significant improvements in depression symptoms, good tolerability and support for further research in public healthcare settings.

## Main

Major depressive disorder (MDD) is a disabling and economically costly mental disorder, affecting approximately 300 million people worldwide^1^. Treatment-resistant depression (TRD), commonly defined as failure to respond to at least two antidepressants at therapeutic dose and duration, affects one-third of those who suffer from MDD and is associated with a poorer prognosis and higher economic costs^2^. Treatment options with best evidence for efficacy in TRD are ketamine, esketamine, adjunctive psychotherapy, electroconvulsive therapy and repetitive transcranial magnetic stimulation^2^. However, access to these specialized treatments is limited.

Before they were classified under Schedule I of the 1971 UN Conventions on Narcotic Substances, classic psychedelic drugs such as psilocybin were marketed as treatments in psychiatry, with some studies suggesting improvements in those with depression and alcoholism^3^. The active metabolite of psilocybin, psilocin—a partial agonist of the 5-HT_2A_ receptor—accounts for many of the characteristic subjective effects, including heightened emotions, mystical experiences, altered perceptions and ego dissolution, although activation of other serotonin receptors is also contributory^4^. These subjective effects may facilitate the putative therapeutic effects of psilocybin^5^.

Modern clinical research into psilocybin began again in the 2000s, with small open-label trials reporting symptom improvements and indications of safety in conditions such as obsessive-compulsive disorder (OCD)^6^ and TRD^7^. Our group previously conducted a phase 1 randomized, placebo-controlled safety study in which 89 healthy volunteers were randomized 1:1:1 to receive a single dose of placebo, 10 mg of psilocybin or 25 mg of psilocybin, with 3 months of follow-up^8^. No serious adverse events (SAEs) or adverse events (AEs) that led to withdrawal were reported, and data from cognitive tasks indicated no negative effects of psilocybin when compared to placebo.

Prior to the funding of this study, a single open-label, publicly funded pilot study had been completed in 20 patients with TRD in England^7^. The present trial built upon this pilot, exploring the feasibility of a randomized controlled trial (RCT) design that included a placebo. While this trial was being conducted, additional one-arm open-label trials in TRD^9,10^, phase 2 RCTs in non-treatment-resistant MDD^11–14^ and one multicenter phase 2b trial in TRD^15^ were completed; however, this trial is, to our knowledge, the first to specifically assess feasibility within an NHS setting in England using a true placebo control.

## Results

### Patient disposition

**Fig. 1:**
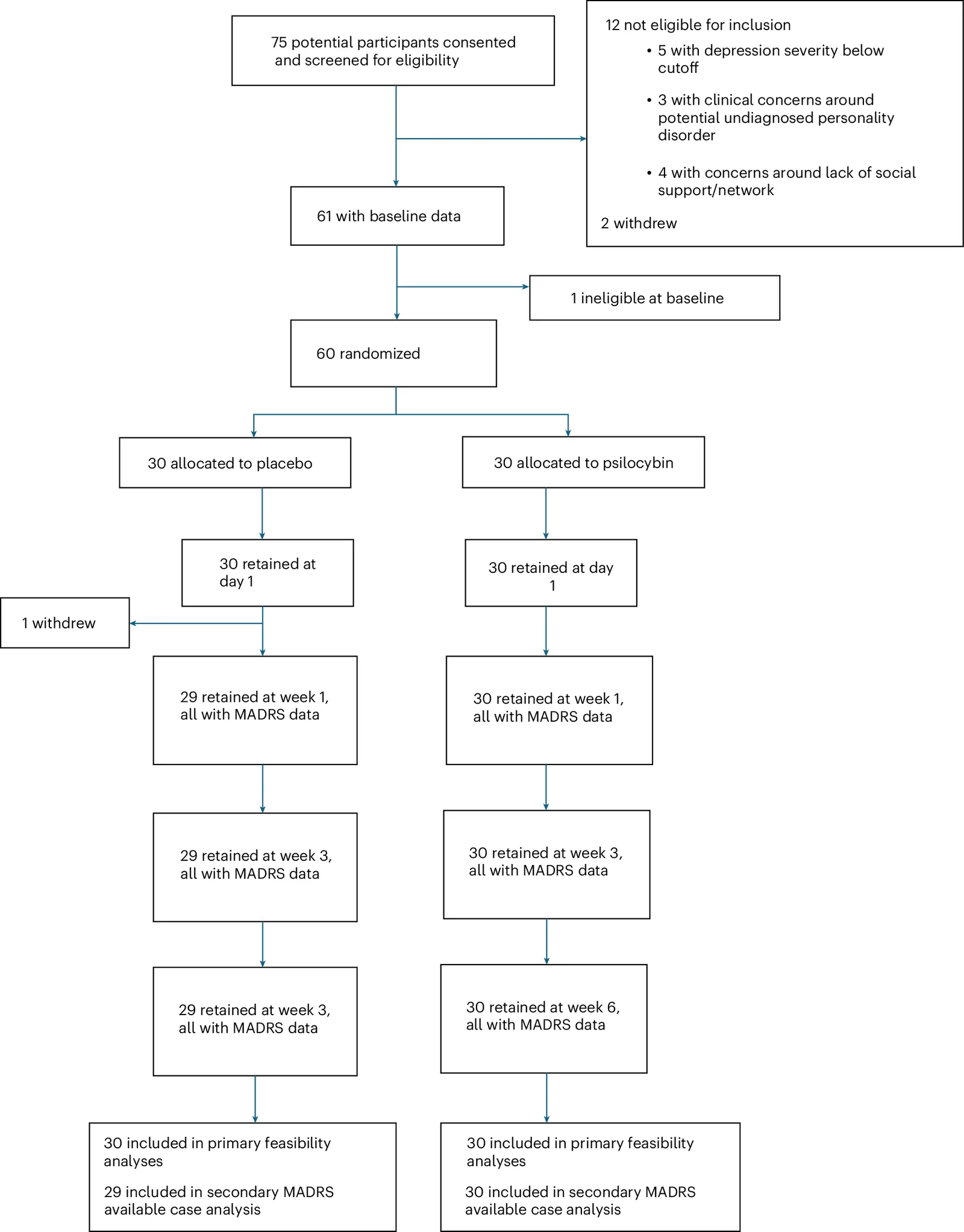
CONSORT diagram. PsiDeR, Psilocybin in Depression Resistant to Standard Treatments.

Between 21 June 2021 and 3 July 2024, 75 potential participants consented and were screened for eligibility. Sixty participants were randomized to either psilocybin or placebo arms; 30 participants were allocated to each arm (Fig. 1). All 60 participants were included in primary feasibility analyses. One participant withdrew in the placebo arm before contributing follow-up MADRS data and was, thus, excluded from mixed models. Participant demographics are summarized in Table 1. Demographics were balanced between arms. Median age at randomization was 40.8 years (interquartile range (IQR): 35.2−52.3), and half (30 out of 60, 50%) were female. Median Maudsley Staging Method (MSM) scores at screening indicated that most participants scored within the moderate range for treatment resistance, with a chronic course of illness.

**Table 1:** Minimization and baseline demographics by arm and overall

	Placebo (*N* = 30)	Psilocybin (*N* = 30)	Overall (*N* = 60)
Minimization factor			
Sex, *n* (%)			
Male	15 (50%)	15 (50%)	30 (50%)
Female	15 (50%)	15 (50%)	30 (50%)
Age group, *n* (%)			
25−59	27 (90%)	27 (90%)	54 (90%)
60+	3 (10%)	3 (10%)	6 (10%)
Past psilocybin use, *n* (%)			
No	25 (83%)	26 (87%)	51 (85%)
Yes	5 (17%)	4 (13%)	9 (15%)
Baseline characteristics			
Age at randomization, median (IQR)	40.2 (33.7−52.0)	41.7 (38.6−57.0)	40.8 (35.2−52.3)
HAM-D-17 score, median (IQR)	17.0 (15.0−21.0)	18.5 (16.0−21.0)	18.0 (15.0−21.0)
Ethnicity, *n* (%)			
White	24 (80%)	21 (70%)	45 (75%)
Mixed or multiple ethnic groups	2 (7%)	4 (13%)	6 (10%)
Asian or Asian British	3 (10%)	4 (13%)	7 (12%)
Black, African, Caribbean or Black British	1 (3%)	0 (0%)	1 (2%)
Other ethnic group	0 (0%)	1 (3%)	1 (2%)
Highest level of completed education, *n* (%)			
Postgraduate degree	14 (47%)	11 (37%)	25 (42%)
Undergraduate degree	10 (33%)	15 (50%)	25 (42%)
Other	6 (20%)	3 (10%)	9 (15%)
Missing	0 (0%)	1 (3%)	1 (2%)
Monthly take-home household income, *n* (%)			
0−£1,999	8 (27%)	6 (20%)	14 (23%)
£2,000−£3,999	7 (23%)	10 (33%)	17 (28%)
£4,000−£6,000	6 (20%)	8 (27%)	14 (23%)
>£6,000	6 (20%)	4 (13%)	10 (17%)
Declined to disclose	3 (10%)	2 (7%)	5 (8%)
Employment category, *n* (%)			
Craft and related trades workers	4 (13%)	3 (10%)	7 (12%)
Elementary occupation	1 (3%)	2 (7%)	3 (5%)
Managers	2 (7%)	0 (0%)	2 (3%)
Professional	17 (57%)	21 (70%)	38 (63%)
Service and sales workers	3 (10%)	2 (7%)	5 (8%)
Skilled agricultural forestry and fishery workers	1 (3%)	0 (0%)	1 (2%)
Technicians and associate professionals	2 (7%)	2 (7%)	4 (7%)
Current occupational status, *n* (%)			
Full-time employed	11 (37%)	15 (50%)	26 (43%)
Part-time employed	10 (33%)	5 (17%)	15 (25%)
Unemployed	4 (13%)	5 (17%)	9 (15%)
Other	5 (17%)	5 (17%)	10 (17%)
Sexual orientation			
Heterosexual	25 (83%)	26 (87%)	51 (85%)
Gay or lesbian	5 (17%)	4 (13%)	9 (15%)
Bisexual	0 (0%)	0 (0%)	0 (0%)
Other	0 (0%)	0 (0%)	0 (0%)
1st degree family history of depression, *n* (%)			
No	15 (50%)	12 (40%)	27 (45%)
Yes	15 (50%)	18 (60%)	33 (55%)
Years spent with feelings of significant depression, mean (s.d.)	19.2 (10.3)	20.9 (10.2)	20.0 (10.2)
Treatment resistance: MSM score at screening, median (IQR)	8.0 (7.0−9.0)	8.0 (7.0−10.0)	8.0 (7.0−9.5)

### Primary outcomes

Primary feasibility parameters are summarized in Table 2. No success metrics were set, but eligibility and randomization rates were high. Of 75 participants consented and screened, 62 were eligible (83%, 95% confidence interval (CI): 72−90%), and 60 were randomized (80%, 95% CI: 69−88%). All randomized participants attended the dosing visit, and 59 of 60 participants completed the MADRS at each timepoint, as the only missing observation was the one participant in the placebo arm who had withdrawn prior to week 1. At week 3, the between-group difference in the MADRS was −10.41 (95% CI: −14.86 to −5.95), indicating a clinically significant improvement in depression in the psilocybin arm compared to the placebo arm.

**Table 2:** Estimates of PsiDeR primary feasibility parameters

Feasibility parameter		Numbers	Proportion (95% CI)
Participation rate (no. enrolled / no. approached)		75 / 165	46% (38−53%)
Eligibility rate (no. eligible / no. consented to screening)		62 / 75	83% (72−90%)
Randomization rate (no. randomized / no. consented)		60 / 75	80% (69−88%)
Dosing visit attendance	Placebo	30 / 30	100% (88%)
	Psilocybin	30 / 30	100% (88%)
	Overall	60 / 60	100% (94%)
Dosing receipt	Placebo	30 / 30	100% (88%)
	Psilocybin	30 / 30	100% (88%)
	Overall	60 / 60	100% (94%)
Week 1 MADRS completion	Placebo	29 / 30	97% (83−100%)
	Psilocybin	30 /30	100% (88%)
	Overall	59 / 60	98% (91−100%)
Week 3 MADRS completion	Placebo	29 / 30	97% (83−100%)
	Psilocybin	30 / 30	100% (88%)
	Overall	59 / 60	98% (91−100%)
Week 6 MADRS completion	Placebo	29 / 30	97% (83−100%)
	Psilocybin	30 / 30	100% (88%)
	Overall	59 / 60	98% (91−100%)

The 97.5% one-sided CI (with an upper bound of infinity) is given if the proportion was 100%.

The participation rate is calculated as the number enrolled (consented and screened face to face) as a proportion of the number approached (screened by a researcher over the telephone).

PsiDeR, Psilocybin in Depression Resistant to Standard Treatments.

### Secondary outcomes

Clinical outcomes at baseline and follow-up are summarized in Supplementary Table 1. Baseline scores were balanced between arms. Three responses to baseline Euro-QoL 5D-5L Visual Analog Scale (EQ-5D-5L VAS) and Maudsley 3-item Visual Analog Scale (M3VAS) scores and four responses to the baseline Warwick-Edinburgh Mental Well-being Scale (WEMWBS) were missing; these values were mean imputed prior to analyses in mixed linear regression models. No multiple imputation was done for missing follow-up measurements.

At week 3, 1 (3%) participant in the placebo arm and 13 (43%) participants in the psilocybin arm met the cutoff for MADRS response, and 1 (3%) in the placebo arm and 12 (40%) in the psilocybin arm met the cutoff for MADRS remission. These figures were sustained at week 6; however MADRS response increased to 15 (50%) participants in the psilocybin arm. At week 3, no participants in the placebo arm met criteria for Quick Inventory of Depressive Symptomatology Self-Report 16-item version (QIDS-SR-16) response compared to 15 (50%) in the psilocybin arm. At week 6, one (4%) participant in the placebo arm and 13 (43%) participants in the psilocybin arm met QIDS-SR-16 response. One participant (4%) in the placebo arm and 12 (40%) participants in the psilocybin arm met the cutoff for QIDS-SR-16 remission at week 3 compared to two (7%) in the placebo arm and 10 (33%) in the psilocybin arm at week 6. Plots of raw MADRS and QIDS-SR-16 scores over time, as well as predicted scores from mixed models, are displayed in Supplementary Figs. 1 and 2.

There were more reports of moderate anxiety on the Generalized Anxiety Disorder Scale (GAD-7) in the placebo arm, with lower proportions observed in the psilocybin arm: at week 6, 5 of 30 (17%) psilocybin participants had GAD-7 scores ≥10 compared with 18 out of 29 (62%) placebo participants. Owing to the low number of events and small sample, the logistic regression estimates of odds ratios were extremely imprecise (Supplementary Table 2).

**Fig. 2:**
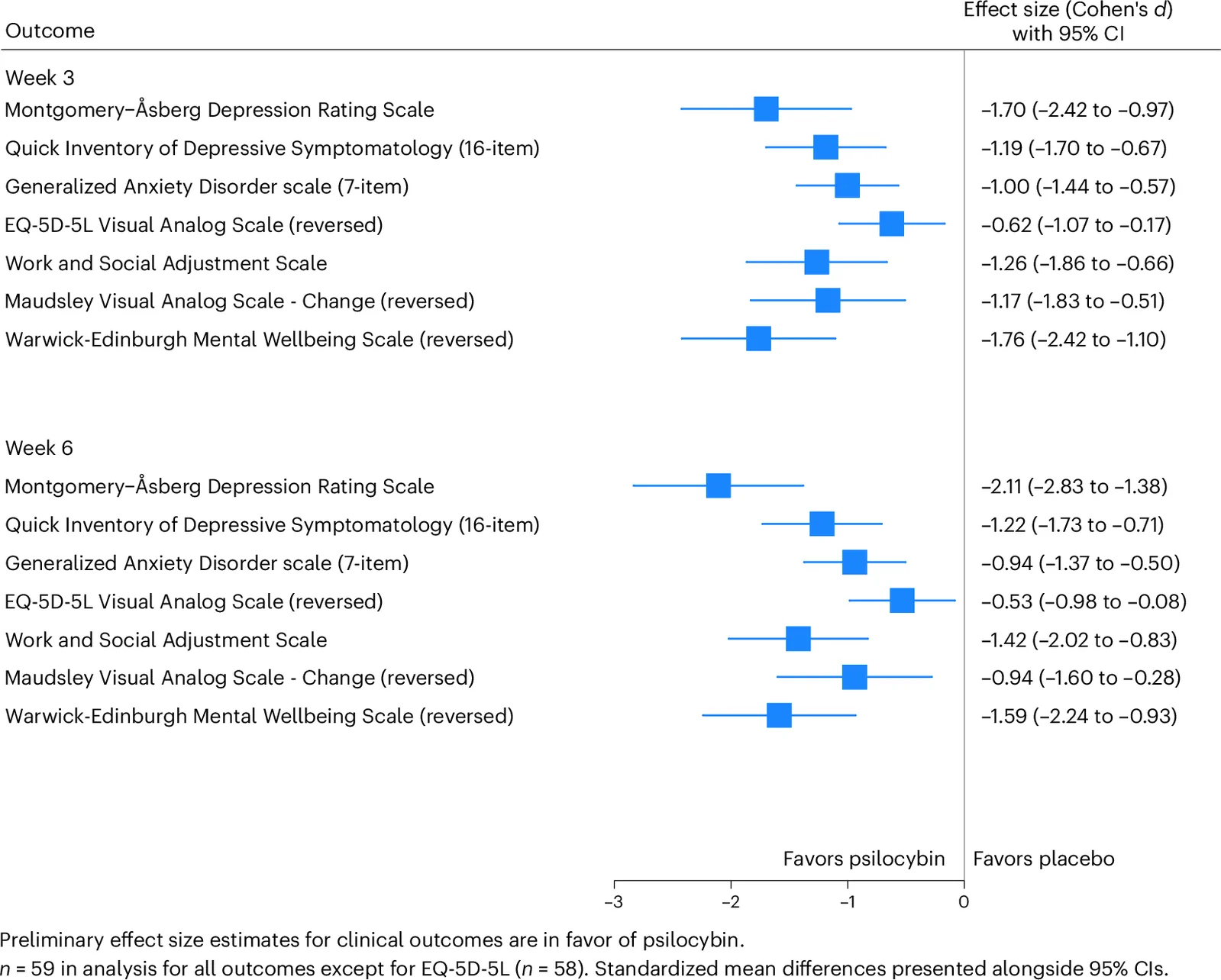
Primary and secondary outcomes. Data presented as standardized effect sizes (Cohen’s *d*) at week 3 (top) and week 6 (bottom).

Adjusted mean differences between arms and associated Cohen’s *d* values for secondary clinical outcomes are presented in Table 3. Standardized effect sizes at week 3 and week 6 are presented in Fig. 2. Large effect sizes were observed for clinician-reported and participant-reported measures of depression severity. The between-group difference in the MADRS at week 3 was sustained at week 6, with an adjusted mean difference of −12.92 points (95% CI: −17.37 to −8.47). Self-reported depression severity was improved in the psilocybin arm compared to placebo at all follow-up timepoints, as were ratings of anxiety, overall health state, psychopathology and mental wellbeing. M3VAS total change scores were higher and improved at all follow-up timepoints in the psilocybin arm.

**Table 3:** Preliminary effect sizes estimates for PsiDeR secondary clinical outcomes

Secondary clinical scale (*n* = observations in mixed model)	Timepoint	Mean difference (95% CI)	Cohen’s *d* (95% CI)
MADRS (*n* = 59)	Week 1	−11.44 (−15.89 to −6.98)	−1.87 (−2.59 to −1.14)
	Week 3	−10.41 (−14.86 to −5.95)	−1.7 (−2.42 to −0.97)
	Week 6	−12.92 (−17.37 to −8.47)	−2.11 (−2.83 to −1.38)
QIDS-SR-16 (*n* = 59)	Week 1	−5.85 (−8.20 to −3.50)	−1.27 (−1.78 to −0.76)
	Week 3	−5.48 (−7.85 to −3.12)	−1.19 (−1.7 to −0.67)
	Week 6	−5.63 (−8.00 to −3.27)	−1.22 (−1.73 to −0.71)
GAD-7 (*n* = 59)	Week 1	−4.93 (−7.03 to −2.83)	−1.02 (−1.45 to −0.58)
	Week 3	−4.85 (−6.96 to −2.74)	−1.00 (−1.44 to −0.57)
	Week 6	−4.54 (−6.64 to −2.44)	−0.94 (−1.37 to −0.50)
EQ-5D-5L VAS (*n* = 58)	Week 3	12.46 (3.39−21.54)	0.62 (0.17−1.07)
	Week 6	10.69 (1.63−19.75)	0.53 (0.08−0.98)
WSAS (*n* = 59)	Week 3	−8.20 (−12.09 to −4.30)	−1.26 (−1.86 to −0.66)
	Week 6	−9.22 (−13.08 to −5.36)	−1.42 (−2.02 to −0.83)
M3VAS (*n* = 59)	Day 1	30.07 (4.75−55.38)	0.78 (0.12−1.43)
	Week 1	44.68 (19.26−70.09)	1.15 (0.50−1.81)
	Week 3	45.33 (19.69−70.96)	1.17 (0.51−1.83)
	Week 6	36.42 (10.80−62.04)	0.94 (0.28−1.60)
WEMWBS (*n* = 59)	Week 3	10.48 (6.57−14.40)	1.76 (1.10−2.42)
	Week 6	9.44 (5.55−13.32)	1.59 (0.93−2.24)

Estimates are from mixed models adjusting for the fixed effects of arm, time (categorical), arm × time, design factors (age group, sex, past psilocybin use) and baseline values of outcomes plus random intercepts at the participant level. Cohen’s *d* is the mean difference between arms divided by pooled baseline standard deviation.

PsiDeR, Psilocybin in Depression Resistant to Standard Treatments.

### Safety

In total, 287 nonserious AEs (123 in the placebo arm, 164 in the psilocybin arm) were recorded in the RCT phase (Table 4). Nonserious AEs occurring over the entire trial (RCT phase and open-label phase) are listed in Supplementary Table 6. Four SAEs (one in the placebo arm, three in the psilocybin arm) were recorded: three during the open-label phase and one during the RCT phase (Supplementary Table 3). Over half of nonserious AEs (184 out of 287, 64%) were not related to study medication, and all but one SAE was deemed not related to study medication. The remaining event, which occurred in the placebo arm, was deemed as possibly related. One participant withdrew from the placebo arm; the participant no longer wished to participate for personal reasons. Safety and mechanism scales are summarized in Supplementary Tables 4 and 5, respectively.

**Table 4:** Nonserious AEs by arm and overall for events reported during the main phase (randomization to week 6)

	Placebo *N* events = 123	Psilocybin *N* events = 164	Overall *N* events = 287
	(reporters = 28)	(reporters = 30)	(reporters = 58)
Body system category			
Neurological	32 (17)	53 (26)	85 (43)
Mood/psychological	13 (9)	30 (18)	43 (27)
Respiratory infection	24 (16)	12 (11)	36 (27)
Gastrointestinal	8 (6)	15 (12)	23 (18)
Musculoskeletal/pain	13 (11)	14 (9)	27 (20)
Reproductive	2 (2)	3 (2)	5 (4)
Skin/ocular	3 (3)	2 (2)	5 (5)
Medication or substance related	5 (5)	10 (9)	15 (14)
General fatigue/tiredness	4 (4)	8 (8)	12 (12)
Other	19 (11)	17 (12)	36 (23)
Other event details			
Relatedness, *n* (%)			
Not related	105 (85%)	79 (48%)	184 (64%)
Possibly related	16 (13%)	56 (34%)	72 (25%)
Related	2 (2%)	29 (18%)	31 (11%)
Intensity, *n* (%)			
Mild	77 (63%)	111 (68%)	188 (66%)
Moderate	45 (37%)	48 (29%)	93 (32%)
Severe	1 (1%)	5 (3%)	6 (2%)
Outcome during main phase, *n* (%)			
Resolved during main phase	101 (82%)	143 (87%)	244 (85%)
Unresolved during main phase	18 (15%)	10 (6%)	28 (10%)
Ongoing	4 (3%)	11 (7%)	15 (5%)
Action taken, *n* (%)			
None	63 (51%)	113 (69%)	176 (61%)
Medication required	56 (46%)	48 (29%)	104 (36%)
Other	4 (3%)	3 (2%)	7 (2%)
Adverse events of special interest (AESIs), *n* (%)			
Not AESI	123 (100%)	164 (100%)	287 (100%)
AESI	0 (0%)	0 (0%)	0 (0%)

For body system categories, the number of events of that category is reported first, followed by the number of unique reporters in parentheses.

Only the nonserious AEs reported during the main phase (randomization to week 6) are summarized here, as participants remaining for the open-label phase all received psilocybin. The outcome at the time of the week 6 visit is reported here (that is, ‘Unresolved during main phase’, meaning that the event started during the main phase but was not resolved until the open-label phase, and ‘Resolved during main phase’, meaning that the event was reported and resolved during the main phase).

### Post hoc analysis

Of the 23 participants who were asked, *n* = 13 out of 13 (100%) in the psilocybin arm and *n* = 7 out of 10 (70%) in the placebo arm correctly guessed their treatment allocation.

## Discussion

This study of psilocybin assisted-therapy for TRD—to our knowledge, the first RCT in this population to assess feasibility in an NHS setting in England—found significantly greater improvements in depressive symptoms in the active treatment arm compared to placebo, maintained at 3-week and 6-week follow-up. Psilocybin administration was well tolerated: AEs were mostly mild in severity and resolved by the end of the trial, and clinical safety scales measuring suicidality and mania did not show substantial changes across time. SAEs in all cases where a participant received psilocybin were deemed unrelated to the treatment. Recruitment, retention and measure completion metrics supported the feasibility of the trial design.

We noted a very large effect size at week 3, which was sustained at week 6 for both clinician-rated and patient-rated depressive symptoms. However, a part of this effect is likely attributable to a lack of response or remission in the placebo arm, with total MADRS and QIDS-SR scores not showing meaningful reductions across time. Blinding was not upheld, with 100% of participants who received psilocybin and 70% of participants who received placebo correctly guessing their allocation. Thus, expectancy effects in both groups likely account for some of the between-group difference observed.

These findings complement two previous RCTs of psilocybin versus placebo or immediate versus delayed treatment in MDD and TRD, respectively, both of which found very large between-group effect sizes at week 2 on the MADRS^13,16^. Together, these results suggest that a single dose of psilocybin may reduce depressive symptoms in MDD and TRD, although larger, and longer, scale studies are required to confirm the nature and durability of these effects.

On average, patients in this trial had been ill for 20 years and showed moderate levels of treatment resistance. Unlike commercial trials in this population^15^, we did not set an upper limit on the degree of treatment resistance, thus including some participants with severe and chronic illness who had not responded to a wide range of interventions. This may be more representative of a real-world implementation of psilocybin therapy, particularly in healthcare settings such as the NHS, where novel interventions are usually reserved for those unresponsive to multiple established treatments.

This trial was delivered initially in an NHS clinical research facility. Halfway through recruitment, we moved to a refurbished community mental health team setting in a residential area, close to the local hospital. Although this move was dictated by circumstance rather than intention, it introduced a natural test of the deliverability of psilocybin therapy in a less regulated, outpatient community setting. Indeed, such a setting is generally thought to be preferable for treatments involving drugs such as psilocybin, where overtly medical or inpatient hospital settings can invoke unhelpful health anxiety and are probably unnecessary given the benign physiological toxicity profile of the drug itself. Our own observations underlined this: we noted no new logistical or safety concerns because of the move; however, we present this as informal commentary only.

An inactive placebo control allows for a better characterization of safety signals in psychedelic trials and does not adversely affect feasibility aims; hence, it was chosen for this trial^17^. However, other trial designs (that is, active placebos or subperceptual doses of the study drug) may be more appropriate for efficacy trials and will be considered in the design of a future trial^17^. Indeed, in the largest RCT of psilocybin for TRD to date, Goodwin et al.^15^ found more modest effect sizes comparing 1-mg to 25-mg psilocybin at week 3 than in the present trial.

In the present study, the proportion of patients reporting suicidal ideation on the Columbia−Suicide Severity Rating Scale (C−SSRS) was similar at baseline and at week 3 and week 6 follow-up, after an initial reduction at day 1 and week 1. Proportions also appeared similar across arms. Conversely, suicidality as measured by the M3VAS appeared to improve in the psilocybin arm at each follow-up point (by approximately 15 points on average at week 3 and week 6) and remained relatively stable or worsened slightly in the placebo arm (by approximately −2 to −6 points on average). The M3VAS may provide a more nuanced measurement of change over time compared to the C−SSRS rating. High suicidal risk as measured by the C−SSRS was an exclusion in this trial (as is a usual requirement for ethical authorizations of experimental drug treatments in psychiatry), and, thus, the potential for observable change was attenuated.

Concerns have been raised over suicidality in previous psilocybin trials, with suicidal ideation and behavior AEs and SAEs appearing more common in the 10-mg or 25-mg arms than in the 1-mg arm in the trial reported by Goodwin et al.^15^, although statistical significance was not assessed^15^. On the other hand, a review including three RCTs of psilocybin-assisted therapy that included suicidal ideation as an outcome or safety measure reported significant reductions in suicidal ideation with psilocybin^18^. A 2-year follow-up of participants in this trial is currently underway and will be reported in due course.

### Limitations

This trial has several strengths and limitations. Given that this trial was implemented within a public mental healthcare setting in the NHS in the UK, it helps inform how psilocybin therapy may be implemented within this healthcare context, if licensing is achieved and funding models are agreed. The recruitment target was reached, and almost all participants were retained to week 6, indicating that the inclusion criteria were appropriate and study procedures were acceptable to the population. These aspects of study design may be used in future efficacy trials in this setting.

Additional strengths include the use of a true placebo design that allows a baseline rate of AEs to be collected (to our knowledge, only one previous RCT in MDD has used a true placebo^13^); the inclusion of an optional open-label extension for all participants, which may have contributed to the high rates of participant retention; and the wide inclusion criteria (lack of an upper limit for treatment resistance as well as including psychological therapy trials), which allowed for a more ‘real-world’ group of patients to be recruited than the usual narrow criteria seen in commercially funded trials.

Ethnic diversity has been reported to be poor in clinical trials of psychedelics, with 85% of participants across studies being white^19^. Although results of trials testing psilocybin for various conditions have been promising, limited participation of ethnic minorities in these trials limits generalizability of the results. Furthermore, best practices in delivering complex interventions such as these may differ across individuals with different cultural backgrounds. We largely met our recruitment targets in all ethnic groups, apart from in Black, African, Caribbean or Black British participants. This is an important limitation that we aim to address in the follow-on trial; this could involve increased recruitment of individuals from Black ethnic groups as researchers or clinicians to increase cultural competency as well as inclusion of relevant cultural centers in participant recruitment efforts^20^.

In a similar way, we also aimed to recruit a diverse sample with regard to sexual orientation, surpassing our target for recruiting gay or lesbian participants but underrecruiting bisexual or other orientations. We did not create targets for recruitment for educational level or employment; this is another important limitation that could be addressed in future trials using similar methodology used for ethnicity and sexual orientation. We note that our sample population is highly educated, with much higher proportions of participants with university degrees than in the general population.

Functional unblinding due to the subjective effects of psilocybin is an obvious limitation of this trial, as it is with all trials of drugs that have noticeable effects, as well as many psychological and behavioral interventions. However, we used raters who were independent of the trial team to measure the primary outcome in this trial, and participants were asked not to discuss their beliefs about their allocation with the raters. Despite this, the limited assessment of blinding is a weakness of this trial. However, it is important to note that expectancies and functional unblinding in psychedelic trials are not solely a source of bias. The therapeutic model of psilocybin-assisted therapy relies on the subjective drug effects and their integration; therefore, expectancies, subjective effects and pharmacological effects likely work synergistically and thus may be intrinsic to the treatment^21^. Although placebo-controlled RCTs continue to be considered ‘gold standard’ to the extent that they are demanded for regulatory approval, they have obvious limitations when applied to psychoactive drugs delivered within a supportive context. A more detailed understanding of psilocybin therapy will necessarily emerge from a confluence of controlled and observational studies; statistical approaches that incorporate prior knowledge (for example, Bayesian); and, perhaps most critically, the sort of nuanced insights that arise from extensive clinical experience and careful attention to patient feedback.

The results of this trial suggest that administering a single dose of psilocybin versus placebo to participants with TRD, with psychological support, in a randomized, double-blind trial design is feasible. The treatment was well tolerated, and secondary endpoints suggest improvements in other important outcomes, such as anxiety. Efficacy and duration of antidepressant effects were observed up to 6 weeks in this trial using the preregistered, independently collected primary outcome. Feasibility parameters support the development of larger efficacy trials in public mental health settings in this population. However, the placebo control design alongside the obvious functional unblinding that occurs with psilocybin introduces complexity into the interpretation of results. Designs that are better able to disambiguate between the differential expectancy effects between groups and the interactional effect of psychological support given alongside a drug intervention are required, although such disambiguation is unlikely to be definitive in any clinically meaningful sense. Future trials should also examine long-term effects of psilocybin on functional outcomes. Furthermore, the cost-effectiveness of psilocybin therapy should be adequately characterized if it is to be assessed for provision in a public healthcare setting.

If found to be clinically effective and cost-effective in larger trials, psilocybin could offer a new treatment for TRD—a common and chronic condition with a high burden of disability and disproportionate risk of completed suicide, with few licensed or effective treatments available. In the longer term, this could lead to substantial improvements in functional recovery and reductions in societal and healthcare costs.

## Methods

### Study design

This was a parallel-group, two arm, double-blind, randomized, placebo-controlled phase 2 feasibility trial undertaken at one NHS site in the UK (South London and Maudsley NHS Foundation Trust). An open-label extension was also offered to all participants, lasting a further 12 weeks after the 6-week RCT follow-up period. Study visits were carried out initially at King’s College Hospital Clinical Research Facility and, subsequently, a refurbished community mental healthcare and research setting in the local community.

The trial received a favorable opinion from the London, Brent Research Ethics Committee (reference 20:/LO/0206). The trial was registered with the Medicines and Healthcare Products Regulatory Agency (EudraCT no.: 2018-003573-97) and at ClinicalTrials.gov (NCT04959253), and the trial protocol was previously published^22^. The sponsor of the trial was the South London and Maudsley NHS Foundation Trust and King’s College London. COMP360 psilocybin—a proprietary, pharmaceutical-grade synthetic psilocybin formulation—and matching placebo capsules were supplied by COMPASS Pathfinder Ltd., a subsidiary of COMPASS Pathways plc, which had no role in the design, conduct or analysis of the study.

Patients with lived experience (including the Service User Research Enterprise at King’s College London) helped develop the trial. Patients were also recruited to the trial steering committee and were involved in trial oversight.

### Participants

The target sample size was 60 participants. Recruitment to this trial was severely delayed by the impact of the COVID-19 pandemic, although the trial did recruit to target. Participants were recruited via primary and secondary care services, established registries of research-interested patients and advertising in the community and social media. We aimed to recruit a representative sample with regard to ethnicity and sexual orientation, using UK census data to create targets for participant recruitment. Because there are differences in demographics between London and the rest of the UK, and we expected participants from both within and outside of London, targets were based on the average of the London population and the rest of the UK.

Eligibility criteria for participants included age 25−80 years, fluency in English, meeting DSM-5 criteria for a current episode of MDD, a 17-item Hamilton Depression Rating Scale (HAM-D-17)^23^ score of ≥14 and an inadequate response to ≥2 therapeutic antidepressant trials or ≥1 antidepressant trial and ≥1 psychotherapy trial. There was no upper limit on the number or type of previous treatments permitted. For those aged ≥60 years, the first episode of depression must have started prior to their 60th birthday. Exclusion criteria included a DSM-5 diagnosis of bipolar disorder, psychotic disorder, drug or alcohol dependence syndrome, personality disorder or dementia; suicide attempt in the past year; any clinically significant medical diagnoses or biochemical or electrocardiographic abnormalities deemed incompatible with psilocybin treatment; personal circumstances or behavior judged to be incompatible with establishment of rapport or safe exposure to psilocybin; women of childbearing potential not using adequate contraception; women who were pregnant or breastfeeding; those unable to give informed consent; those enrolled in another drug trial; and hypersensitivity to the investigational medicinal product, any of the excipients or placebo. The trial was undertaken with licenses from the UK Home Office to possess and administer Schedule 1 controlled drugs to human subjects. All participants gave written informed consent. Participants were not paid for participation in this trial; however, they were reimbursed for out-of-pocket expenses.

### Randomization and masking

Participants were randomized (1:1) to receive 25-mg psilocybin or placebo, with the sequence generated using minimization by sex (male, female), age (25−59, ≥60) and past psilocybin use (yes, no), by a web-based service hosted by the King’s Clinical Trials Unit (KCTU) and concealed from the trial team. Participants, researchers and the senior statistician (B.C.) were masked to treatment allocation until database lock. The trial statistician (P.C.) was kept masked until approval of the statistical analysis plan (SAP). During enrollment, the treatment allocation was sent electronically from the KCTU system to the hospital pharmacy, which dispensed the blinded treatment to a member of the trial team. COMP360 psilocybin and placebo were administered as five capsules, matched for appearance, weight, color, texture and smell. The adequacy of participant blinding was assessed using self-report visual analog scales of group allocation confidence, introduced part-way through the trial and, therefore, collected in only a subsample of participants.

### Procedures

Participants determined eligible at screening (V1) entered a screening and preparation phase (lasting 1−8 weeks), during which they were withdrawn from antidepressant, antipsychotic or mood-stabilizing drugs and had sessions of psychological preparation and psychoeducation with a trial therapist. A baseline visit (V2) followed, where final eligibility was confirmed, baseline clinical measures were taken and participants were randomized. One-to-three days later, participants attended a dosing visit (V3). Participants self-administered either 25-mg COMP360 psilocybin or placebo via oral administration. Participants were accompanied by a therapist and chaperone for approximately 6 hours within a quiet room with a bed and adjustable lighting within the research facility. Participants were given an optional eye mask and earphones through which they listened to a curated playlist of music. A trial doctor determined suitability for discharge at the end of the day. For 6 weeks after the dosing visit, participants met with the study team to complete follow-up assessments and engage in time-limited integration therapy (1 day (V4), 1 week (V5), 3 weeks (V6) and 6 weeks (V7) after dosing). As part of the extended design of this trial, at 6-week follow-up all eligible participants were invited to take part in a 12-week open-label extension, the results of which will be published elsewhere. Additional details on the psychological support given throughout the trial, as well as measures completed at each visit, are available in the protocol^22^.

### Outcomes

Primary outcomes were measures of feasibility: recruitment rates, dropout rates and an estimate of the variance of the MADRS^24^. Secondary outcomes at weeks 1, 3 and 6 follow-up included MADRS total scores; proportions of participants in each group who met criteria for clinician-rated response to treatment (defined as a ≥50% decrease in MADRS score from baseline) and remission (defined as a MADRS score ≤10); QIDS-SR-16 (ref. ^25^) total scores; and proportions of participants in each group who met criteria for response to treatment (defined as a ≥50 decrease in QIDS-SR-16 score from baseline) and remission (defined as QIDS-SR-16 score ≤5). Additional clinical outcomes reported here include the GAD-7 (ref. ^26^), ratings of overall health state from the EQ-5D-5L VAS^27^, Work and Social Adjustment Scale (WSAS)^28^, M3VAS^29^ and the WEMWBS^30^ at 1-week, 3-week and 6-week follow-up.

AE and SAE data were collected. Other safety measures reported here include the C−SSRS^31^, the Young Mania Rating Scale (YMRS)^32^, the Altman Self-Rating Mania Scale (ASRM)^33^ and the Positive and Negative Affect Scale (PANAS)^34^. An exploratory measure was created for this study: Delayed Psychedelic After Effects Scale (DePAS). Mechanism outcomes were also collected using clinical scales: State Trait Anxiety Inventory (STAI)^35^, 5-Dimension Altered States of Consciousness Questionnaire (5D-ASC)^36^ and item scores on the M3VAS.

### Statistical analysis

The SAP was drafted following KCTU standard operating procedures by the senior and trial statisticians, both blinded to allocation (Supplementary Information). The SAP was reviewed and approved by the senior trial statistician (B.C.), the principal investigator (J.J.R.) and the independent trial steering committee statistician before database lock. The study was designed as a feasibility study to estimate the dropout rate (20%) with a 95% CI of ±10.1% (9.9−30.1%). No hypothesis testing was undertaken.

Analyses were intention to treat: all participants were analyzed as randomized. To evaluate recruitment and other feasibility parameters, the following are reported alongside 95% CIs: proportion eligible out of number screened; proportion randomized out of number consented; proportion completing at least the MADRS at each follow-up visit out of number randomized (overall and by arm); proportion attending the dosing visit out of number randomized (overall and by arm); and proportion receiving all five capsules/tablets out of number randomized (overall and by arm). Completeness of outcome measures and mean (s.d.) or median (IQR) scores for each outcome measure at baseline and each follow-up are reported.

For secondary continuous clinical outcomes, the adjusted mean difference between arms was estimated in mixed-effects linear regression models. Follow-up values were fit simultaneously and predicted by the fixed effects of trial arm, baseline assessment, minimization factors (sex, age group, prior psilocybin use), time (categorical) and an arm × time interaction term to allow estimates of the treatment effect at each follow-up point. A random intercept was fit at the participant level. Standardized effect sizes (Cohen’s *d*) were calculated by dividing the mean difference between arms over the pooled baseline standard deviation; 95% CIs were reported alongside adjusted mean differences (aMDs) and Cohen’s *d*.

Secondary binary outcomes were analyzed using mixed-effects logistic regression models with random intercept at the participant level and adjusting for the fixed effects, as stated previously. Marginal odds ratios and 95% CIs are reported. Although the GAD-7 at week 3 was only specified in the protocol as an exploratory endpoint, we also compared the odds of reporting moderate anxiety (GAD-7 ≥10) between arms at each follow-up timepoint using the same method. If there were few events, only descriptive summaries are presented with alternative options explored and reported in Supplementary Table 2 (that is, Firth-type logistic regression with intercept correction).

AE and SAEs are summarized overall and by arm using counts of events and participants reporting events. Safety and mechanism scales are summarized descriptively.

Stata version 18.0 was used for analyses. A trial steering committee with an embedded independent data monitoring and ethics committee oversaw the trial. Trial procedures and processes were monitored by the King’s Health Partners Clinical Trials Office.

### Reporting summary

Further information on research design is available in the Nature Portfolio Reporting Summary linked to this article.

## Online content

Any methods, additional references, Nature Portfolio reporting summaries, source data, extended data, supplementary information, acknowledgements, peer review information; details of author contributions and competing interests; and statements of data and code availability are available at https://doi.org/10.1038/s41591-026-04541-0.

## Supplementary information

Supplementary Information Supplementary text, including Supplementary Figs. 1−3 and Supplementary Tables 1−6.

Reporting Summary

## Data Availability

Access to deidentified participant data may be available to researchers who provide a statistically coherent proposal to the corresponding author, beginning 9 months after publication, for 36 months. By ‘statistically coherent’, we mean that the data requested might reasonably be expected to answer the question asked (either alone or in aggregate with other datasets) with a reasonable chance of statistical precision.

## Code availability

Code used for analyses can be found at https://github.com/petrinachu/psider.
