# Supplementary material for: Psilocybin-assisted therapy for treatment-resistant major depressive disorder in a public healthcare setting: a randomized controlled trial
Source: Nat Med. 2026 Aug 6;32(9):3430–7. doi: 10.1038/s41591-026-04541-0 (PMC13577891; doi:10.1038/s41591-026-04541-0)
Supplement: Supplementary file 1 [file 41591_2026_4541_MOESM1_ESM.pdf]

nature medicine

Supplementary information https://doi.org/10.1038/s41591-026-04541-0

Psilocybin-assisted therapy for treatment-resistant major depressive disorder in a public healthcare setting: a randomized controlled trial

In the format provided by the authors and unedited

Table of Contents

PsiDeR Study Group Members 5

Statistical Analysis Plan 7

Secondary clinical outcomes 26

Supplementary table S1. Summary of secondary clinical outcomes at baseline and follow-up .. 26

Supplementary figure 1: Profile plots of raw depression severity scores 28

Supplementary figure 2. Predicted depression scores from mixed models. 29

Supplementary table S2: Results of logistic regression analyses for secondary clinical binary outcomes 30

Serious Adverse Events 31

Supplementary table S3: Serious adverse event listing 31

Safety Scales.... 32

Supplementary table S4: Summary of safety scales.... 32

Mechanism Scales.... 34

Supplementary table S5: Summary of mechanism scales 34

Adverse Events During RCT and Open-Label Phase 35

Supplementary table S6: Summary of non-serious adverse events during RCT and open-label phase.... 35

Supplementary figure 3: Adverse event categories by arm 36

Protocol Full Title.... 38

Protocol Short Title/Acronym 38

Trial Identifiers.... 38

Sponsor/Co-Sponsor: 38

Chief Investigator.... 38

Co-Investigator(s).... 38

Study Synopsis 44

Glossary of Terms 46

Background & Rationale.... 50

Need 50

Background & Rationale.... 50

Pilot Work.... 51

Trial Description 52

Trial Design.... 53

Recruitment 53

Screening 53

Trial Flowchart & Structure 56
Screening & Preparation (SP) Phase (1-8 weeks) 58
Baseline Visit (V2) 60
Dosing Visit (Treatment) (V3) 60
Follow Up Phase (6 weeks) 62
Follow Up Visit 1 (V4). 1 Day After Treatment. 62
Follow Up Visit 2 (V5). 1 Week After Treatment. 63
Follow Up Visit 3 (V6). 3 Weeks After Treatment. 63
Follow Up Visit 4 (V7). 6 Weeks After Treatment. 63
Open-Label Extension. 64
Transfer of Care Arrangements 65
Escape Criteria and Trial Withdrawal 65
Trial Objectives and Design 66
Trial Purpose 66
Trial Objectives 66
Primary Objective 66
Secondary Objectives 66
Exploratory Objectives 67
Trial Endpoints 67
Primary Endpoint 67
Secondary Endpoints 67
Exploratory Endpoints 67
Trial Design 67
Trial Flowchart 67
Trial Medication 69
Investigational Medicinal Product (IMP) 69
Summary of IMP Basic Chemical Properties 69
IMP Manufacture 69
IMP Stability & Shelf Life 70
Dosing Regimen 70
IMP Risks 70
Pharmacology 70
Reported Experiences with Psilocybin 71
Reported-Observed Findings in the Literature Coded Using MedDRA 71
Psychological and Subjective Effects 72
Safety Data Collection 74

Acute Adverse Events 76
Risky or Reckless Behaviour 76
Delayed Adverse Events 77
Prolonged Psychotic Reactions 78
Hallucinogen Persisting Perception Disorder (HPPD) 78
Chronic Neurotoxicity & Personality Change 79
Abuse & Dependence Liability 80
Damage to Non-Brain Organs 80
Fatalities 81
Serotonin Syndrome 82
Legal Position 82
Drug Accountability 83
Storage of IMP 83
Subject Compliance. 83
Concomitant Medication 83
End of Trial 84
Selection of Subjects 85
Inclusion Criteria 85
Exclusion Criteria 85
Exclusions for Pre-Existing Medical Conditions 86
Women of Child Bearing Potential 86
Recruitment of Participants 87
Randomisation Procedure 87
Implementation 88
Emergency Code Break 88
Withdrawal of Subjects 88
Expected Duration of Trial 88
Trial Procedures 90
Trial Measures & Procedures 90
List of Measures & Procedures 90
Schedule of Measures & Procedures 92
Allowable Windows 93
Description of Study Team Administered Measures 93
Description of Participant Administered Measures 98
Open Label Extension (OLE) 103
Protocol Deviations 104

Assessment of Efficacy 105
Primary Efficacy Parameters 105
Secondary Efficacy Parameters 105
Assessment of Safety 106
Specification, Timing and Recording of Safety Parameters. 106
Procedures for Recording and Reporting Adverse Events 107
Reporting Responsibilities 108
Documentation, Classification & Follow Up of Adverse Events 108
Other Procedures for Reporting and Recording Adverse Events 110
Adverse Events of Special Interest (AESI) 110
Adverse Events That Do Not Require Reporting 111
Treatment Stopping Rules 111
Statistics 112
Sample Size 112
Randomisation 112
Analysis 112
Trial and Intervention Feasibility Parameters 112
Estimates for Effect Sizes for Psilocybin 25mg vs. Placebo. 113
Trial Steering Committee 114
Data Monitoring Committee 115
Direct Access to Source Data and Documents 116
Ethics & Regulatory Approvals 117
Quality Assurance 118
Data Handling 119
Data Management 120
Publication Policy 121
Insurance / Indemnity 122
Financial Aspects 123
Signatures 124
References 125

PsiDeR Study Group Members

Therapists:

Amy Durden

Carolina Maggio

Chris Partridge

David Isaacs

Felicity Runchman

Gemma Knight

Joe Kibble

Katie Turnbull

Raquel Scheid

Rebecca Samuels

Sara Tookey

Steve Burchell

Chaperones:

Werner Kierski

Debbie Johnson

Komal Patel

Tonya Bathe

Timmy Davis

Penny Collins

Cerri Saville

Zsofia Elek

Rebecca Samuels

Rosario Vergara

Website Design and Maintenance:

Michael Bourne

Trial Steering Group & Data Safety Board Members:

Celia Morgan

Winston Banya

Michael Bourne

Ian Roullier

Sarah Markham

Chao Huang

Centre for Mental Health Research and Innovation staff:

Laura Hemmings,

Sophie Willams,

Florence Akello

Additional blinded raters:

Benjamin Gooddy,

Daniel Silman,

Andrea Ulrichsen,

Hans-Christian Stein,

Anna Tröger,

Brandi Eiff,

Romayne Gadelrab

PsiDeR

Psilocybin in Depression Resistant to Standard Treatments (PsiDeR) Trial

A randomised, placebo-controlled trial of psilocybin in treatment resistant depression: a feasibility study

Statistical Analysis Plan

Version 1.0

Started 24 Apr 2024

IRAS Reference Number: 252750

EudraCT Number: 2018-003573-97

REC Number: 20-LO/0206

Written based on protocol v1.20 (08 Feb 2024)

Trial Statistician: Petrina Chu

Signed: Date: 10 Sep 2024

Senior Trial Statistician: Prof Ben Carter

Signed: Date: 10 Sep 2024

Principal Investigator: Dr James Rucker

Signed: Date: 11 SEP 24

Independent TSC Statistician: Dr Chao Huang

Signed: Date: 10/09/2024

1. Description of the trial

PsiDeR is a randomised, placebo-controlled feasibility trial of psilocybin in treatment resistant depression (TRD). This is a study with a central 'core' element and three optional arms (the optional arms are introduced but they are beyond the aim of this SAP). The core element is the randomised controlled trial of psilocybin in TRD in up to 60 completing participants with 6 weeks of follow up. Three 'orbiting' elements (optional) can then be 'bolted on' according to participant wishes/consent. If participants consent to involvement with these optional elements, then they will be free to withdraw at any time without affecting their ability to continue in the core trial.

The optional elements are as follows: 1. NIHR Maudsley BioResource for Mental Health. 2. Neuroimaging. 3. An open-label extension with 1 further dose of 25mg psilocybin after 6 weeks of follow up and 12 weeks of subsequent follow up.

1.1. Study objectives

1.1.1. Primary objective

To evaluate the feasibility of a randomised, controlled trial design, in which a single dose of psilocybin 25mg PO vs placebo, is given to adult participants with treatment resistant major depressive disorder (TRD), under psychologically supportive conditions, with 6 weeks of follow up, by measuring recruitment rates, dropout rates and by estimating the variance of the future primary outcome measure (Montgomery Asberg Depression Rating Scale [MADRS]) to inform upon the design of a phase 3 trial. (See **Section 1.7.1** for more information about MADRS).

1.1.2. Secondary Objectives

The secondary objectives of this trial are as follows:

1. To assess the clinician-rated efficacy of psilocybin 25mg compared to placebo via:

   a) The change in the MADRS total score from Baseline to 3 weeks after treatment.

   b) The proportion of participants who demonstrate a response to treatment, where response to treatment is defined as a ≥50% decrease in MADRS total score from Baseline to Week 3. This will also be assessed at Weeks 1 and 6.

   c) The proportion of participants in remission, where remission is defined as a MADRS total score ≤10 at Week 3. This will also be assessed at Weeks 1 and 6.

2. To assess the participant-rated efficacy of psilocybin 25mg compared to placebo via:

   a) The proportion of participants who demonstrate a response to treatment, where response to treatment is defined as a ≥50% decrease in the self-report version of the Quick Inventory of Depressive Symptoms (QIDS-SR-16) total score from Baseline (V2) to Week 3 (V6). (See **Section 1.7.1** for more information about QIDS-SR-16).

b) The proportion of participants in remission, where remission is defined as a QIDS-SR-16 total score ≤5 at Week 3. This will also be assessed at Weeks 1 and 6.

3. To assess the participant-rated efficacy of psilocybin 25mg compared to placebo via:

a) The change in the Maudsley Visual Analogue Scale (MVAS) total score and individual items from Baseline to Day 1, Week 1, Week 3, and Week 6

4. To evaluate the safety and tolerability of psilocybin in participants with treatment resistant major depressive disorder (TRD) based on adverse events (AEs), changes in vital signs and suicidal ideation/behaviour (measured using the Columbia Suicide Severity Rating Scale).

In addition to the MADRS and QIDS-SR-16, there are several other clinical and safety measures of interest (See Section 1.7 for more details). Although these other measures were not specified explicitly in the protocol as secondary outcomes, preliminary estimates of effect sizes for these measures will be of clinical interest and will be reported by trial statisticians.

1.2. Trial design

PsiDeR is a two arm, double-blind, randomized, placebo controlled, between-subjects design in up to 60 completing participants meeting Diagnostic and Statistical Manual of Mental Disorders (5th Edition) (DSM-5) criteria for single or recurrent episodes of major depressive disorder (MDD) of at least moderate severity (but without psychotic features) who have failed to respond to EITHER 2 or more antidepressants prescribed at the minimum effective dose for at least 6 weeks OR at least 1 antidepressant prescribed at the minimum effective dose for at least 6 weeks AND a course of evidence-based psychological therapy given for at least 6 sessions. See Figure 1 for a representation of the overall trial structure. All study visits will take place at the NIHR/Wellcome Trust Clinical Research Facility at King's College Hospital, Denmark Hill, London, UK.

1.3. Eligibility criteria

1.3.1. Inclusion criteria:

- Age 25 - 80 years
- Fluent in the English language
- Fulfil Diagnostic and Statistical Manual of Mental Disorders (5th Edition) (DSM-5) criteria for a primary diagnosis of current single or recurrent episodes of MDD of at least moderate severity but without psychotic features as defined on the MINI 7.0 (Mini-International Neuropsychiatric Interview). Positive and primary diagnoses on the MINI 7.0 will be subject to confirmation at clinical interview by a psychiatrist.
- 17-item HAM-D (Hamilton Depression Rating Scale) score ≥ 14.

- Have failed to respond to 2 or more antidepressants prescribed at the minimum effective dose for at least 6 weeks OR at least 1 antidepressant prescribed at the minimum effective dose for at least 6 weeks AND a course of evidence-based psychotherapy given for at least 6 sessions.
- For those aged ≥ 60 years, the first episode of depression must have started prior to their 60th birthday.

1.3.2. Exclusion criteria

- Diagnosis of bipolar disorder (defined as meeting DSM-5 criteria for bipolar 1 or bipolar 2) on the MINI 7.0. Positive diagnoses on the MINI will be subject to confirmation at clinical interview by a psychiatrist.
- Diagnosis of psychotic disorder (defined as meeting DSM-5 criteria for any psychotic disorder) on the MINI 7.0, EXCEPT substance/medication induced psychotic disorder where the duration was limited to the acute period of direct intoxication with the substance/medication. Positive diagnoses on the MINI will be subject to confirmation at clinical interview by a psychiatrist.
- Diagnosis of drug or alcohol dependence syndrome (defined as meeting DSM-5 criteria for any dependence syndrome) on the MINI 7.0. Positive diagnoses on the MINI will be subject to confirmation at clinical interview by a psychiatrist.
- Diagnosis of any personality disorder (defined as meeting DSM-5 criteria for any personality disorder) based on clinical interview and the MINI 7.0. Positive diagnoses on the MINI will be subject to confirmation at clinical interview by a psychiatrist.
- Diagnosis of any dementia (defined as meeting DSM-5 criteria for any dementia disorder) based on clinical interview by a psychiatrist.
- Personal history of a ≥ 1 suicide attempt in the past year requiring hospitalization, defined using the CSSRS (Q6 (past year) = "y") and clinical interview with a psychiatrist.
- Other personal circumstances and behaviour judged to be incompatible with establishment of rapport or safe exposure to psilocybin.
- Depression secondary to other medical conditions.
- Medical diagnosis incompatible with psilocybin treatment (see Section 6.2.1 in protocol).
- Inability to provide a screening blood sample, urine sample or electrocardiogram.
- Biochemical abnormalities (defined as falling outside the normal reference range) as evaluated by a full blood count, full biochemistry profile and thyroid function tests. Biochemical abnormalities must also be determined as clinically significant by a medical doctor to fulfil the criterion for exclusion.

- Electrocardiographic abnormalities, defined as any abnormality that is not normal sinus rhythm and determined as clinically significant by a medical doctor.
- Women of child bearing potential not using adequate contraception (see Section 6.2.2 in protocol).
- Pregnant or breast-feeding women.
- Those unable to give informed consent.
- Non-registration with a GP or failure to consent to sharing of the GP summary care record and any psychiatric assessments held.
- Those enrolled in another drug trial.
- Hypersensitivity to the IMP or to any of the excipients or placebo

1.4. Randomisation and blinding

The randomisation service will be provided by the King's Clinical Trials Unit (KCTU). Randomisation will use the web-based service hosted at the KCTU in accordance with a standard operating procedure and held on a secure server. The allocation sequence will be generated using minimisation, with **sex** (male, female) **age group** (25 to 59, and 60 plus) and **past psilocybin use** (yes, no) as factors. The sequence will be concealed from all researchers and the trial statistician. A patient identification number from the web-based randomisation system will be provided after the patient has been both consented and randomised.

Allocation will be at the level of the individual participant, to the treatment and placebo arms (ratio 1:1) up to 60 completing participants allocated to two groups of 30.

The PI and trial assistant and researchers have a password to access the system and are authorised to randomise patients through the system. The system automatically generates emails to confirm allocation and sends randomisation information (blinded or unblinded) to the trial team as well as to Fisher Clinical Services, who labels and package the treatment according to trial regulations with participant identification details. This package will then be sent by secure transport to the Maudsley Hospital Pharmacy, who will store the treatment prior to administration.

The statisticians will be blind to the trial randomisation. The junior trial statistician may be partially unblinded after approval of the SAP in order to perform data cleaning tasks or to complete partially blinded closed reports for the data monitoring committee as needed. The senior trial statistician will remain blinded until review of the draft statistical report. The delegation log for the analysis is found in Appendix 2. The trial management team will remain blinded, and the MADRS is administered

by blinded assessors. The trial management team will remain blinded until review of the study results after database lock.

1.5. Start of treatment and duration of treatment period

The trial is expected to begin recruitment in January/February 2021 and finish recruitment in February 2024. The total duration of a participant's involvement in the trial will vary between 7 and 26 weeks, dependent on the length of the (clinically flexible) screening & preparation phase and whether they consent to the open label extension. (the analysis of the open label extension is beyond the scope of this SAP). The follow up of participants will last for 6 weeks after the Dosing Visit (V3) (see **Figure 1**). The open-label extension will last up to 12 weeks. Participants who undertake only the main trial maybe involved with the trial team for 14 weeks, and those who undertake both the main trial and open label extension may be involved with the study team for up to 26 weeks. Ongoing care for participants will be handed over to primary or secondary care teams after this time.

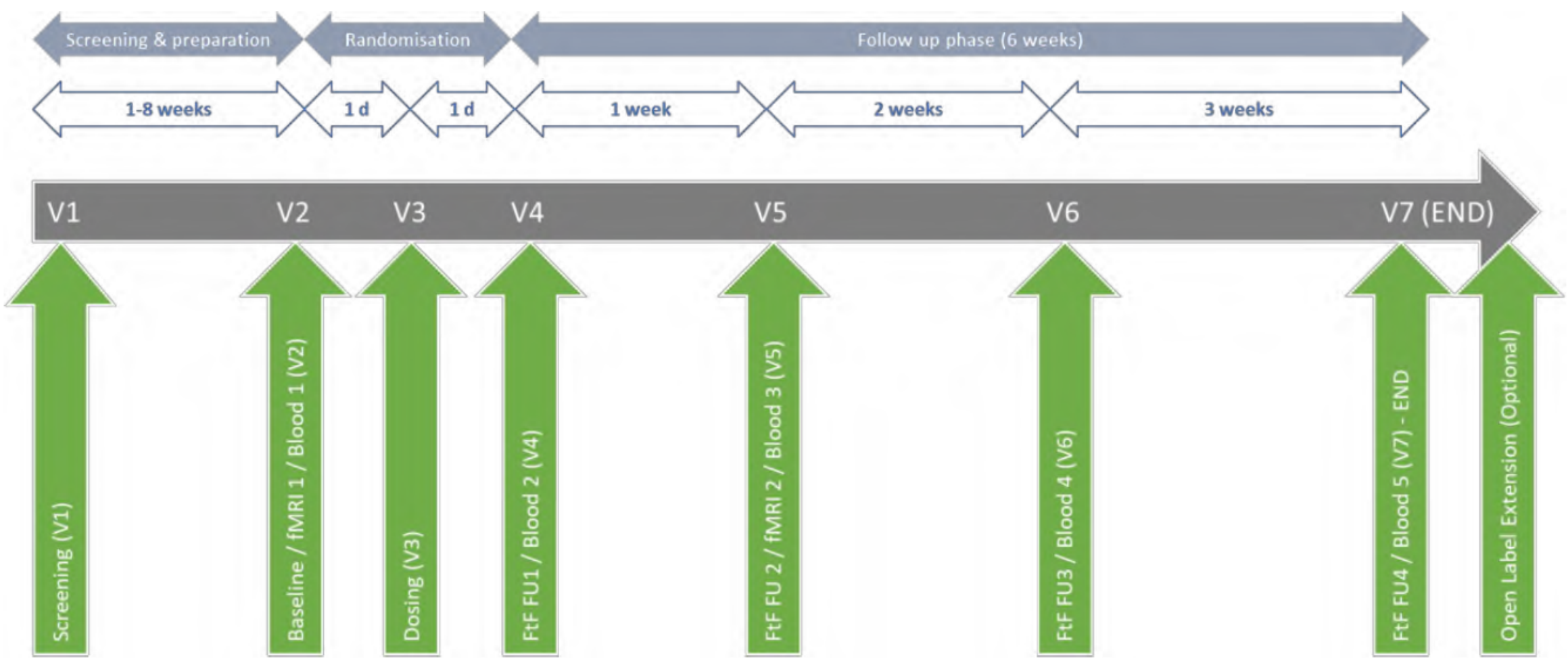

Figure. 1. Study timeline. Abbreviations: FtF: Face to Face, FU: Follow up, V: visit

1.6. Frequency and duration of follow-up

1.6.1. Screening & Preparation (SP) Phase (1-8 Weeks after consent)

Participants who are deemed eligible at the screening visit will be assessed by a study psychiatrist and enter a Screening & Preparation (SP) phase of between 1-8 weeks. The time is required for existing treatments to be withdrawn, to get collateral information from GPs and CMHTs, withdrawal from antidepressant, antipsychotic or mood stabilising treatments, and prepare the participant about the procedure of the study.

1.6.2. Baseline Visit and randomisation (V2 – Day 0)

Final eligibility will be confirmed at this visit, considering all the information available to the study team. At the Baseline Visit (V2), participants attend the CRF. Depending on consent, we will draw a blood sample for biological analysis (BioResource). We will perform baseline clinical procedures and

measures of outcome and functioning as described in the schedule of assessments. Depending on consent, we will ask participants to undergo a 1-hour fMRI scan. Participants will be randomised at V2.

1.6.3. Dosing Visit (Treatment) (V3 – 1 to 3 Days after baseline)

The Dosing Visit (V3) will take place 1, 2 or 3 days after the Baseline Visit (V2). Participants will receive a single dose of either 25mg of psilocybin, per os, or Placebo. Participants and the study team will be blind to treatment allocation.

1.6.4. Follow Up Visit 1 (V4 - 1 Day After Treatment)

The allowable window for this visit is +1 day. Consent depending, we will draw a blood sample. We will ask participants to complete self-report measures. A member of the study team will perform study team-rated measures.

1.6.5. Follow Up Visit 2 (V5 - 1 Week After Treatment)

The allowable window for this visit is +/-1 day. Consent depending, we will draw a blood sample. We will ask participants to complete self-report measures as described in the schedule of assessments. A member of the study team will perform study team-rated measures as described in the schedule of assessments (see **Table 1**). Participants will then be invited to undergo a 1-hour MRI scan at the Centre for Neuroimaging Sciences.

1.6.6. Follow Up Visit 3 (V6 - 3 Weeks After Treatment)

The allowable window for this visit is +/-2 days. Consent depending, we will draw a blood sample. We will ask participants to complete self-report measures as described in the schedule of assessments. A member of the study team will perform study team-rated measures as described in the schedule of assessments. (see **Table 1**)

1.6.7. Follow Up Visit 4 (V7 - 6 Weeks After Treatment)

The allowable window for this visit is +7 days and -3 days. Consent depending, we will draw a blood sample. We will ask participants to complete self-report measures as described in the schedule of assessments (see **Table 1**). A member of the study team will perform study team-rated measures. At this visit we will ask participants to share step counts and sleep data that has been collected by their personal smartphones or fitness watches, if they have them and have indicated their signed consent to this in the PIS.

1.6.8. Open-Label Extension

Participants who complete follow up visits to 6 weeks and who are deemed eligible by the study team will be offered a 12-week open label extension (OLE) to the trial. This will not a part of the feasibility assessment in the SAP.

1.7. Measures

Table 1. The variable list, with the primary function of the variable and collection points. This SAP will cover analysis for only the primary feasibility outcomes and some clinical/safety/mechanism outcomes (indicated by * in this table). The rest of the measures are outside the scope of this SAP.

		Time Points						
**Variable**	**Primary function**	**V1**	**V2**	**V3**	**V4**	**V5**	**V6**	**V7**
Screening numbers*	Screening	•						
Informed consent*	Screening	•						
Inclusion*	Screening	•						
Exclusion*	Screening	•						
Medical history	Screening	•	•					
Prior & Concomitant Medications	Safety	•	•					
Physical Examination	Safety	•	•	•	•	•	•	•
Fitness Watch/Mobile Activity Data	Outcome							•
Collateral History	Safety	•	•					
Adverse Events (UKU/HPPDS)*	Safety	•	•	•	•	•	•	•
Clinical laboratory investigations	Screening	•	•		•			
Electrocardiogram	Screening	•			•			
Vital signs (BP/HR/Temp)*	Safety	•	•	•	•			
Weight*	Safety	•	•					•
Urinalysis	Screening	•						
Urine Drug Screen	Screening	•						
Serum/Urine Pregnancy Test (females only)	Safety	•	•					
Psychological Support & SI	Support		•	•	•	•	•	•
Treatment	Process		•					
MINI	Baseline	•						
HAM-D-17*	Baseline	•	•					
Maudsley Staging Method*	Screening	•						
MADRS*	Outcome		•			•	•	•
CSSRS*	Safety	•	•	•	•	•	•	•
YMRS*	Safety		•		•	•	•	•
STAR-C	Mechanism		•			•	•	•
QIDS-SR-16*	Outcome	•	•			•	•	•
GAD-7*	Outcome		•			•	•	•
CTS	Mechanism		•					
STAI*	Mechanism		•			•	•	•
STAR-P	Mechanism		•			•	•	•
DESS	Safety		•					
5D-ASC*	Mechanism				•			
ASRM*	Safety		•			•	•	•
SCL-90	Safety		•			•		
DePAS*	Safety					•	•	•
EQ-5D-5L*	Outcome		•				•	•
WSAS*	Outcome	•	•				•	•

		Time Points						
**Variable**	**Primary function**	**V1**	**V2**	**V3**	**V4**	**V5**	**V6**	**V7**
TAS	Mechanism		●					●
PANAS*	Safety		●		●	●	●	●
SCSS	Mechanism		●		●			●
SCST	Mechanism		●				●	●
CES	Mechanism				●			
TSDI	Mechanism	●	●					●
WEMWBS*	Mechanism/Outcome		●				●	●
CNS	Mechanism		●				●	●
SAPAS	Mechanism	●	●					●
MTI	Screening	●						
THINC-it	Mechanism	●	●			●		●
ETB	Mechanism		●			●		●
MVAS*	Mechanism/Outcome	●	●		●	●	●	●

1.7.1. Clinical outcome measures of interest reported by trial statisticians

1. MADRS: Montgomery–Åsberg Depression Rating Scale, is a 10-item scale to measure the severity of depressive episodes in patients with mood disorders. Higher MADRS score indicates more severe depression, and each item yields a score of 0 to 6. The overall score ranges from 0 to 60.
2. QIDS-SR-16: The Quick Inventory of Depressive Symptomatology (16-Item) (Self-Report). Total score range 0-27. Severity of depression can be judged based on the total score. 1-5 = No depression, 6-10 = Mild depression, 11-15 = Moderate depression, 16-20 = Severe depression, 21-27 = Very severe depression.
3. GAD-7: Generalised Anxiety Disorder Assessment. GAD-7 has 7 items with likert response choices between 0 to 3. total score for the seven items ranges from 0 to 21. Scores of 5, 10, and 15 represent cut-points for mild, moderate, and severe anxiety, respectively. This measure was only listed as an exploratory endpoint/outcome in the protocol, but represents a clinical outcome of interest.
4. EQ-5D-5L: Euro-QOL Health Questionnaire. The questionnaire has 5 Likert items and 1 Visual Analogue Scale (VAS) question self-rating overall health on a scale of 0 (worst health imaginable today) to 100 (best health imaginable today). Only the VAS rating will be summarised by trial statisticians. This measure was not listed explicitly as a secondary outcome in the protocol, but represents a clinical outcome of interest as a self-rated measure of overall health.
5. WSAS: Work and Social Adjustment Scale. There are 5 items in the scale. The response for each item can be 0 (not at all) to 8 (very severely). The score has a range between 0 to 40. Lower scores are better. The total WSAS score is calculated by adding up all of the items. A WSAS score above 20 appears to suggest moderately severe or worse psychopathology. This

measure was only listed as an exploratory endpoint/outcome in the protocol, but represents a clinical outcome of interest.

6. MVAS: Maudsley Visual Analogue Scales. Questionnaire consisting of 3 visual analogue scales going from 0 to 100. Participants are asked to self-rate on the scale their quality of mood, experience of pleasure, and experience of suicidal thoughts or feelings. A total score is calculated by summing all items. Administered at all study visits.
7. WEMWBS: Warwick & Edinburgh Mental Wellbeing Scale: WEMWBS. Questionnaire consisting of 14 items on a scale of 1 (none of the time) to 5 (all of the time). A total score is calculated (ranging from 14 to 70) by summing all items, higher scores indicate more positive wellbeing. The WEMWBS was only listed as a mechanism outcome in the protocol but also represents a clinical outcome of interest.

1.7.2. Screening / baseline measures reported by trial statisticians:

1. Informed consent
2. Inclusion: the number of patients met inclusion out of screened number. The number of patients excluded by the reason for exclusion.
3. Exclusion: The number of patients excluded by the reason for exclusion
4. HAM-D-17: The Hamilton Rating Scale for Depression. Scoring is based on the 17-item scale and scores of 0–7 are considered as being normal, 8–16 suggest mild depression, 17–23 moderate depression and scores over 24 are indicative of severe depression [3]; the maximum score being 52 on the 17-point scale.
5. MSM: Maudsley Staging Method. This is a 15-point scale that measures treatment resistance, participants need to score at least 5/15 to be eligible for the trial. The MSM is only administered at screening.

1.7.3. Safety measures reported by trial statisticians:

1. Adverse Events (UKU/HPPDS)
2. Vital signs (BP/HR/Temp)
3. Weight in kg.
4. CSSRS: COLUMBIA-SUICIDE SEVERITY RATING SCALE. The C-SSRS is made up of ten categories, all of which maintain binary responses (yes/no) to indicate a presence or absence of the behaviour. The number and proportion of participants reporting suicidal ideation will be reported.
5. DePAS: Delayed Psychedelic After Effects Scale. An exploratory measure created for this trial, consists of 20 items assessing symptomatology across 10 domains using visual analogue scales.
6. YMRS: YOUNG MANIA RATING SCALE. The YMRS is an 11-item measure of the patient's clinical condition over the past 48 hours. 4 items (#5/irritability, #6/speech, #8/thought content, #9/disruptive/aggressive behaviour) are scored on a 0-8 scale and the remaining 7 items are

scored on a 0-4 scale. A total score ranging from 0 to 60 is calculated by summing the value of items, higher scores indicate more severe mania. This measure was listed as a 'safety' outcome in the protocol but also represents a clinical measure of interest.

7. ASRM: Altman Self-Rating Mania Scale. The ASRM is a 5-item self-reported measure of mania symptoms. Each item is scored on a scale of 0-4 and a total score (range: 0 to 20) is calculated, with higher scores indicating more severe mania. If 2 or more items are left unanswered, a total score is not calculated. If 1 item is missing, the raw total is multiplied by 5 then divided by 4 to get the prorated total score. This measure was listed as a 'safety' outcome in the protocol but also represents a clinical measure of interest.
8. PANAS: Positive and Negative Affect Scale. 20-item measure of positive and negative affect (10 items each on scale of 1-5). A summary score is calculated by finding the sum of items and totals range from 10 to 50, which higher scores indicating greater positive/negative affect. This measure was listed as a 'safety' outcome in the protocol but also represents a clinical measure of interest.

1.7.4. Mechanism measures covered by trial statisticians:

1. STAI: State-Trait Anxiety Inventory. The full 40-item version consists of two subscales of 20 items each measuring state and trait facets of anxiety. Items are score on a scale of 1 (not at all / almost never) to 4 (very much so / almost always) and total scores are the sum of the items. Higher scores indicate greater anxiety. The trait anxiety (Form Y-2) is used in this study, total scores range from 20 to 80.
2. 5D-ASC: 5 Dimension Altered States of Consciousness Questionnaire. A 94-item self-reported measure of the acute effects of psychoactive drugs. Only administered at Day 1, a total score will be calculated by summing all items.

1.7.5. Feasibility measures

1. Screening numbers: this encompasses the number of patients eligible and not eligible based by the reason, numbers not interested, not reachable, exclude due to safety concerns,
2. Adverse events and serious adverse events
3. Dropout rate during the preparation phase
4. Retention rate between randomisation and Week 6 and reasons for withdrawal

1.8. Sample size calculation

60 participants will be recruited in this study. The primary aim of the trial is to assess feasibility parameters for treatment and the trial (recruitment rates, loss to follow up) and estimate a variance parameter to inform a sample size calculation for a larger efficacy trial in the future. The expected rate of loss to follow-up is 20%. With 60 participants we would be able to estimate this target rate with a

95% confidence interval of ± 10.1% (9.9% to 30.1%). For the estimate of effect size, the modest sample sizes in pilot trials mean that this is sensitive to inflation (Sim & Lewis, 2012).

2. Quantitative Analysis Plan

2.1. Overview

This section will describe the plan for any quantitative statistical analysis for feasibility trial PsiDeR. According to the NIHR, a feasibility study focuses on conducting research to examine whether the study can be done [NIHR, 2012]. The purpose of this trial is to evaluate the feasibility, safety and explore how possible effectiveness of psilocybin by measuring the means, confidence intervals and effect sizes would look like, given under supportive conditions, in a randomised, blinded trial design in adult participants with treatment resistant major depressive disorder. No estimand has been defined, but the data from this feasibility trial will help inform the estimand for a larger future trial.

2.2. Description of the trial

PsiDeR is a two arm, double-blind, randomized, placebo controlled, between-subjects design in up to 60 completing participants meeting Diagnostic and Statistical Manual of Mental Disorders (5th Edition) (DSM-5) criteria for single or recurrent episodes of major depressive disorder (MDD) of at least moderate severity (but without psychotic features) who have failed to respond to EITHER 2 or more antidepressants prescribed at the minimum effective dose for at least 6 weeks OR at least 1 antidepressant prescribed at the minimum effective dose for at least 6 weeks AND a course of evidence-based psychological therapy given for at least 6 sessions. Thus, the aim of this research is to conduct feasibility, safety and estimate preliminary efficacy of psilocybin in participants with TRD.

A CONSORT diagram will be constructed to summarise recruitment of TRD patient to the study, as well as their follow-up and retention. A template in shown in **Figure 2**.

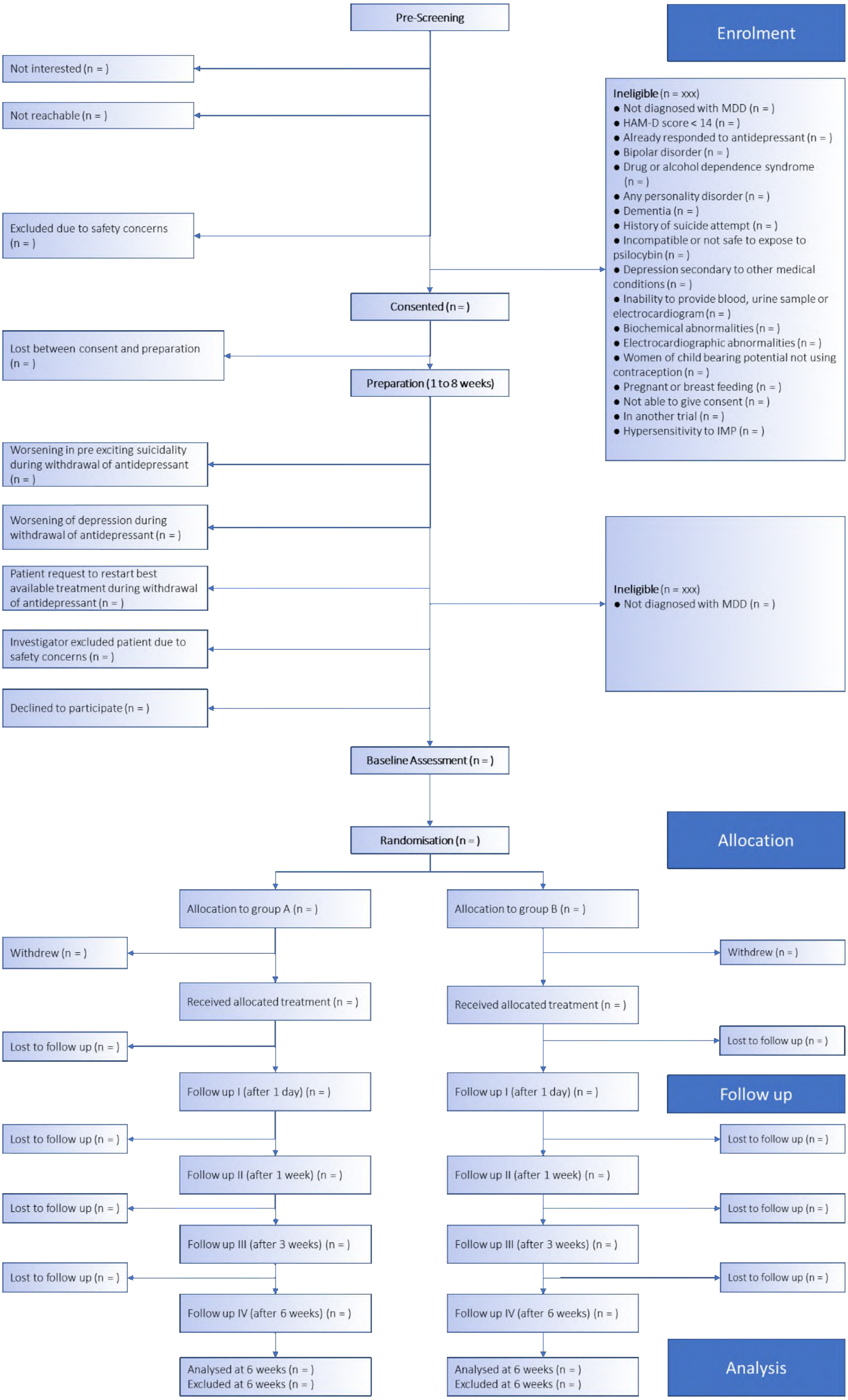

Figure 2. Template CONSORT diagram for PsiDeR feasibility trial

2.3. Objectives of the feasibility trial

This feasibility trial will estimate parameters needed for planning an efficacy trial including estimating various rates (e.g. recruitment, randomisation, follow-up rates) and estimating the within-trial arm standard deviation, the pre-post correlation and the Intra-cluster Correlation Coefficient (ICC) for possible outcome measures to inform the sample size calculation of a future randomised control trial. Clinical outcome, safety and mechanism measures will be summarised descriptively at each timepoint by trial arm and overall. Intervention effect sizes (e.g. mean differences for continuous outcome measures, odds ratios for proportion having defined remission/response) will be estimated for clinical outcomes of interest, but no formal hypotheses testing will be carried out.

2.4. Description of the trial sample

In order to describe the trial sample and check that the trial arms are balanced, baseline clinical variables indicated in **Table 1** will be summarised overall and by trial arms. No statistical testing will be done to assess for between-arm differences. Summary statistics will be calculated, specifically the mean and standard deviation (SD) for all normally distributed continuous measures and median and quartile ranges for continuous measures with skewed distributions. Discrete outcomes will be described using frequencies and proportions.

2.5. Evaluation of the recruitment and retention parameters

2.5.1. Evaluate the recruitment capability

For evaluating the recruitment capabilities we will report numbers of patients screened and found eligible of the targeted population, number of patients not found eligible, recruitment rate, refusal rates for participation and refusal before randomization, numbers not reachable, numbers excluded due to safety concerns, and numbers found eligible at V2 (final eligibility). The following proportions will be reported alongside the corresponding 95% CI:

- Participation rate will be computed as the number of patients enrolled divided by the number approached to participate.
- Eligibility rate will be computed as the number of patients eligible divided by approached (screened)
- Randomisation rate will be computed as the number of patients randomised divided by consented patients.
- We also assess the feasibility of eligibility criteria, by reporting the numbers excluded based on the reason for exclusion.
- We will report the numbers who will consent, and enters the preparation phase (1 to 8 weeks), the numbers of patients who drops out because of worsening of the condition for withdrawal of antidepressant or decline to continue or any other reasons in this phase.

2.5.2. Evaluate the trial procedure and follow-up parameters

Appropriate summaries of the feasibility measures listed below will be reported:

- Follow-up rate will be computed as the number of participants at each follow-up visit that have at least the MADRS completed divided by randomised participants. The appropriate 95% CI will also be reported.
- Completeness of outcome measures: For each outcome measure listed in **Table 1** the number of observations out of the number of participants at the respective timepoint will be described.
- Mean (SD) or Median (IQR) score of each outcome measure at baseline and each follow-up timepoint (Table 1). Mean or median will be determined based on how normally distributed the score is (determined via inspection of histograms or other visual means).

2.6. Evaluation of the experience and acceptability of the trial intervention

The following PsiDeR intervention details will be summarised overall and by trial arm (where appropriate) to have an evaluation of the experience of intervention:

- Rate of attendance to intervention session (Visit 3). This will be defined as the number attending the dosing visit divided by the number randomised.
- Rate of patients receiving the full dosage of intervention IMP in each arm. This will be defined as the number receiving all 5 capsule/tablets divided by the number randomised.
- Adverse events (See Section 2.8 for more details)

Proportions/rates will be reported alongside the corresponding 95% confidence interval (CI). Adverse events will be summarised descriptively by arm using counts of events and counts of unique reporters. The reasons for not attending the intervention session will also be tabulated for both arms.

2.7. Description of the clinical outcome measures

2.7.1. Overview

Clinical outcomes of interest listed in **Section 1.7.1** (MADRS, QIDS-SR-16, GAD-7, VAS from the EQ-5D-5L, WSAS, MVAS, WEMWBS) will be summarised by trial arm and trial arm differences (and standardised effect sizes) estimated for 1-week, 3-week and 6-week after randomisation (as well as 1-day after randomisation for MVAS). 95% confidence intervals will be constructed for the trial arm differences. Trial arm adjusted mean differences and their associated 95% CIs will be standardised by dividing by the (pooled-group) baseline SD of the outcome. We note that while we construct such inferences these are not to be used for significance testing; the trial is a small feasibility trial and we are not powered to detect a single pre-specified difference; never mind detecting differences on multiple outcomes. The purpose of our effect size estimates is purely to illustrate the size of effects across a

number of possible outcomes. Analysis will be based on the intention-to-treat principle. No significance testing will be performed and no p-values will be reported.

2.7.2. Preliminary estimations of patients' responses to intervention

We will estimate the adjusted mean difference (aMD) in the expected values of the continuous outcomes listed in **Section 1.7** using mixed linear regression models. These analyses will provide the treatment effect estimate on each outcome at 1, 3 and 6 weeks post randomisation (and 1 day post randomisation when applicable). The linear regression model will contain the post-treatment measures of the outcome as the dependent variables and a random intercept at the participant level to account for repeated measurements. Fixed effects will be fitted for the following predictors:

- Baseline measures of the outcome
- Trial arm
- Design factors (sex, age group, past psilocybin use)
- Time and an arm*time interaction term to allow estimates of the treatment effect at each follow up timepoint

Secondary binary outcomes (i.e. odds of achieving clinician-rated remission defined as MADRS total score ≤10 at follow up) will be analysed using mixed logistic regression models that include a random intercept at the participant level and adjust for the fixed effects of trial arm, baseline value (i.e. baseline total MADRS score), minimisation factors, time, and an arm*time interaction term to allow estimation of treatment effect at each follow up timepoint. Marginal odds ratios (ORs) and corresponding 95% CIs will be reported.

Although the GAD-7 at Week 3 was only specified in the protocol as an exploratory endpoint, we will also compare the odds of reporting moderate anxiety (GAD-7 ≥10) between arms at each follow up timepoint using the same method.

Time to restarting antidepressant medication was specified in the protocol as a secondary endpoint but not explicitly mentioned in the list of secondary objectives (although it may be related to secondary objective #4 in evaluating the safety and tolerability of psilocybin). Using information from the concomitant medication log, the time from the Dosing Visit to the date of first restarting antidepressant medication for any reason will be compared between arms. Participants that do not restart antidepressants before the end of the follow up period or who are withdrawn will be right-censored. Time to first restart of antidepressants will be predicted in Cox regression models by the fixed effects of trial arm and minimisation factors. A hazard ratio (HR) and corresponding 95% CI will be reported, and the proportion hazards assumption will be checked using visual means. If the proportional hazards assumption appears not to have been met, alternative approaches will be considered (i.e. including arm*time interaction term).

2.7.3. Evaluation of missing data

Missing baseline data

All efforts will be made to avoid missing baseline data (i.e. requiring completion of baseline data before randomisation), but if this occurs, missing values for baseline model covariates will be imputed using mean imputation as per the recommendations of White and Thompson [2005].

Missing items in scales and subscales

Where available we will use missing value guidance provided for scales. Where this is not available, we will prorate missing items only when there are no more than 20% missing items (i.e. for a ten item questionnaire, prorate only where one or two items are missing) by replacing the missing item values with the mean value of the complete items for each individual. See Appendix for further details. If after prorating there are still missing total questionnaire scores at baseline, these will be imputed as described in the paragraph just above.

Prediction of Missing outcome data

Where there are two or more outcome timepoints, missing post-randomisation values will be dealt with by fitting linear mixed models to all the available data using maximum likelihood methods. Such an approach provides valid inferences under the assumption that the missing data mechanism is ignorable or missing at random (MAR). The results of this trial will help inform potential considerations for capturing reasons for missingness in a future trial.

Details of how to deal with missingness for each measure is presented in **Appendix I**.

2.8. Safety and adverse events

2.8.1. Safety parameters and adverse events

Adverse events and serious adverse events will be tabulated overall and by arm as counts of events and counts of people who have had events. We will present the level of severity and relatedness of AE and SAEs overall and by arm.

2.8.2. Serious Adverse Events (SAE)

A serious adverse event (SAE) is an AE that is fatal or life-threatening, or results in hospitalization, prolongation of hospitalization, persistent or significant disability/incapacity, or a congenital anomaly/birth defect. A life-threatening AE is an AE that, in the view of the investigator, places the subject at immediate risk of death from the reaction as it occurred. Important medical events that may not result in death, be life-threatening, or require hospitalization may be considered SAEs when, based upon appropriate medical judgment, they may jeopardize the subject or subject and may require medical or surgical intervention to prevent one of the outcomes listed in this definition. All SAEs collected with an outcome of "Fatal" will be presented in a listing.

2.9. Other analyses

Safety and mechanism outcomes listed in Section 1.7 will be summarised descriptively (i.e. mean or median total scores for continuous outcomes).

No formal interim or subgroup analyses have been planned for this study.

No definitions of protocol deviation or violation have been defined.

2.10 Data handling and quality assurance

Trial data is recorded on a secure Excel spreadsheet managed by the Principal Investigator. During data checking, trial statisticians will request an extract and perform range and valid value checks on clinical outcome, safety, and mechanism data covered under this SAP. Data analysis will be conducted on Stata version 18.0 (or later), R version 4.1.1 (or later) may be used for data visualisation processes. The trial statisticians will follow King's Clinical Trial Unit Statistics SOPs.

3 References

National Institute for Health and Care Research. (2024). *Feasibility studies.* NIHR Evaluation, Trials and Studies Coordinating Centre.

Sim, J., & Lewis, M. (2012). The size of a pilot study for a clinical trial should be calculated in relation to considerations of precision and efficiency. *J Clin Epidemiol*, *65*(3), 301-308. https://doi.org/10.1016/j.jclinepi.2011.07.011

White, I.R., & Thompson, S.G. (2005). Adjusting for partially missing baseline measurements in randomized trials. *Statist. Med, 24*(7)*,* 993-1007. https://doi.org/10.1002/sim.1981

Secondary clinical outcomes

Supplementary table S1. Summary of secondary clinical outcomes at baseline and follow-up

	Baseline			Week 1			Week 3			Week 6		
**Clinical scale**	**Placebo**	**Psilocybin**	**Overall**	**Placebo**	**Psilocybin**	**Overall**	**Placebo**	**Psilocybin**	**Overall**	**Placebo**	**Psilocybin**	**Overall**
MADRS total												
Number of participants (N)	30	30	60	29	30	59	29	30	59	29	30	59
Mean (SD)	29·5 (7·3)	29·7 (4·8)	29·6 (6·1)	26·7 (8·3)	15·2 (9·0)	20·8 (10·4)	28·8 (7·5)	18·3 (12·3)	23·5 (11·5)	29·3 (7·9)	16·3 (11·1)	22·7 (11·6)
MADRS response (% of N)												
No, <50% decrease from baseline	··	··	··	28 (97%)	14 (47%)	42 (71%)	28 (97%)	17 (57%)	45 (76%)	28 (97%)	15 (50%)	43 (73%)
Yes, >=50% decrease from baseline	··	··	··	1 (3%)	16 (53%)	17 (29%)	1 (3%)	13 (43%)	14 (24%)	1 (3%)	15 (50%)	16 (27%)
MADRS remission (% of N)												
No, MADRS >10	··	··	··	27 (93%)	20 (67%)	47 (80%)	28 (97%)	18 (60%)	46 (78%)	28 (97%)	18 (60%)	46 (78%)
Yes, MADRS <=10	··	··	··	2 (7%)	10 (33%)	12 (20%)	1 (3%)	12 (40%)	13 (22%)	1 (3%)	12 (40%)	13 (22%)
QIDS-SR-16 total												
Number of participants (N)	30	30	60	29	30	59	28	30	58	28	30	58
Mean (SD)	15·6 (5·3)	16·1 (3·9)	15·8 (4·6)	13·7 (5·4)	8·1 (4·7)	10·8 (5·8)	14·2 (4·6)	9·0 (5·9)	11·5 (5·9)	14·6 (5·0)	9·2 (5·6)	11·8 (6·0)
QIDS-SR-16 response (% of N)												
No, <50% decrease from baseline	··	··	··	26 (90%)	15 (50%)	41 (69%)	28 (100%)	15 (50%)	43 (74%)	27 (96%)	17 (57%)	44 (76%)
Yes, >=50% decrease from baseline	··	··	··	3 (10%)	15 (50%)	18 (31%)	0 (0%)	15 (50%)	15 (26%)	1 (4%)	13 (43%)	14 (24%)
QIDS-SR-16 remission (% of N)												
No, QIDS-SR-16 >5	··	··	··	26 (90%)	20 (67%)	46 (78%)	27 (96%)	18 (60%)	45 (78%)	26 (93%)	20 (67%)	46 (79%)

Yes, QIDS-SR-16 <=5	··	··	··	3 (10%)	10 (33%)	13 (22%)	1 (4%)	12 (40%)	13 (22%)	2 (7%)	10 (33%)	12 (21%)
GAD-7 total												
Number of participants (N)	30	30	60	29	30	59	28	30	58	29	30	59
Mean (SD)	12·1 (5·2)	11·5 (4·6)	11·8 (4·8)	11·3 (5·3)	5·8 (4·2)	8·5 (5·5)	12·5 (4·7)	7·2 (5·6)	9·8 (5·8)	11·7 (5·6)	6·6 (4·9)	9·1 (5·8)
GAD-7 moderate anxiety (% of N)												
No (<10)	··	··	··	12 (41%)	24 (80%)	36 (61%)	7 (25%)	20 (67%)	27 (47%)	11 (38%)	25 (83%)	36 (61%)
Yes (>=10)	··	··	··	17 (59%)	6 (20%)	23 (39%)	21 (75%)	10 (33%)	31 (53%)	18 (62%)	5 (17%)	23 (39%)
EQ-5D-5L VAS												
Number of participants	28	29	57	··	··	··	27	28	55	26	29	55
Mean (SD)	46·7 (20·4)	49·2 (20·0)	47·9 (20·1)	··	··	··	45·7 (17·4)	61·3 (19·8)	53·6 (20·1)	48·9 (18·4)	61·4 (22·2)	55·5 (21·2)
WSAS total												
Number of participants	30	30	60	··	··	··	28	30	58	29	30	59
Mean (SD)	24·4 (7·4)	26·8 (5·3)	25·6 (6·5)	··	··	··	24·6 (7·0)	18·4 (10·2)	21·4 (9·3)	25·2 (7·4)	17·8 (10·3)	21·4 (9·7)
M3VAS total												
Number of participants	28	29	57	29	27	56	27	28	55	26	29	55
Mean (SD)	212·8 (41·3)	225·1 (35·8)	219·1 (38·8)	20·9 (47·5)	69·8 (48·3)	44·5 (53·5)	7·8 (44·7)	52·6 (65·7)	30·6 (60·3)	22·0 (42·6)	59·8 (49·7)	41·9 (49·8)
WEMWBS total												
Number of participants	28	28	56	··	··	··	28	30	58	29	30	59
Mean (SD)	31·2 (6·6)	28·1 (4·9)	29·7 (5·9)	··	··	··	28·6 (7·0)	37·6 (8·6)	33·2 (9·0)	30·9 (8·0)	38·9 (8·6)	34·9 (9·2)

MADRS: Montgomery-Asberg Depression Rating Scale, QIDS-SR-16: Quick Inventory of Depressive Symptomatology (16-Item Self Report), GAD-7: Generalised Anxiety Disorder assessment (7-item), EQ-5D-5L VAS: Visual Analogue Scale from Euro-QOL, WSAS: Work and Social Adjustment Scale, M3VAS: Maudsley Visual Analogue Scales, WEMWBS: Warwick & Edinburgh Mental Wellbeing Scale

Supplementary figure 1: Profile plots of raw depression severity scores

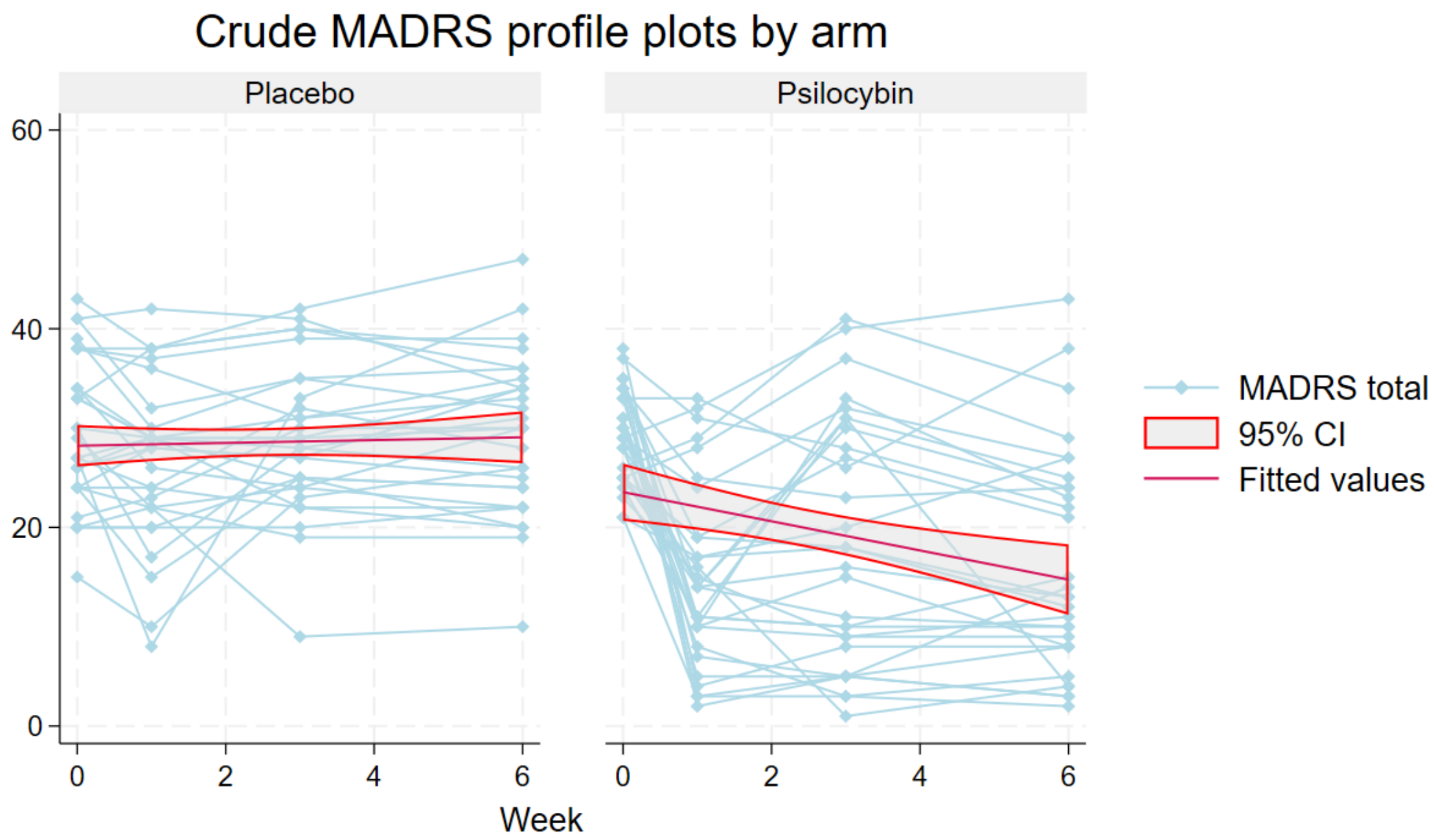

Placebo N=30, Psilocybin N=30. Higher score means more severe depression.

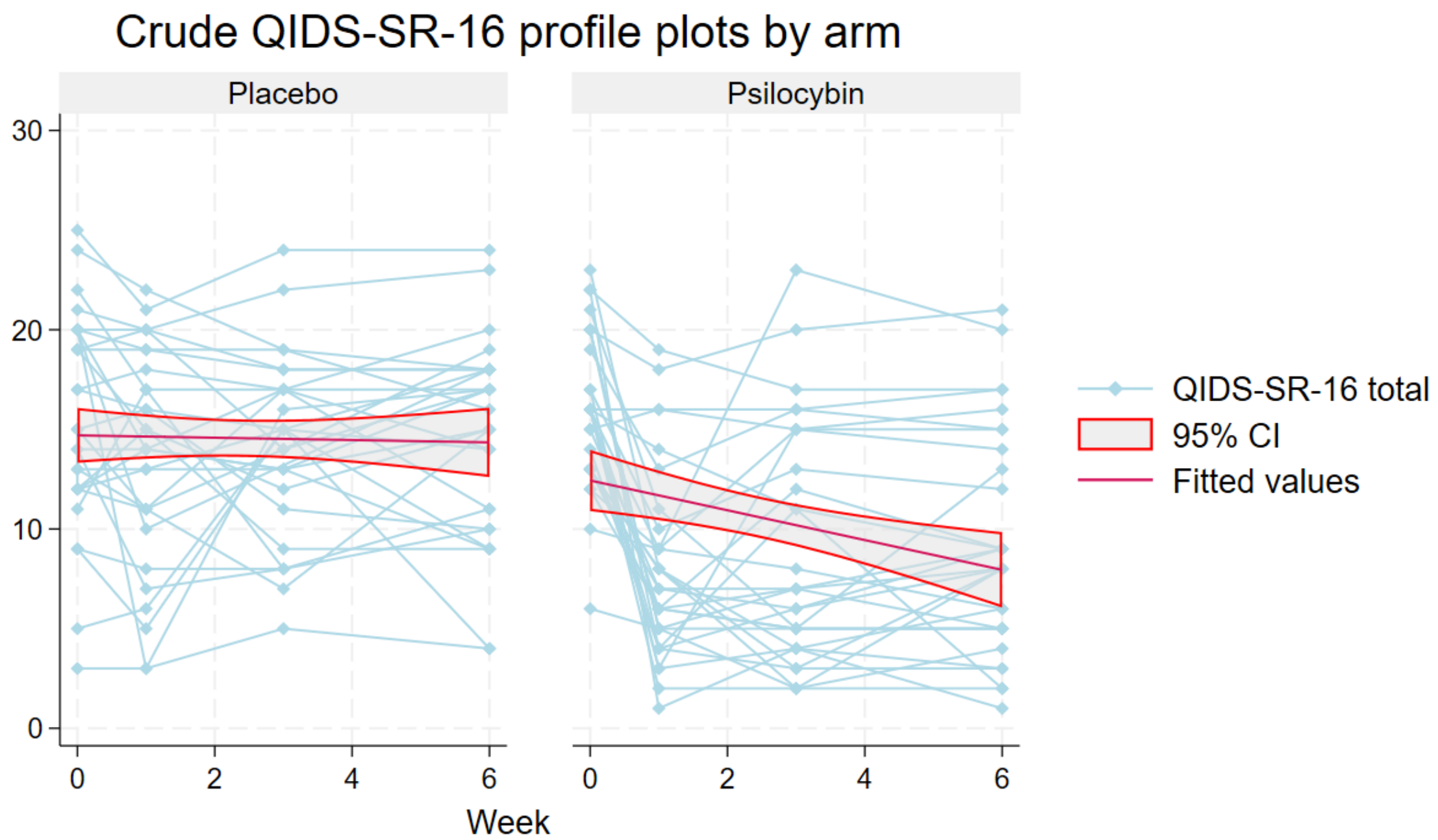

Placebo N=30, Psilocybin N=30. Higher score means more severe depression.

Supplementary figure 2. Predicted depression scores from mixed models.

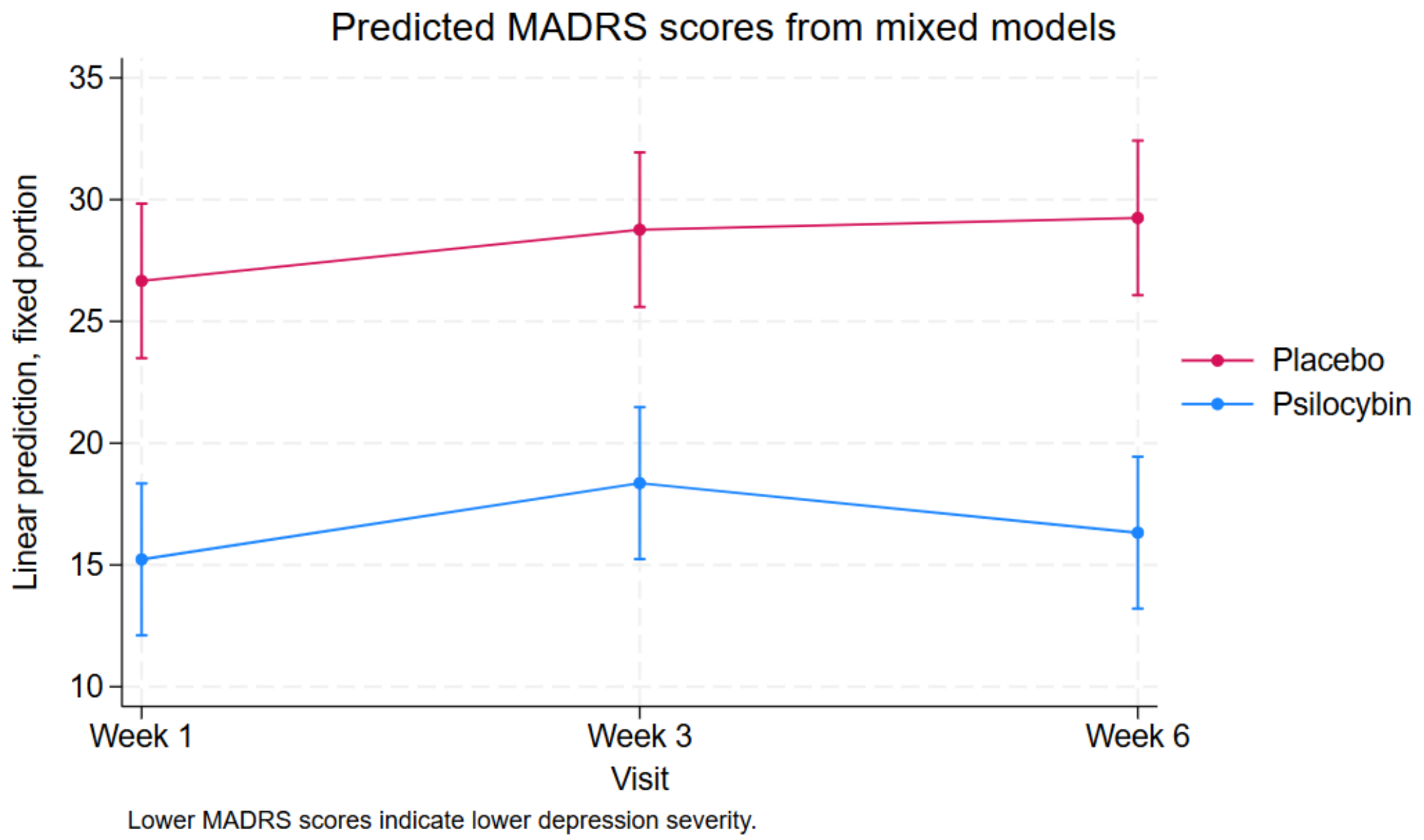

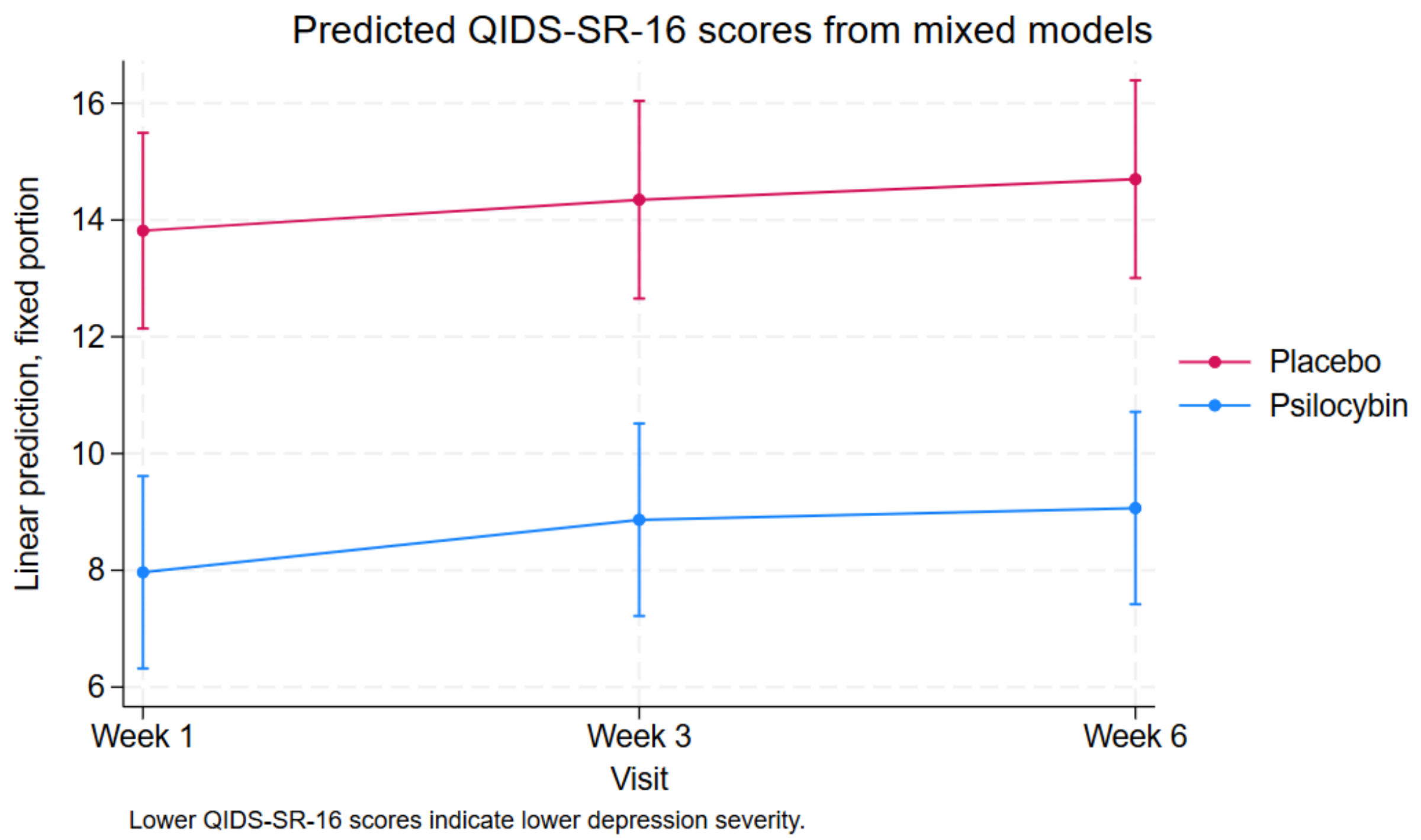

Supplementary table S2: Results of logistic regression analyses for secondary clinical binary outcomes

Outcome	Week	N in model	Conditional odds ratio (OR) [95% CI]	Marginal OR [95% CI]*
MADRS Response	Week 1	59	45.37 [4.69, 439.09]	18.74 [17.95, 19.79]
	Week 3	59	29.1 [3.2, 264.85]	13.42 [13.02, 13.83]
	Week 6	59	62.16 [5.99, 644.94]	18.46 [16.75, 19.83]
MADRS Remission	Week 1	59	8.67 [1.56, 48.1]	5.52 [5.43, 5.65]
	Week 3	59	24.1 [2.69, 216.07]	11.6 [11.26, 11.9]
	Week 6	59	33.94 [3.43, 335.77]	12.54 [11.82, 13.4]
QIDS-SR-16 Response	Week 1	59	11.69 [2.58, 53.06]	7.16 [6.94, 7.38]
	Week 3	N/A	Not estimable	Not estimable
	Week 6	58	29.45 [3.16, 274.58]	12.77 [12.43, 13.38]
QIDS-SR-16 Remission	Week 1	59	5.99 [1.27, 28.24]	4.19 [4.1, 4.33]
	Week 3	58	43.63 [3.34, 570.88]	13.46 [12.27, 15.4]
	Week 6	58	8.86 [1.52, 51.75]	5.49 [5.37, 5.68]
GAD-7 Moderate anxiety	Week 1	59	0.06 [0.01, 0.43]	0.25 [0.2, 0.3]
	Week 3	58	0.08 [0.02, 0.39]	0.18 [0.16, 0.21]
	Week 6	59	0.07 [0.01, 0.35]	0.19 [0.16, 0.21]

MADRS: Montgomery-Asberg Depression Rating Scale, QIDS-SR-16: Quick Inventory of Depressive Symptomatology (16-Item Self Report), GAD-7: Generalised Anxiety Disorder assessment (7-item).
*Marginal OR from Firth logistic regression models with intercept correction. 50 bootstrap samples used to generate percentile 95% CIs.
Conditional ORs are the ORs for psilocybin vs placebo at each timepoint for males with no past psilocybin use and with average age at randomisation and average baseline scores. For the QIDS-SR-16 response model, the Week 3 timepoint was not fit because there were no participants in the placebo arm at that time that had met the cut-off for self-rated depression response.

Serious Adverse Events

Supplementary table S3: Serious adverse event listing

MedDRA code	Medical description	Intensity	Outcome	Relatedness	Action	Start Date	End Date	AESI?	Arm
10083753	Localised infection	Severe	Resolving	Not related	Hospitalisation	03sep2022	07sep2022	Not AESI	Psilocybin
10024919	Depressed mood	Life threatening	Resolving	Possibly related	Other	07nov2023	.	Not AESI	Placebo
10006264	Breast mass	Moderate	Not resolved	Not related	None	10jun2024	.	Not AESI	Psilocybin
10072388	Drug induced psychosis	Severe	Unknown	Not related	Hospitalisation	04nov2024	.	Not AESI	Psilocybin

Further information: All events (except for breast mass event) were reported during the open-label phase. The drug induced psychosis developed in a patient treated with high doses of steroids for an unrelated physical health problem. Psychosis resolved once steroids were withdrawn.

Safety Scales

Supplementary table S4: Summary of safety scales

	**Baseline**			**Day 1**			**Week 1**			**Week 3**			**Week 6**		
**Safety scale**	**Placebo**	**Psilocybin**	**Overall**	**Placebo**	**Psilocybin**	**Overall**	**Placebo**	**Psilocybin**	**Overall**	**Placebo**	**Psilocybin**	**Overall**	**Placebo**	**Psilocybin**	**Overall**
CSSRS suicidal ideation															
No	27 (90%)	27 (90%)	54 (90%)	28 (93%)	30 (100%)	58 (97%)	27 (93%)	29 (97%)	56 (95%)	26 (90%)	27 (90%)	53 (90%)	25 (86%)	26 (87%)	51 (86%)
Yes	3 (10%)	3 (10%)	6 (10%)	2 (7%)	0 (0%)	2 (3%)	2 (7%)	1 (3%)	3 (5%)	3 (10%)	3 (10%)	6 (10%)	4 (14%)	4 (13%)	8 (14%)
Missing							*1 (3.3%)*	*0 (3.3%)*	*1 (1.7%)*	*1 (3.3%)*	*0 (3.3%)*	*1 (1.7%)*	*1 (3.3%)*	*0 (3.3%)*	*1 (1.7%)*
YMRS total, median (IQR)	1.0 (0-4.0)	0 (0-4.0)	0.5 (0-4.0)	0 (0-1.0)	0 (0-4.0)	0 (0-3.0)	1.0 (0-4.0)	1.0 (0-4.0)	1.0 (0-4.0)	0 (0-4.0)	0 (0-4.0)	0 (0-4.0)	0 (0-3.0)	1.0 (0-4.0)	0 (0-4.0)
Missing							*1 (3.3%)*	*0 (3.3%)*	*1 (1.7%)*	*1 (3.3%)*	*0 (3.3%)*	*1 (1.7%)*	*2 (6.7%)*	*0 (6.7%)*	*2 (3.3%)*
ASRM total, median (IQR)	0 (0-2.0)	0 (0-2.0)	0 (0-2.0)	--	--	--	1.0 (0-3.0)	3.5 (3.0-7.0)	3.0 (0-6.0)	0 (0-1.0)	3.5 (1.0-5.0)	1.0 (0-4.0)	1.0 (0-3.0)	3.0 (2.0-5.0)	2.0 (0.0-4.0)
Missing				--	--	--	*1 (3.3%)*	*0 (3.3%)*	*1 (1.7%)*	*2 (6.7%)*	*0 (6.7%)*	*2 (3.3%)*	*1 (3.3%)*	*0 (3.3%)*	*1 (1.7%)*
PANAS: Positive Affect, median (IQR)	19.0 (13.0-24.0)	16.0 (14.5-21.0)	18.0 (13.0-23.0)	18.5 (13.0-28.5)	28.5 (24.0-33.0)	25.0 (15.0-32.0)	16.0 (14.0-19.0)	24.5 (16.0-30)	19.0 (15.0-27.0)	16.0 (12.0-20.5)	20.5 (15.0-29.0)	17.5 (13.0-23.0)	18.0 (15.0-23.0)	26.0 (16.0-31.0)	23.0 (15.0-28.0)
Missing	*1 (3.3%)*	*2 (3.3%)*	*3 (5%)*	*2 (6.7%)*	*0 (6.7%)*	*2 (3.3%)*	*1 (3.3%)*	*0 (3.3%)*	*1 (1.7%)*	*2 (6.7%)*	*0 (6.7%)*	*2 (3.3%)*	*1 (3.3%)*	*0 (3.3%)*	*1 (1.7%)*

PANAS: Negative Affect, median (IQR)	18.0 (15.0-23.0)	18.5 (16.0-24.5)	18.0 (15.0-23.0)	17.5 (12.0-25.5)	14.5 (11.0-17.0)	15.0 (11.0-21.0)	18.0 (14.0-24.0)	14.0 (11.0-22.0)	16.0 (13.0-22.0)	23.0 (18.5-28.5)	16.5 (12.0-25.0)	20 (14.0-28.0)	20 (15.0-24.0)	15.0 (13.0-24.0)	16.0 (13.0-24.0)
Missing	*1 (3.3%)*	*2 (3.3%)*	*3 (5%)*	*2 (6.7%)*	*0 (6.7%)*	*2 (3.3%)*	*1 (3.3%)*	*0 (3.3%)*	*1 (1.7%)*	*2 (6.7%)*	*0 (6.7%)*	*2 (3.3%)*	*1 (3.3%)*	*0 (3.3%)*	*1 (1.7%)*
DEPAS total, median (IQR)	--	--	--	--	--	--	0 (-2.0-0)	3.0 (-7.0-10)	0 (-5.5-6.5)	0 (-2.0-0)	2.5 (-5.0-7.0)	0 (-4.0-5.0)	0 (-1.0-0)	2.0 (-3.0-6.0)	0.0 (-2.0-5.0)
Missing	--	--	--	--	--	--	*1 (3.3%)*	*3 (3.3%)*	*4 (6.7%)*	*3 (10%)*	*2 (10%)*	*5 (8.3%)*	*4 (13.3%)*	*1 (13.3%)*	*5 (8.3%)*

CSSRS: Columbia Suicide Severity Rating Scale, YMRS: Young Mania Rating Scale, ASRM: Altman Self-rating Mania Scale, PANAS: Positive and Negative Affect Scale, DePAS: Delayed Psychedelic Affect Effects Scale

Mechanism Scales

Supplementary table S5: Summary of mechanism scales

	**Baseline**			**Day 1**			**Week 1**			**Week 3**			**Week 6**		
**Mechanism scale**	**Placebo**	**Psilocybin**	**Overall**	**Placebo**	**Psilocybin**	**Overall**	**Placebo**	**Psilocybin**	**Overall**	**Placebo**	**Psilocybin**	**Overall**	**Placebo**	**Psilocybin**	**Overall**
STAI (Trait anxiety) - median (IQR)	65.0 (60.0-70.0)	64.0 (60.0-67.0)	64.0 (60.0-68.0)	--	--	--	65.0 (60.0-69.0)	47.0 (40.0-57.0)	59.0 (45.0-66.0)	66.0 (63.5-69.0)	48.5 (42.0-61.0)	62.0 (48.0-67.0)	67.0 (62.0-69.0)	52.5 (44.0-61.0)	62.0 (48.0-68.0)
Missing							*1 (3.3%)*	*0 (3.3%)*	*1 (1.7%)*	*2 (6.7%)*	*0 (6.7%)*	*2 (3.3%)*	*1 (3.3%)*	*0 (3.3%)*	*1 (1.7%)*
5D-ASC total (94 items) - median (IQR)	--	--	--	3.6 (0.8-7.8)	47.8 (33.9-55.1)	22.7 (3.7-47.8)	--	--	--	--	--	--	--	--	--
Missing				*2 (7%)*	*1 (10%)*	*3 (5%)*									
M3VAS mood quality - mean (sd)	77.0 (26.0)	76.4 (27.0)	76.7 (26.3)	22.8 (27.2)	23.8 (29.4)	23.3 (28.1)	24.4 (28.8)	31.3 (23.7)	27.8 (26.5)	22.6 (29.4)	24.7 (28.7)	23.7 (28.8)	28.5 (25.2)	29.6 (30.3)	29.1 (27.7)
Missing	*2 (6.7%)*	*1 (6.7%)*	*3 (5%)*	*3 (10%)*	*0 (10%)*	*3 (5%)*	*1 (3.3%)*	*3 (3.3%)*	*4 (6.7%)*	*3 (10%)*	*2 (10%)*	*5 (8.3%)*	*4 (13.3%)*	*1 (13.3%)*	*5 (8.3%)*
M3VAS pleasure - mean (sd)	69.4 (16.8)	74.0 (11.4)	71.7 (14.4)	0.5 (21.2)	19.7 (19.3)	10.6 (22.2)	-1.6 (18.3)	20.1 (23.1)	8.9 (23.3)	-8.4 (15.6)	12.5 (27.0)	2.2 (24.4)	-4.1 (14.8)	14.9 (19.6)	5.9 (19.8)
Missing	*2 (6.7%)*	*1 (6.7%)*	*3 (5%)*	*3 (10%)*	*0 (10%)*	*3 (5%)*	*1 (3.3%)*	*3 (3.3%)*	*4 (6.7%)*	*3 (10%)*	*2 (10%)*	*5 (8.3%)*	*4 (13.3%)*	*1 (13.3%)*	*5 (8.3%)*
M3VAS suicidality – mean (sd)	66.4 (19.6)	74.8 (17.5)	70.6 (18.9)	0.1 (16.3)	12.2 (16.0)	6.5 (17.1)	-2.0 (14.2)	18.3 (18.3)	7.8 (19.1)	-6.3 (15.0)	15.4 (22.1)	4.8 (21.7)	-2.4 (14.5)	15.2 (17.8)	6.9 (18.5)
Missing	*2 (6.7%)*	*1 (6.7%)*	*3 (5%)*	*3 (10%)*	*0 (10%)*	*3 (5%)*	*1 (3.3%)*	*3 (3.3%)*	*4 (6.7%)*	*3 (10%)*	*2 (10%)*	*5 (8.3%)*	*4 (13.3%)*	*1 (13.3%)*	*5 (8.3%)*

STAI: State-Trait Anxiety Inventory, 5D-ASC: 5 Dimension Altered States of Consciousness Questionnaire, M3VAS: Maudsley Visual Analogue Scales
Note: higher STAI and 5D-ASC scores indicate higher trait anxiety and greater acute effects, respectively. The baseline M3VAS scales ask participants how they have been feeling in the past two weeks on a scale of 0 to 100, with higher scores indicating more depression, less enjoyment, and more suicidality. Follow-up M3VAS assessments ask participants to indicate how much these domains have changed since they received the trial treatment on a scale of -50 to 50, with higher scores indicating improvement in mood, enjoyment, and suicidality.

Adverse Events During RCT and Open-Label Phase

Supplementary table S6: Summary of non-serious adverse events during RCT and open-label phase

	Placebo N events = 241	Psilocybin N events = 251	Overall N events = 492
	(reporters = 29)	(reporters = 30)	(reporters = 59)
**Body system category**			
Neurological	69 (26)	75 (29)	144 (55)
Mood/Psychological	26 (18)	51 (19)	77 (37)
Respiratory infection	41 (19)	24 (13)	65 (32)
Gastrointestinal	25 (16)	24 (15)	49 (31)
Musculoskeletal/pain	19 (14)	22 (15)	41 (29)
Reproductive	6 (5)	6 (4)	12 (9)
Skin/ocular	5 (5)	3 (2)	8 (7)
Medication or substance related	5 (5)	13 (10)	18 (15)
General fatigue/tiredness	10 (7)	13 (11)	23 (18)
Other	35 (17)	20 (14)	55 (31)
**Other event details**			
Relatedness - n (%)			
Not related	151 (63%)	111 (44%)	262 (53%)
Possibly related	50 (21%)	96 (38%)	146 (30%)
Related	40 (17%)	44 (18%)	84 (17%)
Intensity - n (%)			
Mild	158 (66%)	173 (69%)	331 (67%)
Moderate	78 (32%)	71 (28%)	149 (30%)
Severe	5 (2%)	7 (3%)	12 (2%)
Outcome - n (%)			
Resolved	216 (90%)	217 (86%)	433 (88%)
Resolving	13 (5%)	25 (10%)	38 (8%)
Not resolved	10 (4%)	9 (4%)	19 (4%)
Resolved with sequelae	1 (0%)	0 (0%)	1 (0%)
Unknown	1 (0%)	0 (0%)	1 (0%)
Action taken - n (%)			
None	138 (57%)	172 (69%)	310 (63%)
Medication required	95 (39%)	76 (30%)	171 (35%)
Other	8 (3%)	3 (1%)	11 (2%)
Adverse event of special interest (AESI) - n (%)			
Not AESI	239 (99%)	249 (99%)	488 (99%)
AESI	2 (1%)	2 (1%)	4 (1%)

For body system categories, the number of events of that category are reported first, followed by the number of unique reporters in brackets.

Supplementary figure 3: Adverse event categories by arm

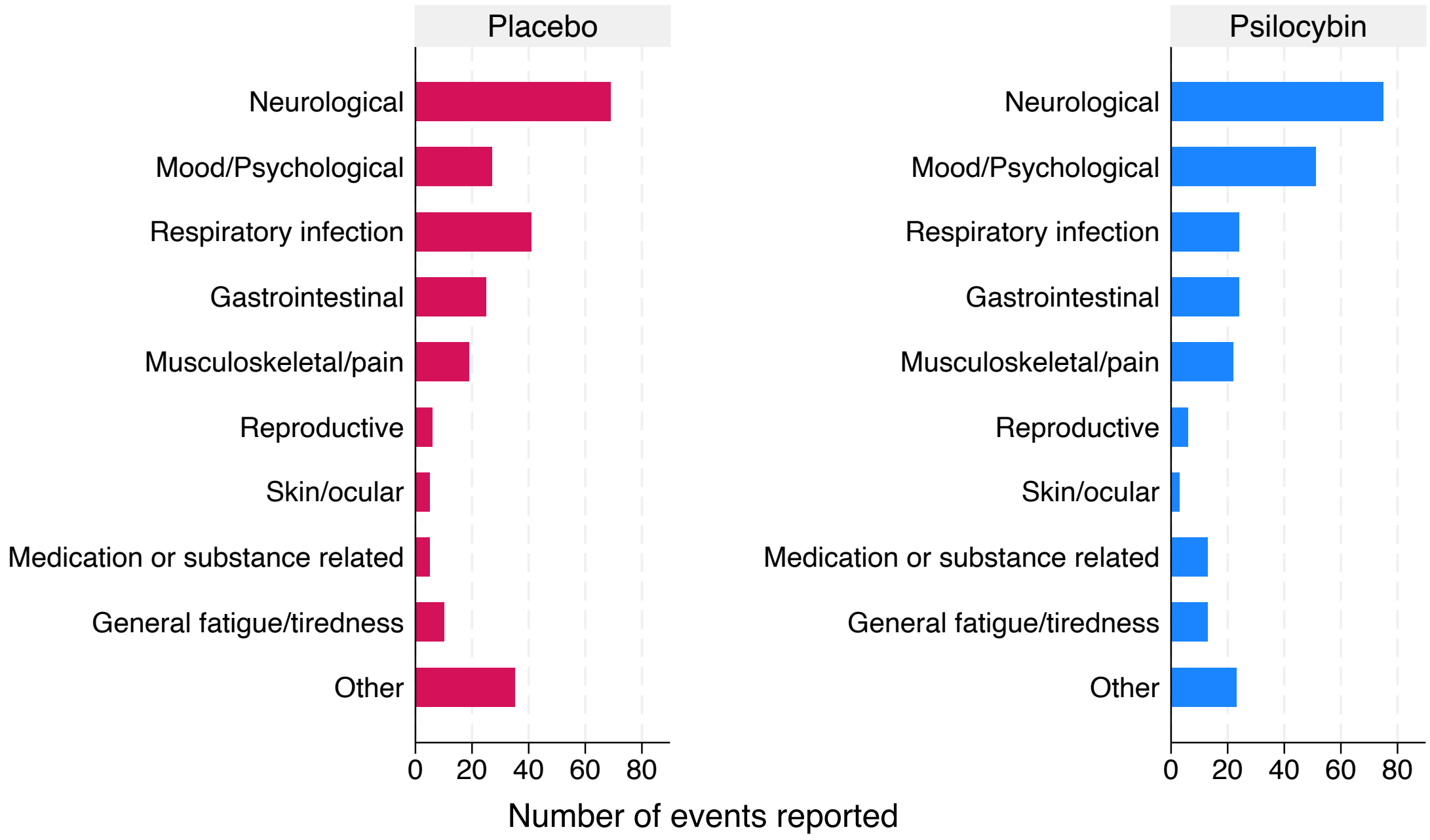

Adverse event categories by arm

Adverse events from the main and open-label extension are summarised here.

(Psi)locybin in (De)pression (R)esistant to Standard Treatments (PsiDeR) Trial Protocol

VERSION CONTROL

Document	Vers	Date	Author	Approval	Summary of Changes
Protocol	0.1	28-MAY-2017	James Rucker	Allan Young	Initial NIHR application
Protocol	0.2	14-MAR-2018	James Rucker	Allan Young	Initial draft. Incorporates feedback from service users and carers, supervisors, colleagues, the research design service, the NIHR and peer reviewers.
Protocol	0.3	09-MAY-2018	James Rucker	Allan Young	Updated in line with colleague and PPI feedback
Protocol	0.4	14-MAY-2018	James Rucker	Allan Young	Updated in line with statistician feedback.
Protocol	0.5	29-MAY-2018	James Rucker	Allan Young	Updated with details of open label extension. Cognitive and emotional test batteries added. For PPI feedback. Internal CAG approval obtained.
Protocol	0.6	07-JUN-2018	James Rucker	Allan Young<br>Service users	Updated after PPI feedback. Change to placebo control given open label extension.
Protocol	0.7	17-JUL-2018	James Rucker	Allan Young	Updated after TSC feedback.
Protocol	0.8	08-AUG-2018	James Rucker	Allan Young	Updated after CTO/CRF feedback
Protocol	0.85	19-SEP-2018	James Rucker	Allan Young	Further updates
Protocol	0.9	07-OCT-2018	James Rucker	Allan Young	IRAS submission version 1st draft
Protocol	0.91	13-NOV-2018	James Rucker	Allan Young	Updated with API changes, website changes.
Protocol	0.95	07-JAN-2019	James Rucker	Allan Young	BioResource updates.
Protocol	0.96	30-JAN-2019	James Rucker	Allan Young	Minor changes
Protocol	0.97	10-MAR-2019	James Rucker	Allan Young	Change to follow up protocol after TSC feedback. OLE addition.
Protocol	0.98	25-MAY-2019	James Rucker	Allan Young	Changes re: IMP supply agreement.
Protocol	0.99	25-JUL-2019	James Rucker	Allan Young	Minor changes
Protocol	1.00	02-JAN-2020	James Rucker	Allan Young	IRAS Submission
Protocol	1.10	11-MAR-2020	James Rucker	Allan Young	Updated in line with REC feedback
Protocol	1.12	12-JUN-2020	James Rucker	Allan Young	Minor amendments. MHRA submission
Protocol	1.13	09-AUG-2020	James Rucker	Allan Young	Updated in line with MHRA feedback
Protocol	1.14	06-JAN-2021	James Rucker	Allan Young	Changes to blinding procedure<br>Changes to code break procedure<br>Changes to compassion experience scale
Protocol	1.15	19-JAN-2021	James Rucker	Allan Young	Removal of BioResource. Updates to sample collection procedures.
Protocol	1.16	28-SEP-2021	James Rucker	Allan Young	Updates to open label extension window
Protocol	1.17	05-MAY-2022	James Rucker	Allan Young	Updates to blood collection procedures
Protocol	1.18	24-NOV-2022	James Rucker	Allan Young	Updates to secondary outcomes, blinding and expectancy questions, study end date Corrections to section 7.1.1. in line with 7.1.2
Protocol	1.19	15-SEP-2023	Tim Mantingh	Allan Young	Updates location of visits
Protocol	1.20	08-FEB-2024	Tim Mantingh	Allan Young	Updates to study end date and safety data reporting details
Protocol	1.21	15-AUG-2024	Tim Mantingh	Allan Young	Sponsor contact details updated

Protocol Full Title

A randomised, placebo-controlled trial of psilocybin in treatment resistant depression: a feasibility study

Protocol Short Title/Acronym

(Psi)locybin in (De)pression (R)esistant to standard treatment (PsiDeR)

Trial Identifiers

EudraCT Number	2018-003573-97
REC Number	20-LO/0206
IRAS Project ID	252750

Sponsor/Co-Sponsor:

King's College London/South London & Maudsley NHS Foundation Trust

Contact: Ann Marie Murtagh, King's Health Partners Clinical Trials Office

Address: F16 Guy's Tower, Guy's Hospital, Great Maze Pond, London, SE1 9RT

Telephone: +44 020 71885732

Email: QM.KHPCTO@kcl.ac.uk

Chief Investigator

Name: Professor Allan Young

Address: PO72. The Centre for Affective Disorders, The Institute of Psychiatry, Psychology & Neuroscience, 16 De Crespigny Park, London, SE5 8AF

Telephone: 02078480088

Fax: 02078480298

Email: allan.young@kcl.ac.uk

Co-Investigator(s)

Title: Principal Investigator

Name: Dr James Rucker

Address: PO72, The Centre for Affective Disorders, The Institute of Psychiatry, Psychology & Neuroscience, King’s College London,16 De Crespigny Park, London, SE5 8AF, United Kingdom

Telephone: 02078480088

Fax: 02078480298

Email: james.rucker@kcl.ac.uk

Title: Statistician

Name: Dr Ben Carter

Address: S2.04, Department of Biostatistics and Health Informatics, The Institute of Psychiatry, Psychology & Neuroscience, King's College London, 16 De Crespigny Park, London SE5 8AF, United Kingdom

Telephone: 02078480305

Fax: 02078480298

Email: ben.carter@kcl.ac.uk

Table of Contents

PROTOCOL FULL TITLE **38**

PROTOCOL SHORT TITLE/ACRONYM **38**

TRIAL IDENTIFIERS **38**

SPONSOR/CO-SPONSOR: **38**

CHIEF INVESTIGATOR **38**

CO-INVESTIGATOR(S) **38**

1. STUDY SYNOPSIS **44**

2. GLOSSARY OF TERMS **46**

3. BACKGROUND & RATIONALE **50**

3.1. NEED 50
3.2. BACKGROUND & RATIONALE 50
3.2.1. Pilot Work *51*
3.3. TRIAL DESCRIPTION 52
3.3.1. Trial Design *53*
3.3.2. Recruitment *53*
3.3.3. Screening *53*
3.3.4. Trial Flowchart & Structure *56*
3.3.5. Screening & Preparation (SP) Phase (1-8 weeks) *58*
3.3.6. Baseline Visit (V2) *60*
3.3.7. Dosing Visit (Treatment) (V3) *60*
3.3.8. Follow Up Phase (6 weeks) *62*
3.3.9. Follow Up Visit 1 (V4). 1 Day After Treatment. *62*
3.3.10. Follow Up Visit 2 (V5). 1 Week After Treatment. *63*
3.3.11. Follow Up Visit 3 (V6). 3 Weeks After Treatment. *63*
3.3.12. Follow Up Visit 4 (V7). 6 Weeks After Treatment. *63*
3.3.13. Open-Label Extension. *64*
3.3.14. Transfer of Care Arrangements *65*
3.3.15. Escape Criteria and Trial Withdrawal *65*

4. TRIAL OBJECTIVES AND DESIGN **66**

4.1. TRIAL PURPOSE 66
4.2. TRIAL OBJECTIVES 66

4.2.1. Primary Objective ... *66*
4.2.2. Secondary Objectives ... *66*
4.2.3. Exploratory Objectives ... *67*
4.3. TRIAL ENDPOINTS ... 67
4.3.1. Primary Endpoint ... *67*
4.3.2. Secondary Endpoints ... *67*
4.3.3. Exploratory Endpoints ... *67*
4.4. TRIAL DESIGN ... 67
4.5. TRIAL FLOWCHART ... 67

5. TRIAL MEDICATION ... 69

5.1. INVESTIGATIONAL MEDICINAL PRODUCT (IMP) ... 69
5.1.1. Summary of IMP Basic Chemical Properties ... *69*
5.1.2. IMP Manufacture ... *69*
5.1.3. IMP Stability & Shelf Life ... *70*
5.2. DOSING REGIMEN ... 70
5.3. IMP RISKS ... 70
5.3.1. Pharmacology ... *70*
5.3.2. Reported Experiences with Psilocybin ... *71*
5.3.3. Reported-Observed Findings in the Literature Coded Using MedDRA ... *71*
5.3.4. Psychological and Subjective Effects ... *72*
5.3.5. Safety Data Collection ... *74*
5.3.6. Acute Adverse Events ... *76*
5.3.7. Risky or Reckless Behaviour ... *76*
5.3.8. Delayed Adverse Events ... *77*
5.3.9. Prolonged Psychotic Reactions ... *78*
5.3.10. Hallucinogen Persisting Perception Disorder (HPPD) ... *78*
5.3.11. Chronic Neurotoxicity & Personality Change ... *79*
5.3.12. Abuse & Dependence Liability ... *80*
5.3.13. Damage to Non-Brain Organs ... *80*
5.3.14. Fatalities ... *81*
5.3.15. Serotonin Syndrome ... *82*
5.3.16. Legal Position ... *82*
5.4. DRUG ACCOUNTABILITY ... 83
5.5. STORAGE OF IMP ... 83
5.6. SUBJECT COMPLIANCE. ... 83
5.7. CONCOMITANT MEDICATION ... 83

5.8. End of Trial ... 84

6. SELECTION OF SUBJECTS ... 85

6.1. Inclusion Criteria ... 85

6.2. Exclusion Criteria ... 85

6.2.1. Exclusions for Pre-Existing Medical Conditions ... 86

6.2.2. Women of Child Bearing Potential ... 86

6.3. Recruitment of Participants ... 87

6.4. Randomisation Procedure ... 87

6.4.1. Implementation ... 88

6.5. Emergency Code Break ... 88

6.6. Withdrawal of Subjects ... 88

6.7. Expected Duration of Trial ... 88

7. TRIAL PROCEDURES ... 90

7.1. Trial Measures & Procedures ... 90

7.1.1. List of Measures & Procedures ... 90

7.1.2. Schedule of Measures & Procedures ... 92

7.1.3. Allowable Windows ... 93

7.1.4. Description of Study Team Administered Measures ... 93

7.1.5. Description of Participant Administered Measures ... 98

7.2. Open Label Extension (OLE) ... 103

7.3. Protocol Deviations ... 104

8. ASSESSMENT OF EFFICACY ... 105

8.1. Primary Efficacy Parameters ... 105

8.2. Secondary Efficacy Parameters ... 105

9. ASSESSMENT OF SAFETY ... 106

9.1. Specification, Timing and Recording of Safety Parameters. ... 106

9.2. Procedures for Recording and Reporting Adverse Events ... 107

9.2.1. Reporting Responsibilities ... 108

9.2.2. Documentation, Classification & Follow Up of Adverse Events ... 108

9.2.3. Other Procedures for Reporting and Recording Adverse Events ... 110

9.3. Adverse Events of Special Interest (AESI) ... 110

9.4. Adverse Events That Do Not Require Reporting ... 111

9.5. Treatment Stopping Rules ... 111

10. STATISTICS ... 112

10.1. SAMPLE SIZE ... 112
10.2. RANDOMISATION ... 112
10.3. ANALYSIS ... 112
10.4. TRIAL AND INTERVENTION FEASIBILITY PARAMETERS ... 112
10.5. ESTIMATES FOR EFFECT SIZES FOR PSILOCYBIN 25MG VS. PLACEBO. ... 113

11. TRIAL STEERING COMMITTEE ... 114

12. DATA MONITORING COMMITTEE ... 115

13. DIRECT ACCESS TO SOURCE DATA AND DOCUMENTS ... 116

14. ETHICS & REGULATORY APPROVALS ... 117

15. QUALITY ASSURANCE ... 118

16. DATA HANDLING ... 119

17. DATA MANAGEMENT ... 120

18. PUBLICATION POLICY ... 121

19. INSURANCE / INDEMNITY ... 122

20. FINANCIAL ASPECTS ... 123

21. SIGNATURES ... 124

REFERENCES ... 125

163 # Study Synopsis

Title of clinical trial	A randomised, placebo controlled trial of psilocybin in treatment resistant depression
Protocol Short Title/Acronym	(Psi)locybin in (De)pression (R)esistant to treatment (PsiDeR) trial
Study Phase, if not mentioned in title	Phase 2
Sponsor name	King's College London<br>South London & Maudsley NHS Foundation Trust
Chief Investigator	Professor Allan Young
EudraCT number	2018-003573-97
REC number	20-LO/0206
Medical condition or disease under investigation	Major depressive disorder (MDD)
Investigational Medicinal Product (IMP)	Psilocybin (3-[2-(Dimethylamino)ethyl]-1*H*-indol-4-yl dihydrogen phosphate)
Purpose of clinical trial	To evaluate the feasibility, safety and efficacy of psilocybin, given under supportive conditions, in a randomised, blinded trial design in adult participants with treatment resistant major depressive disorder
Primary objective	To evaluate the feasibility of a randomised, controlled trial design, in which a single dose of psilocybin 25mg PO vs placebo, is given to adult participants with treatment resistant major depressive disorder (TRD), under psychologically supportive conditions, with 6 weeks of follow up, by measuring recruitment rates, dropout rates and by estimating the variance of the primary outcome measure (MADRS) to inform upon the design of a phase 3 trial.
Secondary objective(s)	1) To assess the clinician-rated efficacy of psilocybin 25mg compared to placebo via:<br>a) The change in the Montgomery Asberg Depression Rating Scale (MADRS) total score from Baseline to 3 weeks after treatment.<br>b) The proportion of participants who demonstrate a response to treatment, where response to treatment is defined as a ≥50% decrease in MADRS total score from Baseline to Week 3. This will also be assessed at Weeks 1 and 6.<br>c) The proportion of participants in remission, where remission is defined as a MADRS total score ≤10 at Week 3. This will also be assessed at Weeks 1 and 6.<br>2) To assess the participant-rated efficacy of psilocybin 25mg compared to placebo via:<br>a) The proportion of participants who demonstrate a response to treatment, where response to treatment is defined as a ≥50% decrease in QIDS-SR-16 total score from Baseline (V2) to Week 3 (V6).<br>b) The proportion of participants in remission, where remission is defined as a QIDS-SR-16 total score ≤5 at Week 3. This will also be assessed at Weeks 1 and 6.<br>3) To evaluate the safety and tolerability of psilocybin in participants with TRD based on adverse events (AEs),

	changes in vital signs and suicidal ideation/behaviour (measured using the Columbia Suicide Severity Rating Scale).
Trial Design	Two arm, double-blind, randomised, placebo controlled, between-subjects design
Endpoints	Recruitment rates, dropout rates and estimation of the variance of the primary outcome measure (MADRS).
Sample Size	60
Summary of eligibility criteria	1) Age 25 - 80 years<br>2) Fluent in the English language<br>3) Fulfil Diagnostic and Statistical Manual of Mental Disorders (5th Edition) (DSM-5) criteria for current single or recurrent episodes of MDD of at least moderate severity but without psychotic features, as defined on the MINI 7.0.<br>4) 17-item HAM-D score ≥ 14.<br>5) Have failed to respond to 2 or more antidepressants prescribed at the minimum effective dose for at least 6 weeks OR at least 1 antidepressant prescribed at the minimum effective dose for at least 6 weeks AND a course of evidence-based psychotherapy given for at least 6 sessions.<br>6) For those aged ≥ 60 years, the first episode of depression must have started prior to their 60th birthday.
IMP, dosage and route of administration	Psilocybin 25mg per os
Comparator product(s)	Placebo
Maximum duration of treatment of a subject	1 day
Follow up Duration	6 (+12 OLE) weeks. Total of up to 18 weeks if eligible and consenting to OLE. Otherwise 6 weeks.
Planned Trial Period	September 2020 – February 2025
Funding	National Institute for Health Research (NIHR) CS-2017-17-007

164

Glossary of Terms

1H-MRS	Proton Magnetic Resonance Spectroscopy
5-HT	5-Hydroxy Tryptamine
5D-ASC	5-Dimension Altered State of Consciousness
AE	Adverse Event
AESI	Adverse Event of Special Interest
ANCOVA	Analysis of Co-Variance
AR	Adverse Reaction
ASRM	Altman Self-Rating Mania Scale
BNF	British National Formulary
BOLD	Blood Oxygen Level Dependent
BP	Blood Pressure
BRC	Biomedical Research Centre
CAG	Clinical Academic Group
CCSRS	Columbia Suicide Severity Rating Scale
CMHRI	Centre for Mental Health Research and Innovation
CMHT	Community Mental Health Team
CNS	Central Nervous System
CONSORT	Consolidated Standards of Reporting Trials
CRF	Clinical Research Facility
CRF	Case Report Form
CRN	Clinical Research Network
CRP	C Reactive Protein
CSSRS	Columbia Suicide Severity Rating Scale
CTO	Clinical Trials Office
DePAS	Delayed Psychedelic After Effects Scale
DESS	Discontinuation Emergent Signs and Symptoms
DMEC	Data Monitoring & Ethics Committee

DSM-5	Diagnostic and Statistical Manual of Mental Disorders (5th Edition)
DSST	Digit Symbol Substitution Test
DSUR	Development Safety Update Report
ECG	Electrocardiogram
EQ-5D-5L	EuroQoL-5D-5L Scale
FtF	Face to Face
FU	Follow Up
GABA	Gamma Amino Butyric Acid
GAD-7	Generalised Anxiety Disorder Scale
GCP	Good Clinical Practice
GI	Gastrointestinal
GP	General Practitioner
HAM-D	Hamilton Depression Rating Scale
HDPE	High Density Polyethylene
HPMC	Hydroxy Propyl Methyl Cellulose
HPPD	Hallucinogen Persisting Perception Disorder
HR	Heart Rate
IAPT	Improving Access to Psychological Therapies
IB	Investigator's Brochure
IL	Interleukin
IM	Intramuscular
IMP	Investigational Medicinal Product
ITT	Intention to Treat
IUPAC	International Union of Pure and Applied Chemistry
IV	Intravenous
KCL	King's College London
KCTU	King's Clinical Trials Unit

KG	Kilogram
KHP	King's Health Partners
LD-50	Lethal Dose, 50%
LSD	d-Lysergic Acid Diethylamide
MADRS	Montgomery Asberg Depression Rating Scale
MAR	Missing at Random
MDD	Major Depressive Disorder
MedDRA	Medical Dictionary for Regulatory Activities
MG	Milligram
MHRA	Medicines and Healthcare products Regulatory Agency
MINI	Mini Neuropsychiatric Inventory
ML	Millilitre
ML	Maximum Likelihood
MRI	Magnetic Resonance Imaging
MRS	Magnetic Resonance Spectroscopy
MSM	Maudsley Staging Method
MTI	Maudsley Treatment Inventory
MVAS	Maudsley Visual Analogue Scales
NEO FFI	NEO Five Factor Personality Inventory
NHS	National Health Service
NICE	National Institute for Clinical Excellence
NIHR	National Institute for Health Research
OLE	Open Label Extension
PANAS	Positive and Negative Affect Scale
PIN	Patient Identification Number
PO	Per Os
PPI	Pre-Pulse Inhibition
PsiDeR	Psilocybin in Depression Resistant to treatment

QIDS-SR-16	Quick Inventory of Depressive Symptoms
REC	Research Ethics Committee
RH	Relative Humidity
RR	Respiratory Rate
SAE	Serious Adverse Event
SaO2	Blood Oxygen Saturation
SAP	Statistical Analysis Plan
SAR	Serious Adverse Reaction
SCL	Symptom Check List
SOC	System Organ Class
SOP	Standard Operating Procedure
SP	Screening & Preparation
SSRI	Selective Serotonergic Reuptake Inhibitor
STAR-C	Scale to Assess the Therapeutic Relationship (Clinician)
STAR-P	Scale to Assess the Therapeutic Relationship (Patient)
STAI	State Trait Anxiety Inventory
TAS	Tellegen Absorption Scale
TNF-α	Tumour Necrosis Factor Alpha
TRD	Treatment Resistant Depression
Trial Management Group	TMG
TSC	Trial Steering Committee
UAR	Unexpected Adverse Reaction
UN	United Nations
USAR	Unexpected Serious Adverse Reaction
YMRS	Young Mania Rating Scale
µg	Microgram

Background & Rationale

Need

Major depressive disorder (MDD) is a disabling and economically costly mental health problem. Its prevalence in England is estimated to be between 29 and 42 per thousand people [1]. The estimated cost in England is estimated by the UK government to be £20.2-23.8 billion per year [2]. Treatment resistant depression (TRD), defined as failure to respond to at least two antidepressants at therapeutic dose for at least 6 weeks, is seen in 1/3 of those who suffer from clinical depression and as resistance to treatment develops, prognosis worsens [3]. Healthcare expenditure escalates quickly with treatment resistance [4] because A) patients are more likely to be chronically psychosocially disabled and economically inactive, B) patients are less likely to respond to subsequent treatments, C) patients are more likely to require hospitalisation and, D) risk of suicide attempt and completion are increased, with resulting psychosocial and economic burden on affected relatives. Therefore, there is a pressing need to develop treatments for resistant forms of MDD.

Despite this, there have been no new breakthrough pharmaceutical treatments for MDD since the introduction of the selective serotonin reuptake inhibitors (SSRIs) in the late 1980s. Many pharmaceutical companies have ceased drug development efforts in psychiatry altogether [5]. In response, clinicians and researchers have looked to history for inspiration.

Background & Rationale

The classical psychedelic drugs, including psilocybin and d-lysergic acid diethylamide (LSD), were used by psychiatrists in the treatment of mood disorders before prohibition in 1970 [6]. They were established as safe for use in medically controlled settings [7-9], with no evidence of end organ damage even after massive overdose and chronic usage [10]. They were found to be particularly helpful in psychologically supportive settings in those with MDD, anxiety and addictions [11]. They were found to be unhelpful for those with existing psychotic disorders, or those liable to develop them, however screening procedures to remove these individuals from treatment minimised this risk; observational studies found that the rate of psychosis in both healthy participants and psychiatric patients was no greater than might be expected with placebo [8, 12]. This has been confirmed with modern research using web questionnaires in recreational users [13] and two modern population based studies in the US have found psychedelic use to be associated with *lower* rates of inpatient mental health treatment [14], psychological distress and suicidality [15], with no increased rate of psychosis [16].

We recently completed a systematic review of the pre-prohibition literature detailing trials of psychedelics in patients with depression [17]. Whilst pre-prohibition studies were unsystematic and poorly designed, in aggregate 335 of 423 (79.2%, median 80.0%) patients in 19 separate studies were judged by their clinicians to have at least 'improved' with psychedelic therapy.

After prohibition, medical use ceased and research dwindled [18]. Over the past 10 years, for the reasons outlined in the previous section, interest in the therapeutic application of the classical psychedelics has re-emerged within the context of the safeguards to use implicit in legal regulation.

Psilocybin (4-phosphoryloxyl-N,N-dimethyltryptamine) is a tryptamine compound found in a variety of mushrooms of the psilocybe genus, which grow ubiquitously in the UK and all over the world. Psilocybin mushrooms have been used in healing rituals and as spiritual and ceremonial sacraments for thousands of years, perhaps most notably by ancient South American civilisations.

Psilocybin was isolated and synthesised by Albert Hofmann in 1957 and 1958, respectively [19]. Similar to all classical psychedelics, psilocybin is a partial agonist at the 5-HT2a receptor in the CNS and a variety of lines of evidence suggest that it is this pharmacological property that accounts for the subjective effect [20], although its agonist activity at serotonin receptors is broad (except for 5-HT3) [21]. It has low or absent affinity for the serotonin transporter, dopaminergic, histaminergic, adrenergic or cholinergic receptors, an observation that may underlie the absence of known toxic effects on the body, particularly the serotonergic syndrome (ibid.). The toxic ratio (ratio of the estimated lethal dose to the standard psychoactive dose) is estimated at 1,000 [22]. This compares favourably to a figure of 100 for fluoxetine, which is one of the least toxic drugs prescribed routinely in psychiatry. The LD50 in animals ranges from 280mg/kg in rats to 12.5mg/kg in rabbits [23]. No human research so far has used more than 0.4mg/kg. The most common physiological effects are mild increases in heart rate and blood pressure, and mydriasis [24].

In a review of psilocybin administration to 110 healthy subjects, prolonged dysphoric experiences ('bad trips') were rare and dose dependent and there was no evidence of subsequent drug abuse, flashback phenomena or prolonged psychosis [25]. Psilocybin is not habit forming in either humans or animals [26], with no capacity to induce dependence, a property shared with all classical psychedelics.

Since 2006, pilot clinical studies in anxiety associated with advanced cancer [27, 28], obsessive compulsive disorder [29], tobacco [30] and alcohol [31] addiction and cluster headaches [32] have been completed, with encouraging results. In December 2016, two randomised, placebo controlled trials of psilocybin in depression and anxiety associated with advanced cancer were published from US groups in New York and Boston, both showing rapid, substantial and sustained reductions in anxiety and depression in the group treated with psilocybin [33, 34].

Pilot Work

In TRD, we have completed an open label pilot study of oral psilocybin in adults in May 2016 [35, 36]. 20 patients with depression refractory to at least 2 courses of antidepressants were given two oral doses of psilocybin (10mg and 25mg) 1 week apart in a clinical research facility with psychological support before, during and after the experience. The treatment was well tolerated, with no serious adverse events. The 25mg dose was well tolerated by all of the patients; no serious or unexpected adverse events occurred, and the mean patient rating of intensity of drug effects was 7.5 (+/- 2.5) out of 10, within the range of expected intensity.

Patient rated depression severity was markedly reduced in comparison to baseline at 1 week after 25mg psilocybin (Cohen's $d=2.2$, $p<0.001$) and 5 weeks (Cohen's $d=2.3$, $p<0.001$), remaining positive at months 3 and 6 following treatment (Cohen's $d=1.5$, $p<0.001$, and $d=1.4$, $p<0.001$, respectively) (Figure 1). None of the participants sought conventional antidepressant treatment within 5 weeks of treatment. Aside from

transient global headache, lasting an average of 12 hours and responsive to simple analgesia with paracetamol, no adverse events were noted in the immediate follow up period. Based on these observations and further conversations with teams delivering psilocybin as a treatment to healthy volunteers at Imperial College London and patients at Johns Hopkins University (USA) and New York University (USA) we are confident that the 25mg per oram dose maximises the potential for therapeutic benefit, whilst minimising the risk of acute adverse events.

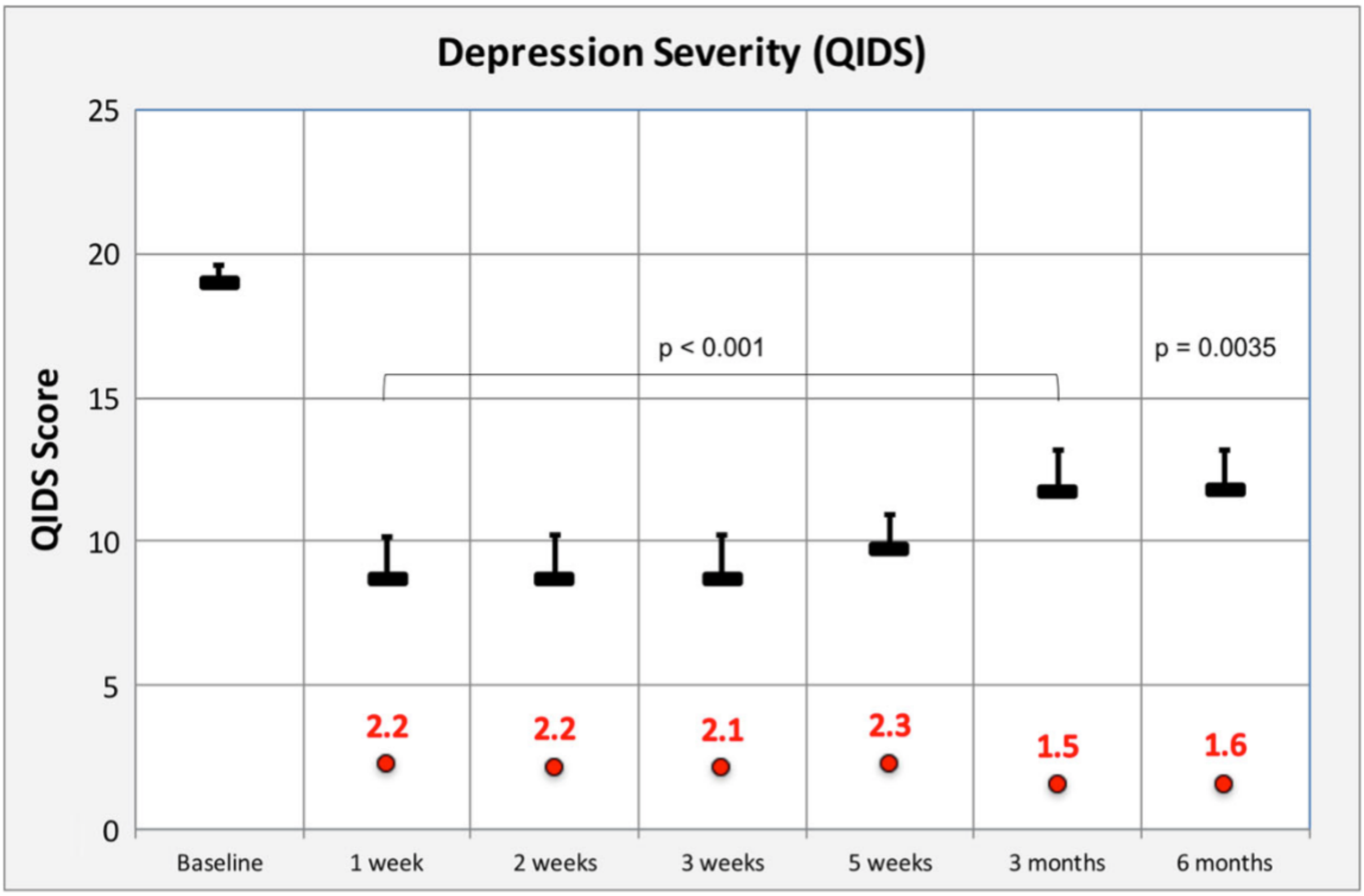

Figure 1. **Mean depression severity (QIDS) over time.** Depression severity determined by self-rated 16-item QIDS. QIDS scores of 16–20 are considered to reflect severe depression, scores of 11–15 are considered moderate depression, scores of 6–10 are considered mild depression, and scores of 5 and less are considered absent depression. All post-treatment assessments were obtained after the high-dose session (i.e., 1 week post-treatment refers to 1 week after the high-dose session). Hedges' g values versus baseline are shown. QIDS=Quick Inventory of Depressive Symptoms. Taken from (34).

The pilot study established the protocol as pragmatic within the current UK legal framework and provided evidence of safety. However, this study was not blinded and did not have a placebo control group. Thus, it remains possible that expectation and/or the psychological support provided as part of the trial account for the improvements in depression scores. Thus, the aim of this research is to conduct a blinded, randomised, placebo-controlled trial of psilocybin in participants with TRD to further investigate feasibility, safety and efficacy in this patient group.

Trial Description

Note: In this section follows a comprehensive description of the clinical trial protocol. Elements of this description will be repeated subsequently with additional detail as required under the appropriate headings.

A full description is included here to facilitate the reader developing a narrative 'sense' of the trial and its procedures.

Trial Design

This will be a **two arm, double-blind, randomized, placebo controlled, between-subjects design in up to 60 completing participants meeting Diagnostic and Statistical Manual of Mental Disorders (5th Edition) (DSM-5) criteria for single or recurrent episodes of major depressive disorder (MDD) of at least moderate severity (but without psychotic features) who have failed to respond to EITHER 2 or more antidepressants prescribed at the minimum effective dose for at least 6 weeks OR at least 1 antidepressant prescribed at the minimum effective dose for at least 6 weeks AND a course of evidence-based psychological therapy given for at least 6 sessions**.

This is a study with a central 'core' element and three optional arms (see Fig. 3). The core element is the randomised controlled trial of psilocybin in TRD in up to 60 completing participants with 6 weeks of follow up. Three 'orbiting' elements can then be 'bolted on' according to participant wishes/consent. If participants consent to involvement with these optional elements, then they will be free to withdraw at any time without affecting their ability to continue in the core trial. The optional elements are as follows:

1. Collection of additional biological samples (blood).
2. Neuroimaging.
3. An open-label extension with 1 further dose of 25mg psilocybin after 6 weeks of follow up and 12 weeks of subsequent follow up.

These three elements are discussed in more detail subsequently.

Recruitment

Trial participants will be recruited by a variety of methods, including secondary and primary care referrals, NHS IAPT (Improving Access to Psychological Therapies) services, established clinical trial registries of patients who have consented to contact from study teams, word of mouth and directly from advertising in the community and on social media.

Screening

Potential trial participants will be initially screened by telephone or online survey by a member of the study team. Those who are deemed to be potentially eligible will be invited for a face to face screening assessment with a member of the study team. Final study inclusion and consent will be taken by a study psychiatrist.

The following inclusion criteria will apply:

- Age 25 - 80 years
- Fluent in the English language
- Fulfil Diagnostic and Statistical Manual of Mental Disorders (5th Edition) (DSM-5) criteria for a primary diagnosis of current single or recurrent episodes of MDD of at least moderate severity but without psychotic features as defined on the MINI 7.0. Positive and primary diagnoses on the MINI 7.0 will be subject to confirmation at clinical interview by a psychiatrist.

- 17-item HAM-D score ≥ 14.
- Have failed to respond to 2 or more antidepressants prescribed at the minimum effective dose for at least 6 weeks OR at least 1 antidepressant prescribed at the minimum effective dose for at least 6 weeks AND a course of evidence-based psychotherapy given for at least 6 sessions.
- For those aged ≥ 60 years, the first episode of depression must have started prior to their 60th birthday.

'Failed to respond' is defined as an inadequate response to an adequate duration and dose OR failure to reach an adequate dose and duration due to lack of tolerance. Thus, an antidepressant that was taken for less than six weeks but discontinued due to intolerable side effects will be counted as a single treatment, regardless of treatment duration. Adequacy of response and dose and duration of treatment will be assessed using the Maudsley Treatment Inventory [37].

'Antidepressant' refers to any drug contained within the most recent version of the British National Formulary that is also licensed for the treatment of MDD or any treatment for MDD listed within the latest (May 2018) version of the Maudsley Prescribing Guidelines, and as summarised in the Maudsley Treatment Inventory [ibid.]. Antidepressant augmentation medication (for example, the addition of mirtazapine, quetiapine, lamotrigine or lithium to an existing antidepressant) will count as a second treatment for the purposes of this protocol **provided that the criteria for adequacy of dose and duration of each separate treatment (as defined by the Maudsley Treatment Inventory [ibid.]) still apply.**

'A course of evidence-based psychological therapy' is defined in reference to revised NICE guidelines for MDD as one of the following:

- Group-based Cognitive Behavioural Therapy
- Individual Cognitive Behavioural Therapy
- Behavioural activation
- Interpersonal Therapy
- Counselling
- [Short-term] psychodynamic therapy
- Behavioural couples therapy
- Mindfulness Based Cognitive Therapy

To qualify, the following conditions must be met

1) The psychological therapy was delivered for the primary purpose of treating depression (+/- anxiety) in the participant.
2) The psychological therapy was delivered by a qualified clinical or counselling psychologist or psychotherapist or psychological wellbeing practitioner.
3) The participant attended for at least 6 sessions of at least 45 minutes each.

Note: Criterion 3 deviates from NICE guidelines, which recommend 12-16 sessions of therapy. We have chosen 6 sessions because in practice this is the minimum number of sessions that is usually offered by NHS IAPT services. This point was specifically discussed and agreed with service user representatives of

the trial steering committee, who felt that it would be discriminatory to exclude potential participants on the basis of a service failing to provide NICE recommended number of sessions.

The following exclusion criteria will apply

- Diagnosis of bipolar disorder (defined as meeting DSM-5 criteria for bipolar 1 or bipolar 2) on the MINI 7.0. Positive diagnoses on the MINI will be subject to confirmation at clinical interview by a psychiatrist.
- Diagnosis of psychotic disorder (defined as meeting DSM-5 criteria for any psychotic disorder) on the MINI 7.0, EXCEPT substance/medication induced psychotic disorder where the duration was limited to the acute period of direct intoxication with the substance/medication. Positive diagnoses on the MINI will be subject to confirmation at clinical interview by a psychiatrist.
- Diagnosis of drug or alcohol dependence syndrome (defined as meeting DSM-5 criteria for any dependence syndrome) on the MINI 7.0. Positive diagnoses on the MINI will be subject to confirmation at clinical interview by a psychiatrist.
- Diagnosis of any personality disorder (defined as meeting DSM-5 criteria for any personality disorder) based on clinical interview and the MINI 7.0. Positive diagnoses on the MINI will be subject to confirmation at clinical interview by a psychiatrist.
- Diagnosis of any dementia (defined as meeting DSM-5 criteria for any dementia disorder) based on clinical interview by a psychiatrist.
- Personal history of a ≥ 1 suicide attempt in the past year requiring hospitalization, defined using the CSSRS (Q6 (past year) = "y") and confirmation at clinical interview with a psychiatrist.
- Other personal circumstances and behaviour judged to be incompatible with establishment of rapport or safe exposure to psilocybin.
- Depression secondary to other medical conditions
- Medical diagnosis incompatible with psilocybin treatment (see Section 6.2.1)
- Inability to provide a screening blood sample, urine sample or electrocardiogram.
- Biochemical abnormalities (defined as falling outside the normal reference range) as evaluated by a full blood count, full biochemistry profile and thyroid function tests. Biochemical abnormalities must also be determined as clinically significant by a medical doctor to fulfil the criterion for exclusion.
- Electrocardiographic abnormalities, defined as any abnormality that is not normal sinus rhythm and determined as clinically significant by a medical doctor.
- Women of child bearing potential not using adequate contraception (see Section 6.2.2).
- Pregnant or breast-feeding women.
- Those unable to give informed consent.

- Non-registration with a GP or failure to consent to sharing of the GP summary care record and any psychiatric assessments held.
- Those enrolled in another drug trial
- Hypersensitivity to the IMP or to any of the excipients or placebo

Treatment resistance will be further defined by the Maudsley Staging Method, which implements a 15 point scale that scores various aspects of the clinical history that define treatment resistance [38]. Participants must score **at least 5/15** on this scale to be eligible for the trial, with no upper limit on the degree of treatment resistance. Treatment resistance will be further defined by the Maudsley Treatment Inventory (MTI), which defines the nature and adequacy of dose and treatment duration of antidepressant and antidepressant augmentation treatments named according to established NICE guidelines, the British National Formulary (BNF) and the Maudsley Prescribing Guidelines [37].

Note. Our recruitment criteria are deliberately chosen to balance three factors:

1) The pragmatics of recruitment to trials in TRD, particularly recruiting and retaining those who are currently depressed.
2) The need to focus emerging therapy research on those who are multiply treatment resistant, who tend to be particularly morbid and/or socioeconomically costly, but also a particularly difficult group to recruit and retain.
3) The demographic of patient that this type of treatment may appeal to is less likely to have substantially engaged in existing antidepressant therapy

To be eligible for this study participants will need, in addition to satisfying the eligibility criteria, to agree to the following:

1. Providing contact details of a trusted friend or relative that the study team may contact in the event of an emergency.
2. To stay within the boundaries of the King's Clinical Research Facility/CMHRI during the Dosing Visit (the day of treatment) for at least 6 hours after the IMP is given, or until it is clinically determined that they are safe to leave, whichever is longer. The rationale for this is expanded upon in Section 3.3.7.

Trial Flowchart & Structure

This is a study with a central 'core' element and three optional arms (see Fig. 3). The core element is the randomised controlled trial of psilocybin in TRD in up to 60 completing participants with 6 weeks of follow up. Three 'orbiting' elements can then be 'bolted on' according to participant wishes/consent. If participants consent to involvement with these optional elements, then they will be free to withdraw at any time without affecting their ability to continue in the core trial. The optional elements are as follows:

1. Collection of additional biological samples (blood). We will collect up to 5 additional sets of blood samples in each participant for storage and subsequent analysis. Each sample set may consist of a maximum of 50mls of blood, separated into EDTA, serum, plasma and paxGene tubes as necessary. The purpose of collecting these samples is to store serum, plasma, buffy coat, DNA and RNA for separate mechanism analyses. For example, with these samples we may explore immunological

correlates of response to treatment with psilocybin because previous research has shown both that psychedelics potently suppress a variety of immune related biological markers [39, 40] and because response to treatment for depression [41], as well as depression itself, is correlated with changes in these markers. These analyses will be performed separately, require separate authorisation and these analyses are not an intended outcome of this project. Samples will be securely stored at the Sponsor's laboratories. The custodian of the samples will be Dr James Rucker.

2. Neuroimaging. As part of this study we will offer participants the opportunity to undergo two 1 hour MRI scans before and after treatment. We have hypothesised that there will be specific changes in amygdala BOLD responses to emotional faces and changes in glutamergic and/or GABAergic tone in the medial prefrontal cortex and/or anterior cingulate cortex when pre-treatment and post-treatment measures are compared between groups. This analysis will be performed separately and is not intended to be an outcome for the present study. A separate PIS and consent form is available for this part of the study.
3. An open-label extension to this trial. We will offer participants who complete 6 week follow up and remain eligible the option to receive an open-label dose of 25mg of psilocybin, delivered under similar conditions, with a further 12 weeks of remote and face-to-face follow up. A separate PIS and consent form is available for this part of the study.

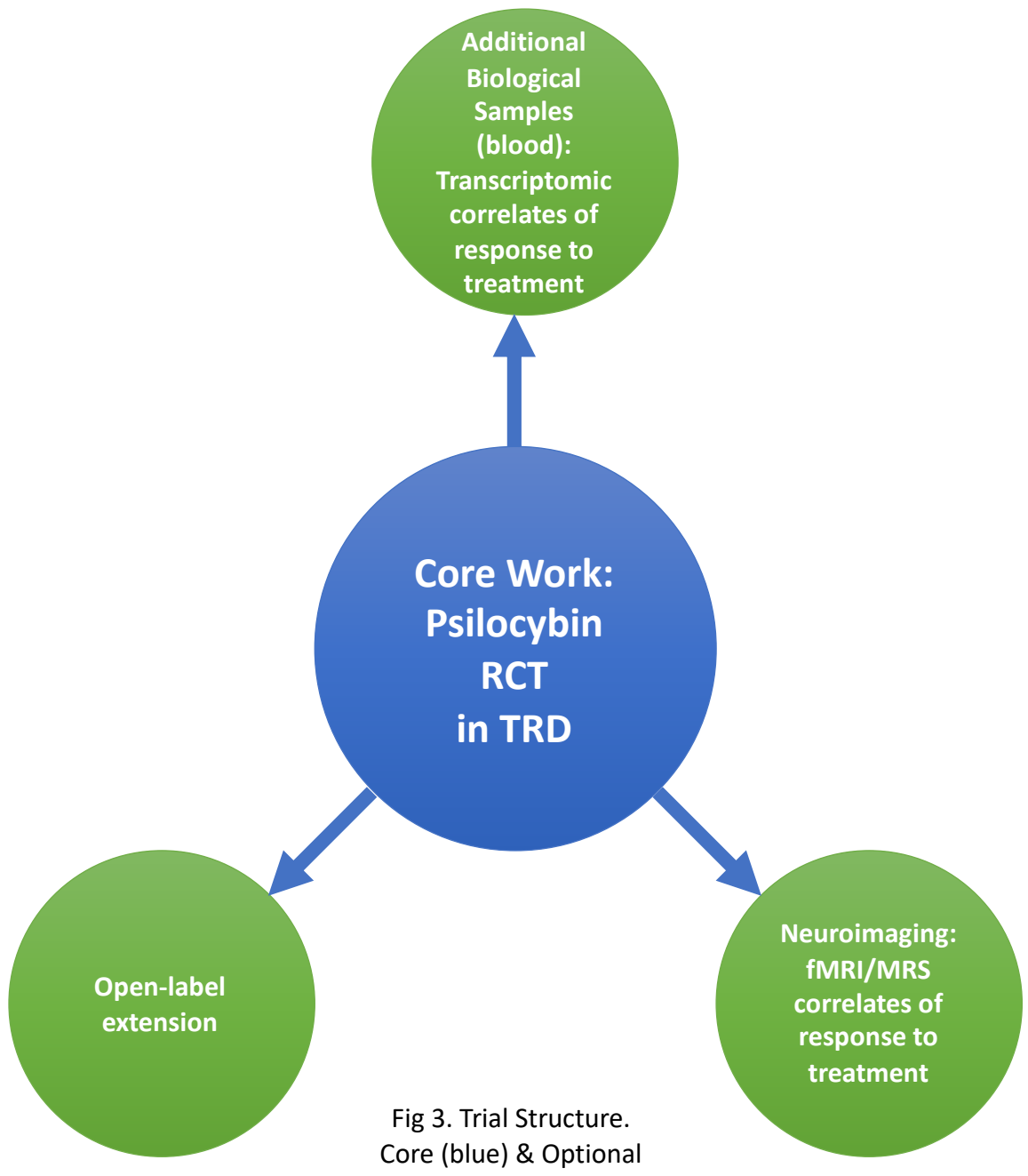

Fig 3. Trial Structure. Core (blue) & Optional (green) Elements

The following schematic pictorially represents a participant's journey through the trial, with the subsequent figure depicting the overall trial structure. All study visits will take place at the NIHR/Wellcome Trust Clinical Research Facility at King's College Hospital, Denmark Hill, London, UK or at the IoPPN/SLaM/Compass Pathways Centre for Mental Health Research and Innovation, 5 Windsor Walk, London, UK.

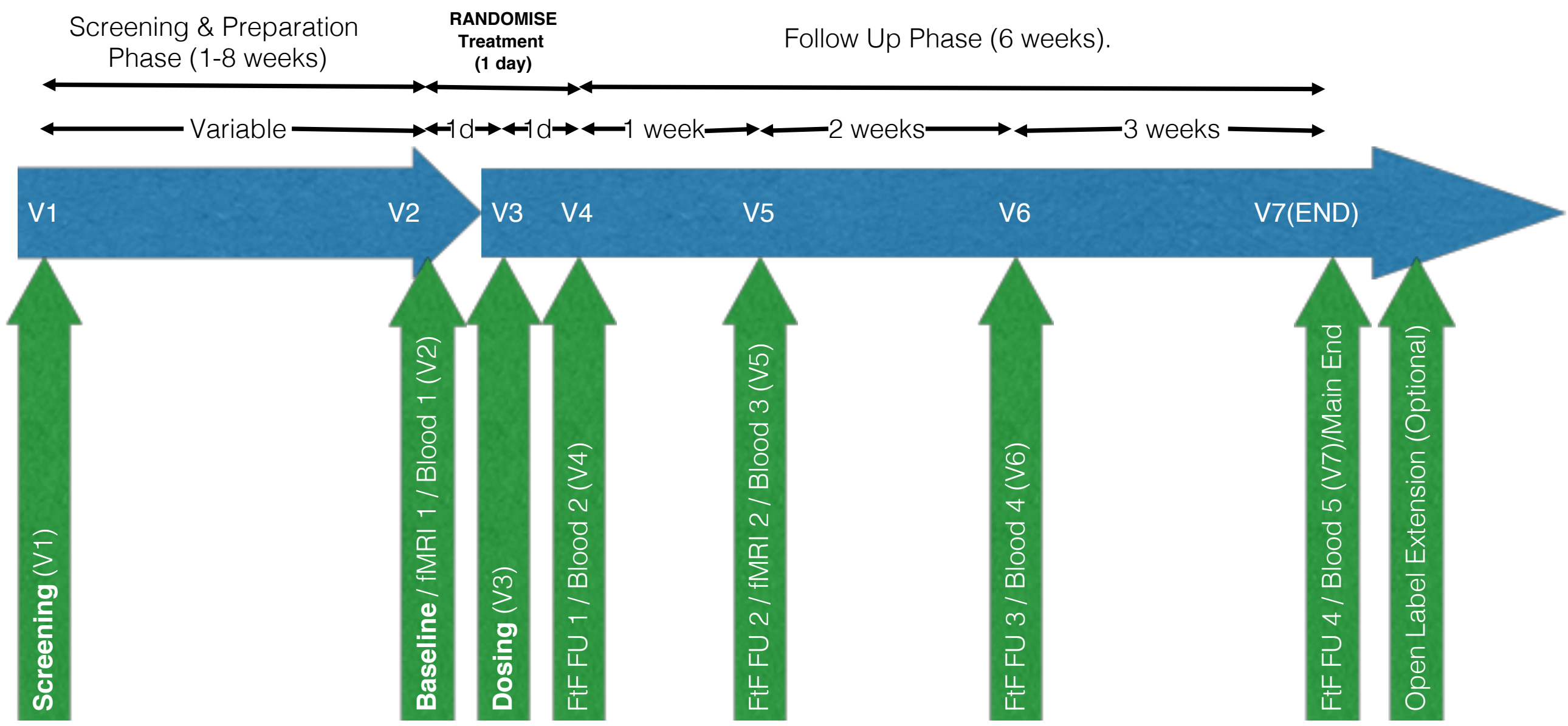

Fig. 2. Study Schematic
Abbreviations: FtF, Face to Face; FU, Follow Up; V, Visit

Screening & Preparation (SP) Phase (1-8 weeks)

Participants who are deemed eligible at the screening visit will be assessed by a study psychiatrist and enter a Screening & Preparation (SP) phase of between 1-8 weeks. The variation in time reflects a number of clinical scenarios. For example, some participants will require time for existing treatments to be withdrawn. This is particularly relevant for the commonly prescribed antidepressant fluoxetine, which has a long biological half-life and hence requires an 8-week wash out period. Similarly, we will need to seek collateral information from GPs and CMHTs (which can often be a slow process) to confirm inclusion and exclusion criteria in some participants. Other participants may require additional face to face psychoeducation and preparation time, particularly if they are naïve to psilocybin. Conversely, some participants may be recruited from 'ready-to-go' repositories of patients with TRD where their clinical status and the ready availability of collateral information will enable a more rapid journey through the SP phase. Similarly, some participants may be familiar with psilocybin through recreational use (although we will seek to keep the overall proportion in the sample with prior experience similar to the population prevalence of use). Regardless, all participants will receive the same screening assessments and baseline psychological preparation prior to receiving treatment. For details, please refer to the Psychological Support Model document. We will use, with permission granted, psychoeducation materials and videos already developed by Compass Pathways for their multicentre TRD trial using psilocybin.

Withdrawal from antidepressant, antipsychotic or mood stabilising treatments will be supervised by a clinician, flexible to the needs of the participant. This step is required because standard antidepressant and augmentation medications attenuate or block the effects of psilocybin. During the SP phase, participants will be regularly reviewed, either face to face or by telephone. We will extend the screening and preparation phase for a further period of up to 8 weeks if participants require more time to withdraw from their

antidepressants, or if collateral history suggests a medical or personal problem that has not been adequately investigated, treated or accounted for, or if participants require more preparation with their therapists prior to the treatment.

We have established minimum follow up requirements for participants in the screening and preparation phase as follows. 1. All participants will receive at least 3 hours of psychological preparation over at least 2 separate sessions with a dedicated therapist. 2. All participants who do not have a care giver will be monitored for emergent or worsening suicidal ideation and general wellbeing in a weekly clinic visit with telephone monitoring calls every four days. 3. Participants with a care giver will be monitored weekly either by clinic visit or telephone call. We have established escape criteria in Section 3.3.15 that outline in what scenario we would recommend to participants that they restart the best available treatment.

For those participants that have a care giver, we will ask them to contact us if they think that the participant's mental health is significantly deteriorating or that they believe that are at significant risk to themselves or others.

Participants will be given a contact card with which they can contact a member of the study team for the duration of their journey through the trial. We have an established system of out-of-hours cover for our department's studies via a 24-hour mobile phone that allows access to a clinician knowledgeable about the trial. Clinical status and risk will be monitored in liaison with a participant's GP and/or CMHT and/or psychotherapist.

It is anticipated that a proportion of participants will drop out during the SP phase and measuring this is part of the purpose of conducting this trial. Reasons for drop outs will be recorded and reported to the independent TSC and DMEC, who will advise the Sponsor on the progress and conduct of the trial. Substantial amendment may be sought in light of this data.

During the SP phase, participants will be given details of the trial website (https://psider.info) that will contain information about the trial (including the participant information sheet), contact details for the study team, and frequently asked questions. The website will be maintained by the study team and web designer (who is a member of the trial steering committee who also has lived experience of both depression and psilocybin therapy). Use of this website is not compulsory but participants will be encouraged to engage with it, as it will serve as a centralised repository of information about the trial, the treatment, the schedule of assessments and visits and the schedule of patient and public engagement about the trial and its results.

During the SP phase, participants will be introduced to a therapist and a chaperone familiar with psilocybin and its effects. The therapist and/or chaperone will provide face to face support for participants on, at a minimum, visits 2, 3 and 4 (V2, V3, V4). The purpose of this is; to provide psychoeducation, establish rapport and address anxieties prior to the treatment (V2); to provide appropriate care and support to participants during the treatment (V3); to provide an opportunity for psychological support and integration after the experience (V4, V5 & V6). We expand upon the particular models of support subsequently. Other sessions with therapists and/or chaperones may be provided either face to face (preferred) or remotely and may be provided on date other than the visit date if desired by the participant or if logistically necessary. Depending

on participant needs we will arrange additional sessions for psychological preparation and integration as required. This reflects a degree of clinical flexibility required to ensure participant's adequate preparation and integration, which will vary depending on their prior history and current circumstances. It also reflects the fact that psychotherapeutic support in this trial is being provided, in the main, by volunteers. Each volunteer has appropriate credentials (registration with an appropriate professional body and an honorary contract with the sponsoring NHS Trust) and will have been adequately trained in the principles we describe below. The number of sessions and total number of hours of psychological support for each participant will be recorded and may be used as an independent variable in the analysis.

In addition, if participants request additional support during their journey through the trial, then we will ask therapists to provide this. Similarly, a psychiatrist will be available to monitor clinical status, risk and medication. This element of the protocol is so-designed to balance the needs of the participant (primary) with the needs of the trial (secondary). Further details about the model of integration used in this trial can be found in Section 7.1.3.6.

Baseline Visit (V2)

Prior to the Dosing Visit (V3), participants will be invited to attend for the Baseline Visit (V2). Final eligibility will be confirmed at this visit, considering all the information available to the study team. The study team will reserve the right to withdraw a participant from the trial at this stage if it has become apparent during the SP phase that the participant is not suitable to be given psilocybin treatment. We have taken this step in light of infrequently occurring scenarios in the pilot trial where it became apparent that a participant's motivations for trial participation were opaque and/or not consistent with collateral information.

At the Baseline Visit (V2), we will ask participants to attend the CRF. Depending on consent, we will draw a blood sample for biological analysis (optional additions). We will perform baseline clinical procedures and measures of outcome and functioning as described in the schedule of assessments. After a short break, participants will meet with their therapist and chaperone to prepare them for the Dosing Visit (V3). Depending on consent, we will ask participants to undergo a 1-hour fMRI scan. If required, participants will be offered a 'practice' session in a sham MRI scanner to determine if they feel comfortable enough to undergo the scan, which can induce feelings of claustrophobia. During the fMRI scan we will ask participants to view a number of emotionally charged and emotionally neutral facial expressions whilst we collect functional MRI data. The emotional faces paradigm is a widely used and accepted method. We will also collect resting state data, structural scans and magnetic spectroscopic scans of glutamatergic and/or GABAergic activity in the medial prefrontal and/or anterior cingulate cortex, during which time participants will be asked to lie quietly in the scanner. It is anticipated that this visit will last approximately 4-6 hours (depending on consent for optional additions). We may perform the fMRI scan 1 day either side of the baseline visit, if this is preferred by the participant or if it is necessary because of other logistical reasons (such as the availability of the scanner).

Dosing Visit (Treatment) (V3)

The Dosing Visit (V3) will take place 1, 2 or 3 days after the Baseline Visit (V2).

Up to **60 participants will be randomly allocated to two groups of 30**. Participants will be randomised at V2, after final eligibility has been confirmed by a study medic. Participants will receive a **single dose** of either:

1) **25mg of psilocybin, per os**
2) **Placebo**

Participants and the study team will be blind to treatment allocation. Psilocybin capsules (or tablets) and identical placebo capsules (or tablets) will be provided by Compass Pathways Ltd., who will supply the dispensing pharmaceutical company. The dispensing pharmaceutical company will be Fisher Clinical Services UK Ltd. (Langhurstwood Road, Horsham, West Sussex, RH12 4QD, UK), who possess the required Schedule 1 pharmaceutical manufacturing licenses that allow storage, packaging and labelling of the psilocybin containers. Randomised treatment allocation will be performed by the King's College London Clinical Trials Unit and passed electronically to Fisher Clinical Services. The treatment will be delivered within 5 identical, opaque, HPMC capsules (or tablets) for oral administration. The trial PI (Dr James Rucker) and CI (Professor Allan Young) hold the relevant Schedule 1 prescribing licenses issued by the UK Home Office, who will also hold a list of those authorised to prescribe and administer psilocybin. A prescription will be delivered to the Maudsley Pharmacy, which holds a Schedule 1 possession and dispensing license. The capsules (or tablets) will be picked up by a member of the study team immediately before administration on the Dosing Visit and the empty dosing bottle delivered back to the pharmacy on the same day. Any unused capsules will be quarantined and disposed with as per pharmacy policy for Schedule 1 drugs.

Both treatments (25mg psilocybin or placebo) will be delivered in identical settings at the NIHR Clinical Research Facility (CRF) at King's College Hospital or at the CMHRI.

On the Dosing Visit (V3), the treatment will be transported in a locked box to the King's CRF by a member of the study team. Participants will arrive in the morning to meet their therapist and chaperone. We will perform a brief mental state exam, before allowing sufficient time for participants to relax and for any anticipatory anxieties or practical considerations to be addressed. Administration of the treatment will occur once the participant is relaxed and the study team have addressed any practical issues.

The environment within which the treatment will be given will be a quiet, neutrally furnished room with adjustable lighting in the King's Clinical Research Facility/CMHRI. A reclining seat or bed will be provided that participants can lie down on, as well as two chairs for the therapist and chaperone. Easy access to the lavatory and light refreshments will be available. Participants will be given an eye mask and ear phones through which they will be able to hear a curated playlist of relaxing music. The therapist and chaperone will be present in the room, with at least 1 person with the participant at all times during the Dosing Visit. We will ask participants to swallow the treatment with a glass of water in the morning. The Dosing Visit and other study visits may be discreetly filmed via a video or audio recording system; however participants will have the option to ask us to switch this off if they feel uncomfortable.

After the treatment has been swallowed, we will require participants to stay within the boundaries of the King's Clinical Research Facility/CMHRI until it is clinically determined that they are safe to leave. This requirement acknowledges the possibility that some participants may have to be prevented from leaving the

King's Clinical Research Facility/CMHRI in pursuance of their best interests if, in the determination of the study team, they pose a risk to themselves or others. This will be specifically discussed and consented at screening. Whilst a deprivation of liberty akin to this did not occur in the pilot trial, within the context of psychoactive drug trials in participants with current mental health difficulties, the possibility is nonetheless acknowledged and discussed here.

Any actions taken to minimise a participant's risk to themselves or others and/or prevent them from leaving the King's Clinical Research Facility/CMHRI will be undertaken in accordance with the tenets of appropriate legislation, in particular the Mental Capacity Act (2005), the Mental Health Act (1983/2007) and the Common Law Doctrine of Necessity. If necessary, and as a last resort, in the event of extreme distress unresponsive to therapist intervention, or in the event of threatened or actual violence, rescue medication will be given in liaison with the ward nursing and medical staff at the CRF. The medication of choice is a short acting benzodiazepine given orally and therefore follows established NICE guidelines for rapid tranquilisation. We will use oromucosal midazolam 2.5mg-5mg per os as a first line. Any further treatment will follow NICE guidelines. This will be given under the supervision of a psychiatrist and, if necessary, overnight hospital admission will be arranged for observation, with review by the study team the following morning and reporting of the event to appropriate pharmacovigilance authorities.

If participants receive the 25mg dose of psilocybin the subjective effect will peak at approximately 2 hours and then wear off over a further period for approximately 2-4 hours. That is to say, if participants take the treatment at 10:30, we expect the vast majority to have returned to baseline by 16:30. Inter-individual variation is, however, expected and therefore participants will be accompanied for as long as necessary by the study team.

A medical doctor will determine when the participants have returned to baseline. We will then allow time for discussion, give participants a meal if desired and then we will allow them to travel home either alone in a taxi or by another method of transport accompanied by a friend or relative. If participants have a long journey home or if the discharge is late at night then overnight accommodation in a local hotel or the trial facility may be arranged.

Follow Up Phase (6 weeks)

The follow up period after treatment will be a total of 6 weeks. A member of the study team will see participants for face to face follow up at 1 day (V4), 1 week (V5), 3 weeks (V6), and 6 weeks (V7).

We will ask (although not require) participants to undergo the following procedures during the follow up period:

1) MRI scan (duration 1 hour) at 1 week after treatment (V5). Identical tests will be administered to those administered at V2.
2) Donate a blood sample for biological analysis. A blood sample as described in Section 3.3.4 will be taken 1 day prior to the treatment (V2), 1 day after the treatment (V4), 1 week after the treatment (V5), 3 weeks after the treatment (V6) and 6 weeks after the treatment (V7).

Follow Up Visit 1 (V4). 1 Day After Treatment.

Participants will attend the CRF in the morning or afternoon. The allowable window for this visit is +1 day. Consent depending, we will draw a blood sample as described in Section 3.3.4. We will ask participants to complete self-report measures as described in the schedule of assessments. A member of the study team will perform study team-rated measures as described in the schedule of assessments. Participants may meet with their therapist for up to 90 minutes to discuss their experience of the treatment and to allow time for the therapist to work with the participant to help them process any difficult material that may have arisen. The chaperone may join this session. It is anticipated that this visit will last approximately 4 hours.

Follow Up Visit 2 (V5). 1 Week After Treatment.

Participants will attend the CRF in the morning or afternoon. The allowable window for this visit is +/-1 day. Consent depending, we will draw a blood sample as described in Section 3.3.4. We will ask participants to complete self-report measures as described in the schedule of assessments. A member of the study team will perform study team-rated measures as described in the schedule of assessments. Participants will meet with their therapist for up to 90 minutes to discuss their experience of the treatment and to allow time for the therapist to work with the participant to help them process any difficult material that may have arisen over the preceding week. The chaperone may join this session. Participants will then be invited to undergo a 1 hour MRI scan at the Centre for Neuroimaging Sciences (5 minutes' walk from the King's CRF). During the MRI scan, we will show participants images of emotionally neutral and emotionally charged facial expressions via an established and well-validated paradigm, over a period of approximately 20 minutes. For the remaining 40 minutes we will collect resting state data, structural scans and magnetic spectroscopic data of glutamatergic and/or GABAergic activity in the medial prefrontal cortex and/or anterior cingulate cortex. Collection of this data will not require participants to actively do anything other than lie quietly in the scanner. It is anticipated that this visit will last approximately 6 hours, consent depending. We may perform the fMRI scan 1 day either side of the visit, if this is preferred by the participant or if it is necessary because of other logistical reasons (such as the availability of the scanner).

Follow Up Visit 3 (V6). 3 Weeks After Treatment.

Participants will attend the CRF in the morning or afternoon. The allowable window for this visit is +/-2 days. Consent depending, we will draw a blood sample as described in Section 3.3.4. We will ask participants to complete self-report measures as described in the schedule of assessments. A member of the study team will perform study team-rated measures as described in the schedule of assessments. Participants may meet with their therapist either face to face or virtually at or around this visit. The chaperone may join this session. It is anticipated that this visit will last approximately 4 hours.

Follow Up Visit 4 (V7). 6 Weeks After Treatment.

Participants will attend the CRF in the morning or afternoon. The allowable window for this visit is +7 days and -3 days. The rationale for this is to allow the flexibility for visit schedules that take account of scheduled holidays, such as Christmas and Easter, without the necessity of submitting a protocol deviation. Consent depending, we will draw a blood sample as described in Section 3.3.4. We will ask participants to complete self-report measures as described in the schedule of assessments. A member of the study team will perform study team-rated measures as described in the schedule of assessments. Participants may meet with their

therapist either face to face or virtually at or around this visit. The chaperone may join this session. At this visit we will ask participants to share step counts and sleep data that has been collected by their personal smartphones or fitness watches, if they have them and have indicated their signed consent to this in the PIS. It is anticipated that this visit will last approximately 2 hours, unless participants have indicated their interest in the open label extension (for which they will have been given another PIS to consider). In this case, participants will be assessed by a study clinician and informed consent taken. Additional measures will be taken as described in the Schedule of Assessments. The therapist and chaperone will undertake additional preparation of the participant as necessary.

Open-Label Extension.

Participants who complete follow up visits to 6 weeks and who are deemed eligible by the study team will be offered a 12 week open label extension (OLE) to the trial. Participants who consent will visit the study site on a further 6 occasions, as detailed in Table 5. The inclusion criteria will be

1) Informed consent to this part of the trial.

The exclusion criteria will be

1) Development of exclusion criteria defined in Section 6.2 during the follow up period.
2) Development of personal circumstances and behaviour judged to be incompatible with establishment of rapport or safe exposure to psilocybin during the open label extension.
3) Development of signs of hallucinogen persisting perception disorder, ascertained using the HPPDS, during the follow up period.

The initial preparation and baseline session (OLE1) can be incorporated into V7, as described in Section 3.3.12. At OLE2, every participant will receive a single 25mg dose of psilocybin given under open-label (but otherwise identical) conditions to those described in Section 3.3.7 (OLE 2). OLE2 may take place up 5 weeks after OLE1 in order to achieve sufficient logistical flexibility and in this instance, an additional visit will be undertaken to recheck eligibility, vital signs, pregnancy test (for women) and urine drug screen. Participants will then be seen for face to face follow up at 1 day (OLE 3), 1 week (OLE 4) and 3 weeks (OLE 5), 6 weeks (OLE 6) and 12 weeks (OLE 7). The final visit (OLE 7) may be remote, if desired. Sessions with therapists may be organised remotely and/or around the dates of the follow up visits, depending on participant preferences and logistics. Participants will continue to be supported by the study therapists and psychiatrist in a flexible manner reflecting their evolving circumstances and mental states. Participants will be seen face to face by their therapists and chaperones at OLE1, OLE2 and OLE3. Other visits may include remote therapist support on or around the visit. The purpose of the open label extension is exploratory, and fourfold:

1) Incentivise participants to engage with follow up and not drop out of the study if they believe they were allocated to the placebo condition.
2) To provide data about intensity of subjective experience in participants who may have restarted antidepressant medication in-between V3 and the open label extension.
3) To explore the acceptability and feasibility of delivering >1 dose of psilocybin.
4) To collect further safety data.

Transfer of Care Arrangements

At the end of a participant's final visit, their care will be transferred back to their usual care team. Any additional support that may be required will be arranged in liaison with the usual care team. Clinicians in the participant's usual care team may make clinical enquiries to the study team for advice, if necessary, after discharge.

Escape Criteria and Trial Withdrawal

Some participants may experience a significant deterioration in their mood during withdrawal of antidepressant medication or during the follow up period. Along with a clinical assessment, we will use the following criteria to assess whether participants should be restarted on the best available treatment.

1) Self-reported or carer-reported emergent OR worsening or pre-existing suicidality, subsequently confirmed by clinical interview.
2) Self-reported or carer-reported worsening of depression subsequently confirmed by clinical interview.
3) In addition to 1 AND 2, participant request and informed consent to restart best available treatment.

If a participant is considered clinically to be unable to withdraw from their current treatments or their condition significantly worsens during the screening and preparation phase to the point that the investigator considers that it would not be safe to randomise them, then they will be withdrawn from the trial. In this instance, the reason for withdrawal will be recorded and reported, as defined in the objectives. We will seek to replace participants who have been withdrawn prior to randomisation and will not consider them in the ITT analysis.

Trial Objectives and Design

Trial Purpose

The purpose of this trial is to evaluate the feasibility, safety and efficacy of psilocybin, given under supportive conditions, in a randomised, blinded trial design in adult participants with treatment resistant major depressive disorder.

Trial Objectives

Primary Objective

To evaluate the feasibility of a randomised, controlled trial design, in which a single dose of psilocybin 25mg PO vs placebo, is given to adult participants with treatment resistant major depressive disorder (TRD), under psychologically supportive conditions, with 6 weeks of follow up, by measuring recruitment rates, dropout rates and by estimating the variance of the primary outcome measure (MADRS) to inform upon the design of a phase 3 trial.

Secondary Objectives

The secondary objectives of this trial are as follows:

1) To assess the clinician-rated efficacy of psilocybin 25mg compared to placebo via:
   a) The change in the Montgomery Asberg Depression Rating Scale (MADRS) total score from Baseline to 3 weeks after treatment.
   b) The proportion of participants who demonstrate a response to treatment, where response to treatment is defined as a ≥50% decrease in MADRS total score from Baseline to Week 3. This will also be assessed at Weeks 1 and 6.
   c) The proportion of participants in remission, where remission is defined as a MADRS total score ≤10 at Week 3. This will also be assessed at Weeks 1 and 6.
2) To assess the participant-rated efficacy of psilocybin 25mg compared to placebo via:
   a) The proportion of participants who demonstrate a response to treatment, where response to treatment is defined as a ≥50% decrease in QIDS-SR-16 total score from Baseline (V2) to Week 3 (V6).
   b) The proportion of participants in remission, where remission is defined as a QIDS-SR-16 total score ≤5 at Week 3. This will also be assessed at Weeks 1 and 6.
3) To assess the participant-rated efficacy of psilocybin 25mg compared to placebo via:
   a) The change in the Maudsley Visual Analogue Scale (MVAS) total score and individual items from Baseline to 1 day (V4), 1 week (V5), 3 weeks (V6) and 6 weeks (V7).
4) To evaluate the safety and tolerability of psilocybin in participants with TRD based on adverse events (AEs), changes in vital signs and suicidal ideation/behaviour (measured using the Columbia Suicide Severity Rating Scale).

Exploratory Objectives

1) Explore the acceptability, tolerability and safety of delivering >1 dose of psilocybin by collecting safety data and participant narrative data from an optional 12 week open label extension to the trial.

2) Exploratory analyses of predictors and mediators of response to treatment

Trial Endpoints

Primary Endpoint

The primary endpoint in this study will be:

Recruitment rates, dropout rates and estimation of the variance of the primary outcome measure (MADRS).

Secondary Endpoints

The secondary endpoints in this study will be:

1) The change in the Montgomery-Asberg Depression Rating Scale total score from the Baseline Visit (V2 - 1 day prior to treatment) to Week 3 after treatment (V6).
2) The proportion of participants who demonstrate a response to treatment, where response to treatment is defined as a ≥50% decrease in MADRS total score from Baseline to Week 3.
3) The proportion of participants in remission, where remission is defined as a MADRS total score ≤10 at Week 3.
4) The proportion of participants who sustain a response up to Week 6, where response is defined as those with ≥50% decrease in MADRS total score on or before Week 3 and remaining at Week 6.

5) The change in the Maudsley Visual Analogue Scale (MVAS) total score and individual items from Baseline to 1 day (V4), 1 week (V5), 3 weeks (V6) and 6 weeks (V7).

6) Time to event measures: restart antidepressant medication for any reason, restart medication for continuing depressive symptoms or relapse from a previously recovered state (clinical judgement, supported by the QIDS-SR-16). Participants who withdraw from the study will be censored from the time to event analysis.

Exploratory Endpoints

This study will include the following exploratory endpoints:

1) Change from baseline (V2) in the following outcome measures
   a) The WSAS at week 6 (V7)
   b) The QIDS-SR-16 at week 3 (V6)
   c) The GAD-7 at week 3 (V6)

Trial Design

This study is a **two arm, double-blind, randomized, placebo controlled, between-subjects design.**

Trial Flowchart

The following flowchart, in contrast to Fig 2, depicts the main trial plus the open label extension.

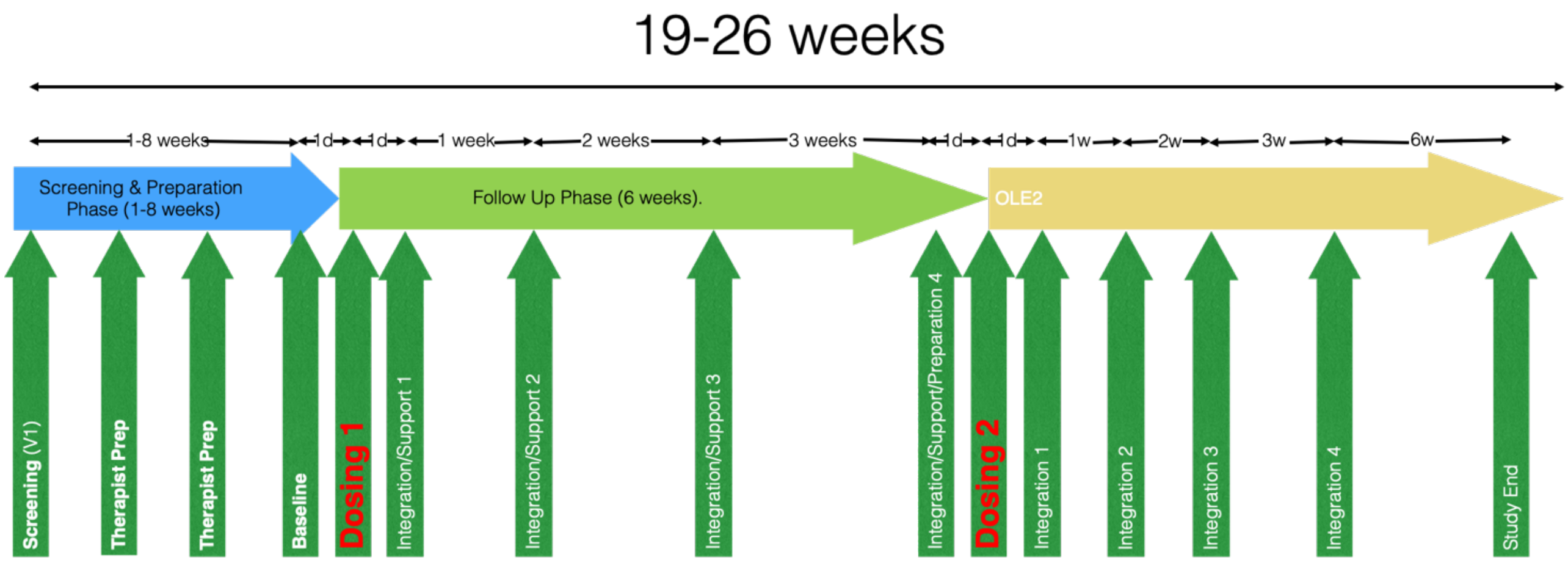

Fig. 4. Study Schematic including Optional Open Label Extension
Abbreviations: FtF, Face to Face; FU, Follow Up; V, Visit

Trial Medication

Investigational Medicinal Product (IMP)

Note: This information is substantially reproduced from the Investigator's Brochure written by Compass Pathways Ltd., and is reproduced here with permission. The Investigator's Brochure and any subsequent updates will be considered the reference document. The IMP Dossier is held by the MHRA with CESP number 986583. Because this contains commercially sensitive information, it is not available to the study team that have written this protocol.

Summary of IMP Basic Chemical Properties

**IMP Name:**	Psilocybin
**IUPAC Name:**	3-[2-(Dimethylamino)ethyl]-1*H*-indol-4-yl dihydrogen phosphate
**Other Common Name:**	4-Phosphoryloxy-*N*,*N*-dimethyltryptamine
**Structure:**	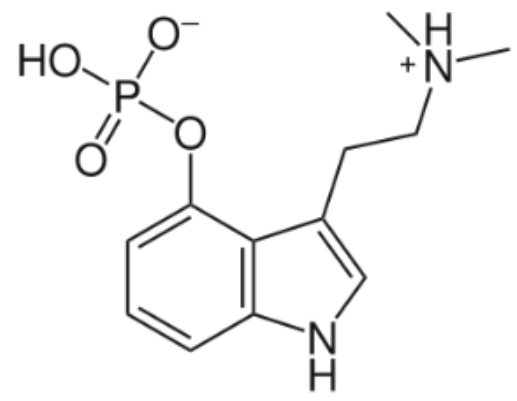
**Chemical Formula:**	$C_{12}H_{17}N_2O_4P$
**Molar mass:**	204.27 g/mol
**Melting point:**	173 to 176 °C (343 to 349 °F)
**Physical Form:**	Solid
**Soluble in:**	Water/saline

Table 1. IMP summary.

IMP Manufacture

Psilocybin is not commercially marketed anywhere in the world to our knowledge.

The drug substance, psilocybin, was manufactured by Onyx Scientific, Ltd. for Compass Pathways, Ltd. Juniper Pharma Services, Ltd. was responsible for the formulation of clinical supplies.

Psilocybin capsules are an immediate-release, solid dosage form in either hydroxypropylmethylcellulose (HPMC) capsules for oral administration. Psilocybin capsules contain a blend of 10% by weight Psilocybin Drug Substance and 90% by weight Starch 1500. Both strengths of Psilocybin capsules contain the same blend of Starch 1500 and Psilocybin Drug Substance, adjusted by fill weight to deliver the required product strength. Placebo capsules contain 50 mg Starch 1500 only. Both psilocybin and placebo capsules are No. 4 white HPMC capsules. Matching 5 mg psilocybin and placebo capsules were manufactured for this trial.

All capsules or tablets will be packaged in 30 mL high-density polyethylene (HDPE) bottles with a 35-mm tamper-evident twist off cap and a desiccant containing a single 5-capsule or 5-tablet dose:

- 25 mg treatment bottle: 5 × 5 mg capsules or 5 x 5 mg tablets

- Placebo: 5 x placebo capsules or 5 x placebo tablets

IMP Stability & Shelf Life

Please see the most up to date version of the investigator's brochure for information on stability and shelf life.

Dosing Regimen

Participants will be required to withdraw from existing antidepressant treatments prior to psilocybin treatment. This 'wash out' period will last a minimum of 1 week and a maximum of 8 weeks, depending on clinical need.

Participants will be asked to refrain from alcohol use the day before the treatment.

The treatment group (N=30) will receive a single dose of psilocybin 25mg given in 5 identical, opaque, HPMC capsules or tablets taken orally. The expected duration of action is 4-6 hours.

The comparison group (N=30) will receive a single dose of a placebo given in 5 identical, opaque, HPMC capsules or tablets taken orally. The expected duration of action is 4-6 hours, however it is not expected that the placebo will have any noticeable effects on the participant.

Participants will be asked to refrain from illicit drug use for the duration of their participation in the trial and asked to abstain from alcohol the day before the Dosing Visit (V3).

No other lifestyle changes will be required.

IMP Risks

Pharmacology

Psilocybin is defined chemically as 3-[2-(Dimethylamino)ethyl]-1H-indol-4-yl dihydrogen phosphate. It belongs to a class of drugs referred to as 'psychedelics' (derived from the Greek words for 'mind'/'soul' and 'manifesting'/'revealing') [18]. Psilocybin is detectable in plasma 20-40 min after oral administration of 0.224 mg/kg (10-20 mg total dose) [42]. The elimination half-life of psilocybin is 50 minutes [43]. Orally ingested psilocybin is dephosphorylated in the liver to psilocin, which is the pharmacologically active drug.

Psilocin is detectable in plasma 15-50 minutes after oral administration of 0.2 mg/kg psilocybin [24]. The half-life of psilocin ranges between 2 and 3 hours and it is detectable in plasma 6 hours after administration [42, 43]. Both psilocin (at 90-97%) and psilocybin (3-10%) are detectable in human urine, particularly conjugated with glucuronic acid, with the majority recovered from urine within 3 hours after oral administration and complete elimination occurring within 24 hours [44].

Psilocin is a partial agonist at a variety of 5-HT receptors, with activity at 5-HT2a thought to account for most of its subjective effects. Ray has published detailed receptor occupancy data for psilocin(20). Activity is restricted to the 5-HT receptors (except 5-HT3), D1, D3 and, to a much lesser extent, the serotonin transporter, alpha2 adrenoceptor family and the imidazoline1 receptor. Psilocin has no activity at the dopamine and norepinephrine transporters, alpha 1 adrenoceptors, muscarinic receptors, opioid receptors, histamine receptors or cannabinoid receptors. This observation is in-line with its benign physiological profile

in humans and animals, lack of dependence potential and safety in over-dosage, which we discuss and reference subsequently.

Reported Experiences with Psilocybin

Patient-reported experiences following administration of psilocybin in therapeutic settings include a variety of changes in affect, mood, perception and thought process. Whilst this was initially observed in studies performed prior to prohibition (pre-1970) [45-50], it has been repeated and confirmed by modern work since 1990 [51-56].

Observations within published literature generally have been subsumed under the Medical Dictionary for Regulatory Activities (MedDRA) categories of 'Nervous System Disorders' and 'Psychiatric Disorders' (see Table 1) although cardiac (increased BP), eye disorders (mydriasis), GI complaints (nausea) and nonspecific observations related to the therapeutic experience have been reported. The mechanism of action for psilocybin is consistent with these effects and they are expected within controlled clinical investigations. For the purposes of expedited safety reporting of Serious Adverse Reactions to Competent Authorities, observations listed in Table 1 should not be used as Reference Safety Information.

Reported-Observed Findings in the Literature Coded Using MedDRA

MedDRA SOC	MedDRA Preferred Term	Reported Term
Cardiac	Blood Pressure Abnormal	Blood Pressure – Instability
	Hypertension; Blood Pressure Increased	Blood Pressure Increased – Hypertension
	Hypotension; Blood Pressure Decreased	Blood Pressure Decreased – Hypotension
	Tachycardia	Heart Rate Increased
Eye Disorders	Mydriasis	Mydriasis
Gastrointestinal	Nausea	Nausea
	Vomiting	Vomiting
General Disorders and Administration Site Conditions	Asthenia	Weakness
	Fatigue	Tiredness
	Hyperreflexia	Reflexes Tendinae Increased
	Hyporeflexia	Reflexes Tendinae Decreased
	Sedation	Sleepiness/Sedation
	Yawning	Yawning
Nervous System Disorders	Dysmetria	Dysmetry
	Headache	Headache
	Tremor	Shaking Hands
		Tremor
Psychiatric Disorders	Aggression	Aggressive Increased
	Agitation	Stimulation
	Anxiety	Anxiety
		Anxiety/Fearfulness
	Crying	Tearing/Crying
	Depersonalization	Depersonalization
	Depressed Mood	Affect/Melancholy Increased Negative
		Depressed
	Derealization	Derealization
	Dissociation	Anxiety, Distance from Ordinary Reality
	Dizziness	Dizziness
	Dyskinesia	Motor Activity Spontaneous
	Dysphoria	Dysphoria

	Euphoric Mood	Joy-Intense Happiness
		Mood Positive
		Mood Positive Increased
	Feeling Abnormal	Dazed State
		Dreaminess
		Thought Disorders (Undefined)
	Feeling Jittery	Jittery
	Flat Affect	Psychosis-Like Symptoms Increased Negative (eg, Feeling Withdrawn/Apathetic)
	Hallucination	Hallucinations “Virtual”
	Hearing Perception Altered	Tone Estimation Impaired
	Mania	Affect/Mania Increased Positive
	Memory Impairment	Spatial Working Memory Impaired
	Mental Impairment	Mental Efficiency Reduced
		Thinking Less Clear
	Mood Altered	Affect Changes
		Peace/Harmony
	Psychotic Symptoms	Psychosis-Like Symptoms Increased Positive
	Social Avoidance Behavior	Introversion
	Somatic Symptom Disorder	Somatization
	Time Perception Altered	Long Interval Estimation Impaired
		Time Perception Altered – Objective
	Vision Distorted	Visual Alteration and Distortion

Table 2. Reported-Observed Findings in the Literature Coded Using MedDRA. This is **not** to be used as reference safety information for assessing whether an adverse event requires expedited reporting as Serious Adverse Reactions to Competent Authorities. Abbreviations: MedDRA, Medical Dictionary for Regulatory Activities; SOC, System Organ Class.

Psychological and Subjective Effects

Psilocybin produces an altered state of consciousness. By definition, this is multifaceted, however users have reported a shift towards introspection, changes in the experience of time, space, and self, intensification of emotion, and primary process modes of thinking and perceiving that can include misperceptions or illusions, an activation of latent and/or emotionally charged memories and associated symbolism and a blurring of the usual conceptual and self/other boundaries experienced in normal waking consciousness [6]. More prosaically, participants in our pilot trial described the experience as similar to dreaming, except that consciousness and awareness were relatively preserved [35]. Fifteen out of 20 patients reported anxiety at some point during the experience. Three out of 20 reported paranoia. In all cases these events were self-limiting and did not require pharmacological intervention (ibid.). Nonetheless, they reflect the multifaceted state, both positive and negative, that psilocybin can elicit. Our experience is that psilocybin is well tolerated and normal functioning is almost completely restored within 6-8 hours after oral drug administration [25, 57]. We have not observed ongoing symptomatology after 24 hours.

Walter Pahnke, in his late 1960s PhD thesis, found that psilocybin engendered spiritual or mystical experiences in divinity students during a religious service [58]. This study employed a placebo. In modern research, psilocybin was far more likely than methylphenidate to produce mystical or ‘peak’ experiences in people with existing religious or spiritual practices [53]. The core features of mystical experience are feelings of unity and interconnectedness with all people and things, a sense of sacredness, feelings of peace and joy, a sense of transcending normal time and space, ineffability, and an intuitive belief that the experience is a source of objective truth about the nature of reality.

Studerus et al. pooled raw data from 8 double-blind placebo-controlled experimental studies conducted between 1999 and 2008 with psilocybin [25]. They utilised the 5D-ASC questionnaire to characterise the state of consciousness elicited with psilocybin. The 5D-ASC is a visual analogue self-rating scale consisting of 94 items assessing 5 primary dimensions and 1 global dimension. The primary dimensions are comprised of several item clusters and described as:

- Oceanic boundlessness
- Dread of ego dissolution
- Visionary re-structuralisation
- Auditory alterations
- Vigilance reduction

Their analysis showed that psilocybin dose-dependently induces an altered state of consciousness which is characterised by marked changes in all mental functions, including perception, mood, volition, cognition and self-experience. The most prominent features described were alterations in visual perception, followed by positively and negatively experienced alterations of self-awareness and loosening of ego-boundaries. The changes in perception ranged from increased visual imagery with closed eyes, optical illusions, elementary hallucinations and synaesthesia to picture-like scenery hallucinations. The hallucinations were almost always recognised as such: loss of insight was rare. Auditory alterations were usually intensification of music and sounds or misperception of normal stimuli, as opposed to auditory hallucinosis per se. Reality testing usually remained intact, with most subjects sustaining a critical distance to their own subjective experience. Reduction of vigilance, manifested by sedation and a clouding of consciousness was also noted, which the authors considered a surprising finding in view of the popular conception that classic psychedelics fail to produce such effects. In any case, the authors explained these effects as being mild and attributed to the states of dreaminess, contemplativeness, and diminishment in attention consistent with the psilocybin-induced state (ibid.). In another work, the same group found that having a high score in the personality trait of Absorption, being in an emotionally excitable and active state immediately before drug intake, and having experienced few psychological problems in past weeks were most strongly associated with pleasant and mystical-type experiences, whereas high Emotional Excitability, low age, and an experimental setting involving positron emission tomography most strongly predicted unpleasant and/or anxious reactions to psilocybin(59).

Observers' descriptions of participants' affect and behaviour with psilocybin are generally consistent with participants' accounts, noting mood lability, changes in sense of self, primary process thinking and perceiving, as well as mystical experiences [51, 59, 60].

At this point it is salient to point out that the subject's 'perspective' on this broad spectrum of potential experiences is itself subject to a degree of variation. The mind 'set' of the individual as well as the 'setting' of the experience are important in mediating what is experienced and how it is appraised, both by the participant and the study team. Unpleasant experiences under psychedelics can be interpreted as part of a necessary psychotherapeutic process or as traumas in themselves, depending on the preconceptions of the observer. Griffiths et al. have found that despite the acute effects of psilocybin being distressing to some, the

same volunteers have retrospectively perceived the experience to be beneficial within the supportive context provided by the study team [60]. This may be salient because the supported abreaction and reprocessing of psychological trauma could be of benefit in treatment resistant depressive disorders, where subconscious processes of repression may be perpetuating the disorder. This observation also serves to disambiguate between the medically controlled use of psilocybin and the recreational use of magic mushrooms, where psychologically destabilising environments as well as poor preparation prior to ingestion can evidently lead to adverse outcomes. Still, in the 2018 Global Drug Survey, only 0.3% of all uses of psilocybin containing mushrooms resulted in the user seeking emergency medical assistance, the lowest of all drugs surveyed [61].

The occurrence and intensity of negative experiences in study settings such as in this trial can be reduced through informing participants about potential drug effects beforehand, establishing a rapport between the participants and the research team, attending to physical discomforts and providing emotional and practical support during the drug experience[9, 18].

Safety Data Collection

This study, as well as companion initiatives by Imperial College London and COMPASS Pathways Ltd., will examine patient self-reports across various domains mapped against observer and patient related changes in the severity of depression. The use of assessments such as the Positive and Negative Affect Schedule (PANAS), the 5D-ASC rating scale, the UKU side effects scale and the audio and video recording of psilocybin sessions immediately following psilocybin administration in the therapeutic setting, will capture a range of relevant behavioural symptomatology that will inform safety reporting.

Reference	Gouzoulis-Mayfrank et al. [54]	Griffiths et al. [53]	Hasler et al. [42]	Hollister and Sjoberg [62]	Passie et al. [24]	Wittmann et al. [63]
Reported and Observed Findings	U test, P<0.05<br>Increased positive psychosis-like symptoms (eg, hallucinations, unusual thoughts)<br>Increased negative psychosis-like symptoms (feeling withdrawn or apathetic)<br>Increased positive affect/mania<br>Increased negative affect/melancholy	Difference P<0.001<br>Anxiety, distance from ordinary reality<br>Dysphoria*<br>Joy / intense happiness<br>Peace / harmony<br>Positive mood<br>Somatisation*<br>Tearing / crying<br>Visual alteration and distortion<br>Yawning<br>P<0.01<br>Anxiety / fearfulness<br>Nausea P<0.05<br>Sleepiness / sedation<br>Spontaneous motor activity<br>Stimulation<br>Reduced mental efficiency**	Reported, not quantified.<br>Depersonalisation<br>Derealisation<br>"Virtual" hallucinations<br>Changes in affect<br>Thought disorders (undefined)	P<0.05<br>Increased aggressive<br>Depressed<br>Jittery<br>Less clear thinking<br>Reported, not quantified.<br>Dizziness<br>Nausea<br>Shaking hands<br>Weakness	AE, Percentage<br>Mydriasis 93%<br>Heart rate: accelerated 56%<br>slowed 13%<br>variable 31%<br>no change 0%<br>Arterial blood pressure: hypotension 34%<br>hypertension 28%<br>instability 22%,<br>no change 16%,<br>Nausea 44%<br>Reflexes tendineae: increased 80%,<br>decreased 6%<br>no change 13%<br>Dysmetry 16%,<br>Tremor 25%	Altered time perception – objective assessment<br>P<0.01<br>Impaired long interval estimation,<br>Impaired tone estimation (trend for 250 µg/kg)<br>Tiredness<br>Dazed state<br>Introversion<br>Dreaminess<br>Increased positive mood<br>Increased anxiety (250 µg/kg)<br>P<0.05<br>Impaired spatial working memory
Control Agent	Placebo	Methylphenidate	Placebo	Pre to Post-drug (2nd hour)	Percentage observed or Self-reported	Placebo
Number	n = 8	n = 30 (of 36)	n = 6	n = 24	n = 30	n = 12
Psilocybin Dose	0.2 mg/kg	30 mg/70 kg (~0.4 mg/kg)	0.224 mg/kg	225 µg/kg	8-12 mg IM & oral	115 µg/kg<br>250 µg/kg
Comparator	Between-subjects	Within-subjects	Within-subjects	Within-subjects	Not known	Within-subjects

Table 3. Acute Observed and Self-reported Effects and Side Effects of 0.114 – 0.4 mg/kg Orally Administered Psilocybin. *ARCI LSD scale loosely assesses dysphoria, jitteriness and somatic sensations. **ARCI BG is mental efficiency, separate from activation or feeling stimulated. Abbreviations: AE, adverse event; ARCI, Addiction Research Center Inventory; BG, Benzedrine group of the ARCI scale; IM, intramuscularly; LSD, lysergic acid dimethylamide.

Acute Adverse Events

From a physiological perspective, psilocybin is relatively benign. There is no evidence in the literature of end organ damage, even after significant over dose [10]

. This safety profile is similar for the other classical psychedelic, d-lysergic acid diethylamide (LSD), which shares similar receptor occupancy profiles [21]. Reversible mydriasis, mild elevations in systolic blood pressure and piloerection are most frequently observed [25, 52].

Adverse events to psilocybin appear to be mediated, at least in part, by variance in user's perspectives on both drug and symptom. Acutely observed and self-reported effects and side effects of psilocybin in modern research are summarised in Table 3. These effects may be perceived as pleasant by some and unnerving or distressing by others. Subjective effects vary within the same person from one episode of use to the next and are probably contingent on prior mental state and the environmental setting. Most of these effects are acute and last no longer than the 4-6-hour duration of drug effects when oral psilocybin is administered (ibid.).

Following high dose oral psilocybin (30 mg/70 kg), Griffiths et al. noted that 22% (8/36) of participants experienced a period of notable anxiety/dysphoria, sometimes including transient ideas of reference/paranoia [53]. No volunteer required pharmacological intervention and the psychological effects were readily managed with reassurance. This trial will use a maximal dose of 25mg (body weight independent).

Reports specific to consumption of psilocybin mushrooms report the occurrence of usually transient anxiety or psychosis-like symptoms[64-66]. The constellation of signs and symptoms exhibited are commonly subsumed under the label 'bad trip' and can encompass anything from agitation, dysphoria, anxiety or disorientation, to impaired concentration and judgment, rarely leading to reckless behaviour. A survey of 44 individuals admitted to hospital after 'magic mushroom' use reported the most common AE to be dysphoria, followed by nausea or vomiting in at least half of the admissions [65]. As might be expected after compounds known to alter perception, these individuals also reported perceptual and other somatic disturbances. In most cases, emergency department admissions related to anxiety or psychological distress after recreational 'magic mushrooms' use did not require ongoing hospitalisation [67]. The Global Drug Survey (2018) concluded that, from a variety of perspectives, psilocybin containing mushrooms were the least harmful recreationally used drug, with only 0.3% of usages resulting in attendance to emergency medical services [61].

The occurrence and intensity of anxiety or panic responses to psilocybin can be reduced through informing participants about potential drug effects beforehand and establishing a rapport between the participants and the research team(9). Supervision and monitoring of participants throughout the drug experience by personnel trained to deal with panic or anxiety by psychological means can also significantly limit adverse events. As a last resort, rescue medication (a benzodiazepine) effectively reverses the effect of psilocybin and treats associated anxiety, distress or paranoia.

Risky or Reckless Behaviour

As with other 5-HT psychedelics, people who have taken psilocybin in uncontrolled settings may engage in risky or reckless behaviour. There have been speculative and isolated reports of individuals jumping to their death from tall buildings or out of windows having consumed magic mushrooms in uncontrolled settings [68]

Participants in this clinical study will remain in a controlled and supported setting throughout the duration of the subjective effects of psilocybin and will remain under close supervision at the research facility until it is appropriate for their safe discharge. Thus, because of the level of supervision, it will be extremely unlikely that participants will perform any high-risk behaviours whilst under the influence of psilocybin and, if they do so, appropriate measures will be instigated to manage that risk. Both the Clinical Research Facility at King's College Hospital and the CMHRI host a wide variety of trials in mental health conditions and are thus experienced in managing such situations.

Delayed Adverse Events

In the immediate aftermath of a psilocybin dose, Studerus et al. reported that headaches, tiredness and exhaustion were the most commonly reported adverse events, but these were all relatively mild and failed to alter functioning in a discernible way [25]. More recently, researchers working with high doses of orally administered psilocybin in the US have noted the phenomenon of headaches in the 24 hour period (mean of 7 hours) post-administration of psilocybin in clinical research settings [69]. This is consistent with ours and other groups findings. Headaches or head pressure are reported in about 12.5-37.5% of participants in a dose-related manner [25, 36]. These findings are consistent with an investigation by Hollister, who measured the after effects of the administration of 36-205 µg/kg of psilocybin to 17 subjects in 27 separate trials and found occasional headaches as one of the most frequent complaints [70]. In Johnson et al.'s double-blinded study, a broad range of psilocybin doses (0, 5, 10, 20, and 30 mg/70 kg) were examined on headache in 18 healthy participants [69]. Most people suffered delayed headaches and the incidence, duration and severity of headaches were all increased in a dose-dependent manner. The authors speculated on the underlying biological mechanisms responsible and suggested the most likely was either delayed nitrous oxide release or the suppression of dorsal raphe nucleus inhibitory activity on the locus coeruleus with adrenoreceptor-linked vasoconstriction during the period of acute drug effects, followed by vasodilatation. We have found that headache caused by psilocybin responds to simple analgesia with paracetamol, which will be provided to participants on request.

In over 50 subjects dosed with 2 mg psilocybin IV, 45 of which took place in spatially restrictive and noisy brain scanners, anxiety scores were not significantly higher after psilocybin than placebo. Any reactions typically resolved spontaneously with supportive care, however 1 participant was given a single dose of midazolam for a period of paranoia that resolved after the acute effects of the drug had passed [57, 71, 72].

One of the subjects from the Studerus analysis following psilocybin administration did experience symptoms of emotional instability, anxiety and depression, which lasted several weeks and were severe enough for him to seek professional help. Further subjects described occasional mood swings, reactivation of old problems, excessive pensiveness and introversion, and memory and concentration problems in the first few weeks after the drug session. These were generally benign and, in all cases, resolved after a few weeks. However they underline the importance of adequate follow-up and appropriate psychological support after psilocybin

administration [25]. This study includes dedicated therapist and psychiatric support for each participant, and we will also provide participants with details of further support on request.

Prolonged Psychotic Reactions

In rare cases, prolonged psychotic states have occurred after use of psychedelics, including psilocybin-containing mushrooms [8, 73, 74]. However, in cases of recreational use of magic mushrooms, there is no pre-evaluation of psychological state or mental health prior to use, and thus drawing conclusions about aetiology is difficult. It may be that such individuals have an underlying liability to psychosis, unveiled by use of psychedelics. After examining the literature, Strassman concluded that hallucinogens (an archaic term for psychedelic), including psilocybin, might trigger psychotic episodes, but in a non-specific and non-aetiological manner, and that they were unlikely to be responsible for causing prolonged psychosis de novo [8]. These findings suggest that participating in a research study involving the administration of a psychedelic compound, such as psilocybin, is very unlikely to trigger transient or persistent psychosis – and it may be misleading to extrapolate from cases involving recreational use of magic mushrooms to controlled studies involving psilocybin given in medically and psychologically supportive settings.

Sidney Cohen's observational work in the 1960s (a survey covering data drawn from physician's reports of 25,000 administrations of psychedelics to a variety of healthy control subjects and patients in hospital settings) suggested that prolonged psychotic reactions and suicidality were no more frequent than might be expected by chance [7]. However, this study could be criticised for acquisition and recall bias and the threshold for determining these states is probably different in the modern era. The pilot trial research group at Imperial College London has dosed over 50 healthy volunteers with 2 mg IV psilocybin and conducted 40 dosing sessions in patients. They have not observed cases of prolonged psychosis. Similarly, in our healthy volunteer study of 89 participants (not yet published), we have not observed any incidences of prolonged psychosis. All the healthy volunteers had used psychedelics previously and were screened appropriately to exclude those with histories of psychosis. Studerus et al. found no incidences of prolonged psychotic reactions or precipitations of schizophrenia-spectrum disorders in the 110 subjects studied who received 1-4 oral doses of psilocybin (45-315 μg/kg body weight) [25]. High-dose studies are not associated with these reactions either [53], and none have been reported in modern studies or studies with patients [18].

Nonetheless, in contemporary studies participants are screened for past or present psychotic disorders and strong family histories of psychosis and these participants will be excluded from this study.

Hallucinogen Persisting Perception Disorder (HPPD)

Some people who have used 5-hydroxytryptaminergic psychedelics report persistent, distressing phenomena after the drug has left the body. These are mostly visual perceptual disturbances that can last from weeks to (in rare cases) years after use. This syndrome is defined as HPPD, and it has been reported after use of psilocybin-containing mushrooms [74, 75]. To date, there are no reports describing prevalence of HPPD in the general population, but an examination of previous reports and estimates of psychedelic use in the US suggests that functionally disabling HPPD is rare [73] and probably better explained by the presence of comorbid psychiatric disorders such as depression and anxiety than by HPPD itself. Halpern and Pope noted that many previous studies were affected by selection bias and confounding factors such as recent

drug intake, polydrug abuse, pre-existing psychiatric disorders and the clinician's variable perspectives on the drugs themselves. The two largest surveys on this phenomenon found that very few recreational psychedelic drug users (3-4%) report perceptual changes that are distressing to them [13]. There have been no cases of flashback-like symptoms occurring in modern clinical studies with psilocybin – even with high doses [53, 76]. The risk of HPPD occurring after psilocybin administration can be reduced by screening participants for potential risk factors such as substance dependence and through excluding people reporting HPPD after prior use of psychedelics [9]. We will specifically ask participants about the symptoms of HPPD during follow up, recording it according to the definitions outlined in Section 9.

Objective assessments of visual perception have detected changes in motion perception and binocular rivalry[56, 77]. A study of the effects of psilocybin on pre-pulse inhibition (PPI) reported enhanced PPI after a long interval between pre-pulse and pulse, and attenuated PPI when the interval was short [78]. In a later study, the 5-HT2a antagonist ketanserin (40 mg orally) was found to abolish the effect of psilocybin (260 μg/kg orally) on PPI as well as in controlled inhibition processes such as the Stroop test [79]. Psilocybin-induced changes were also normalised by risperidone (a mixed 5-HT2A/2C and D2 antagonist). Whereas an antagonist of the type 2 dopamine receptor (haloperidol) normalised only the euphoric symptoms, derealisation and depersonalisation yet had no effect on visual hallucinations and slightly potentiated the feeling of a loss of self-control [80].

Chronic Neurotoxicity & Personality Change

Whilst the possibility of chronic neuropsychological effects has never been specifically addressed with respect to psilocybin, earlier (uncontrolled) observational studies in chronic users of LSD [81] reported detecting subtle changes in personality or neuropsychological function, as described in a review by Halpern and Pope [73]. Such studies are likely to be confounded by other factors and did not describe significant functional impairment.

In contrast, there have been consistent anecdotal reports that sustained positive changes in personality, attitudes, and values, particularly in those subjects who have experienced profound personal insights and transcendent or mystical type experiences, have occurred following use of psychedelics. Studerus et al. found the highest percentages of subjects reporting positive changes in items measuring the attitude to altered state of consciousness (56%), the relationship to the environment/nature (38%), and aesthetic experiencing or appreciation of music and art (37%) [25].

Griffiths et al. found that participants reported an increase in positive attitude changes and behaviour two months after psilocybin administration. People who knew the participants well were questioned and agreed with the subjective reports [53]. The same group subsequently showed that those who fulfilled the criteria for mystical experience following high-dose psilocybin had increases in the personality domain of openness [82]. For these individuals, openness remained significantly higher than baseline more than 1 year after the session. Openness includes a relatively broad range of intercorrelated traits covering aesthetic appreciation and sensitivity, fantasy and imagination, awareness of feelings in self and others, and intellectual engagement. People with high levels of openness are considered permeable to new ideas and experiences and motivated to enlarge their experience into novel territory [83].

Recently, epidemiological studies have analysed the correlates and associations with psychedelic use. In two large population studies of adults in the US of 190,000 and 130,000, it was shown that classical psychedelic use (including psilocybin) was not linked to mental health problems, psychosis or suicidal behaviour, but associated with reduced psychological distress and suicidality [14, 15]. Hendricks has also found an association with reduced recidivism [84].

There is no evidence that psilocybin, particularly in the doses proposed for this study, has any deleterious toxic effects on non-brain organs. This is an observation shared by evidence with the other classical psychedelic, LSD. Massive overdoses of LSD have been survived without any objective long term sequelae [85]. The toxic ratio (ratio of the lethal dose to the commonly ingested dose) for psilocybin (and LSD) is estimated to be 1,000 or above [22]. Those case reports describing death or serious harm from ingestion of mushrooms have generally occurred due to mistaken consumption of poisonous varieties.

Abuse & Dependence Liability

Those studies examining use patterns in humans and studies in nonhuman primates (see Investigator's Brochure) suggest that psilocybin possesses little abuse potential. Surveys of drug use in the US do not ask specifically about use of psilocybin-containing mushrooms, suggesting that even if use of these mushrooms is relatively common, it is not of much official concern (despite the legal status of the drugs). Whilst very few studies specifically ask respondents about use of 'magic mushrooms', there appears to be little notable change in prevalence of "hallucinogen" use in adults aged 19 to 28, with 19.6% reporting some use in 2002 and 20% in 2003. There is no evidence that 'magic mushroom' use has been increasing in recent years, and may in fact be declining [68]. A survey of national drug use in Canada also indicated that hallucinogens accounted for very few admissions for substance abuse, a view supported in successive iterations of the Global Drug Survey [61].

Psychedelics are also not associated with compulsive drug seeking or physical withdrawal symptoms, which are the typical characteristics associated with dependence, underlined by the failure of attempts to train animals to self-administer psychedelics [86-88]. Therefore, there is little risk that exposing human volunteers to psilocybin will leave participants physically or psychologically dependent, except to the extent that it may be efficacious as a treatment for depression (which is a desired outcome). It is not expected that either psilocybin-naïve or experienced individuals will develop dependence after exposure. Studerus et al.s' results indicate that a carefully monitored administration of 1-4 doses of psilocybin to healthy volunteers within an experimental setting does not increase the risk for subsequent abuse of psilocybin or other illicit drugs [25]. Recreational use of 'magic mushrooms' tends to be occasional, e.g., monthly or yearly rather than weekly or daily [89]. Regular use of classical hallucinogens is very rare. Tolerance to the effects rapidly and completely develops after repeated daily doses and the drugs are not classically rewarding. Indeed, users often describe psychedelic experiences themselves as challenging rather than rewarding [68], even if the underlying goal may be one of seeking a degree of psychological equanimity. A convergence of the evidence suggests psilocybin to have a low potential for abuse and dependence, despite its legal classification.

Damage to Non-Brain Organs

Searches of publicly available databases of medical publications have failed to find any cases of psilocybin causing any serious pathology to any organ or system, a conclusion also reached by others [10]. More commonly, damage or disease to organs (illustrated by, for example, renal failure) is associated with mistakenly consuming poisonous mushrooms under the belief that they are psilocybin-containing varieties [90]. People in Mexico have likely used psilocybin-containing mushrooms for at least hundreds (and probably thousands) of years.

More formal clinical research with psilocybin began soon after its isolation and synthesis in 1958 [19]. A lack of case reports of physical illness or disease arising from exposure to psilocybin despite the long history of use within and outside of research settings is encouraging and indicative of association with few risks outside of those adverse events already described. The low rate of serious adverse events (SAEs) and lack of fatalities is especially notable in comparison to other commonly prescribed psychiatric medications and other recreationally used drugs [22].

Because of the sympathomimetic effect of psilocybin in causing mild elevations of blood pressure, there is a theoretical risk that administration may increase the risk of a cardiovascular or cerebrovascular event in those with pre-existing disease. For this reason, patients with a background history of severe cardiovascular disease or cerebrovascular disease will be excluded from this study.

Psilocin is an agonist at the 5-HT2b receptor, which may theoretically increase the risk of valvulopathy, a clinical phenomenon that was originally observed with chronic administration of fenfluramine [90]. There is no report in the literature of valvular heart disease in association with any classical psychedelic drug to our knowledge. We will, in any event, only be giving a single dose. The risk of valvulopathy would therefore appear negligible. Nonetheless, those with clinically determined severe cardiac disease will be excluded from this trial.

Psilocybin shows no activity at the human ether-a-go-go related gene potassium channel up to concentrations of 1mM (see Investigator's Brochure), suggesting the risk of ventricular tachyarrhythmias is negligible.

Fatalities

Verified fatalities that may be solely attributed to ingesting psilocybin or mushrooms containing only psilocybin and related compounds are very rare. Publications in the peer-reviewed scientific literature highlighting them are almost non-existent and, for those that are published, the causative aetiology is questionable. We are only aware of a single case report in the peer-reviewed literature of a death in which psilocybin containing mushrooms were directly implicated via toxicology analysis [91], and this was in an individual who had had a heart transplant and had consumed other psychoactive drugs. Whilst other reports in the non-peer reviewed literature have attributed death directly to the ingestion of psilocybin containing mushrooms, the reports are not peer-reviewed and a clear aetiological pathway is impossible to determine.

We have recently requested data from the UK's Office for National Statistics, which publishes data on deaths attributable to drug misuse on a yearly basis. The following table and line graph provides data on the numbers of deaths in which psilocybin or its relative, LSD, were cited in the death certificate.

Year	LSD	Psilocybin
1993	0	0
1994	1	1
1995	1	0
1996	0	0
1997	1	0
1998	1	0
1999	0	0
2000	0	0
2001	0	0
2002	0	0
2003	1	0
2004	0	0
2005	0	0
2006	0	0
2007	0	0
2008	0	0
2009	0	0
2010	0	0
2011	0	0
2012	0	0
2013	0	0
2014	0	0

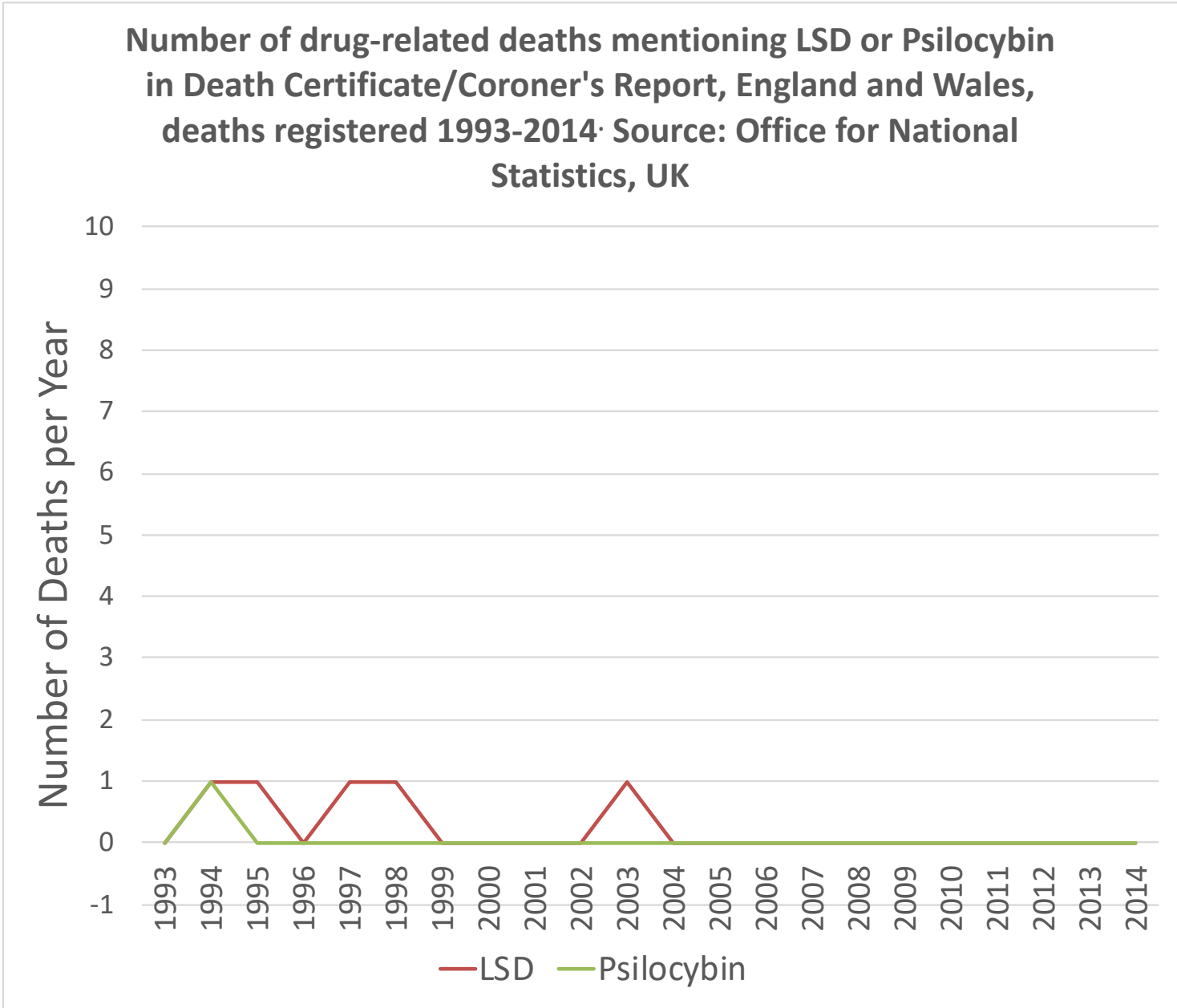

Table 3 & Fig. 4. Number of drug-related deaths mentioning LSD or Psilocybin, England and Wales, deaths registered 1993-2014[1,2,3]. 1. Cause of death was defined using the International Classification of Diseases, Ninth Revision (ICD-9) for the years 1993 to 2000 and Tenth Revision (ICD-10) from 2001 onwards. The ICD codes used to select deaths related to drug poisoning are shown in Box 1 on the Definition page. Deaths were included where the underlying cause was drug-related and the specified substance was mentioned on the death certificate. If both substances were mentioned, the death will be included in both columns. 2. Figures for England and Wales include deaths of non-residents. 3. Figures are for deaths registered, rather than deaths occurring in each calendar. Due to the length of time it takes to complete a coroner's inquest, it can take months or even years for a drug-related death to be registered. More details can be found in the 'deaths related to drug poisoning' statistical bulletin: www.ons.gov.uk/ons/rel/subnational-health3/deaths-related-to-drug-poisoning/index.html

These figures may represent an under-ascertainment as they may not include cases where psilocybin may have been a contributory factor.

Serotonin Syndrome

Extremely high doses of psilocybin may, in theory, induce symptoms of the serotonin syndrome, with some evidence of this using N,N-dimethyltryptamine in rats [92]. To our knowledge, no cases exist documenting serotonin syndrome in humans in response to psilocybin or psilocybin containing mushrooms. This is likely attributable to the fact that the doses of psilocybin that might provoke these physiological effects are likely to be many, many times greater than even the highest doses used in this research, other research, and recreational use of mushrooms, as well as psilocybin's relatively low affinity for the serotonin reuptake transporter [21].

Legal Position

Psilocybin is a Schedule I drug under the UN Convention on Drugs, defined as having no medical use and possessing high abuse liability and harm potential. This is reflected by UK law, where the Misuse of Drugs Act 1971 segregates drugs into 3 discrete classes (A, B and C) intended to broadly indicate the degree of risk associated; Class A being the most harmful and Class C the least harmful. Psilocybin is placed in the highest category (Class A) and attracts higher penalties for using and distributing compared to those drugs in other classes. In addition, in the UK, psilocybin is categorised under Schedule 1 in the Misuse of Drugs

Regulations 2001 and so thought to have no therapeutic value. This classification is questionable given that psilocybin has been classified as one of the least harmful recreational drugs of misuse by expert and users assessments [93] and there is no evidence of dependence.

This trial will be subject to the licensing requirements of that Schedule, which will be organised via the Sponsor and the UK Home Office. We confirm that all the requirements of that Schedule will be complied with.

Drug Accountability

Participants will receive the IMP in a controlled setting. Thus, we do not anticipate problems with drug accountability. Pursuant to requirements under Schedule 1, a strict audit trail of drug dispensing and usage will be kept. If a participant elects not to take the treatment on the Dosing Visit then the study team will return the dose to the Maudsley Hospital Pharmacy, who will arrange quarantine and/or secure destruction in accordance with regulations.

Storage of IMP

All capsules/tablets will be packaged in 30 mL high-density polyethylene (HDPE) bottles with a 35-mm tamper-evident twist off cap and a desiccant containing a single 5-capsule dose.

The shelf life will be extended in line with available stability data according to the defined shelf-life extension plan, assuming that there are no stability failures and no significant changes at any of the conditions.

Further information can be found in the Investigator's Brochure.

Subject Compliance.

The treatment is a single dose of psilocybin administered under the direct observation of the study team. No further compliance monitoring will be necessary.

Concomitant Medication

Participants will be withdrawn from antidepressant and antidepressant augmentation medications under the supervision of a psychiatrist prior to the Dosing Visit (V3). Withdrawal from antidepressants can induce troublesome insomnia and/or anxiety and/or an unpleasant (but not medically dangerous) withdrawal syndrome. We will allow the prescription of hypnotic medication (zopiclone, zolpidem or zaleplon within BNF dosage) for insomnia or a benzodiazepine (lorazepam or diazepam) or antihistamine (promethazine) for anxiety or withdrawal phenomena. Other over-the-counter remedies will be allowed, except for St John's Wort. This phase of the study is variable in duration to allow clinical flexibility. It is anticipated that a proportion of participants will not be able to tolerate withdrawal from their antidepressant medication due to deterioration in mood. Measurement of the proportion of participants that this occurs in is a necessary element of this feasibility trial. Those participants who cannot tolerate withdrawal will be withdrawn from the trial.

Participants will be allowed to continue to take medications for other medical illnesses throughout the study and on the Dosing Visit (V3), provided they do not interact unfavourably with the study treatment. More

specifically, drugs that are lipophilic and have antagonist activity at the 5-HT1a receptor may accentuate the effect of psilocybin by causing reciprocal stimulation of 5-HT2a. The two most commonly prescribed drugs (both beta-adrenoceptor antagonists) that participants may be on that exhibit these properties are as follows:

1. Propranolol

2. Pindolol

Participants on these drugs will be assessed for suitability for a suitable alternative, such as atenolol. Participant's medication and medical histories will be checked at screening (V1) and Baseline (V2), which is 1 day prior to the Dosing Visit (V3).

During the Dosing Visit (V3), rescue medication will be available for extreme agitation or distress that is unresponsive to non-pharmacological measures. We will use oromucosal midazolam 2.5-5mg given PO as a first line, with antipsychotics with 5HT2a antagonist activity available as 2nd line. This is compatible with NICE guidelines for rapid tranquilisation and developed for use with this trial in liaison with Professor David Taylor at the Maudsley Hospital Pharmacy Unit. Rescue medication will be given under the supervision of a medical doctor and participants will be monitored for as long as necessary after it has been given. Overnight hospital stay will be arranged if necessary.

During the follow up phase we anticipate that a proportion of participants will restart antidepressant treatment and we do not feel it would be ethical to discourage this. If participants do want to restart antidepressant treatment then this will be facilitated in liaison with the participant's primary and/or secondary care providers. Restarting of antidepressant medication will be recorded in the case record and the proportion may then be compared between groups.

End of Trial

The End of Trial is defined as the time point of database lock.

Selection of Subjects

Inclusion Criteria

- Age 25 - 80 years
- Fluent in the English language
- Fulfil Diagnostic and Statistical Manual of Mental Disorders (5th Edition) (DSM-5) criteria for a primary diagnosis of current single or recurrent episodes of MDD of at least moderate severity but without psychotic features as defined on the MINI 7.0. Positive and primary diagnoses on the MINI 7.0 will be subject to confirmation at clinical interview by a psychiatrist.
- 17-item HAM-D score ≥ 14.
- Have failed to respond to 2 or more antidepressants prescribed at the minimum effective dose for at least 6 weeks OR at least 1 antidepressant prescribed at the minimum effective dose for at least 6 weeks AND a course of evidence-based psychotherapy given for at least 6 sessions.
- For those aged ≥ 60 years, the first episode of depression must have started prior to their 60th birthday.

Exclusion Criteria

- Diagnosis of bipolar disorder (defined as meeting DSM-5 criteria for bipolar 1 or bipolar 2) on the MINI 7.0. Positive diagnoses on the MINI will be subject to confirmation at clinical interview by a psychiatrist.

- Diagnosis of psychotic disorder (defined as meeting DSM-5 criteria for any psychotic disorder) on the MINI 7.0, EXCEPT substance/medication induced psychotic disorder where the duration was limited to the acute period of direct intoxication with the substance/medication. Positive diagnoses on the MINI will be subject to confirmation at clinical interview by a psychiatrist.

- Diagnosis of drug or alcohol dependence syndrome (defined as meeting DSM-5 criteria for any dependence syndrome) on the MINI 7.0. Positive diagnoses on the MINI will be subject to confirmation at clinical interview by a psychiatrist.

- Diagnosis of any personality disorder (defined as meeting DSM-5 criteria for any personality disorder) based on clinical interview and the MINI 7.0. Positive diagnoses on the MINI will be subject to confirmation at clinical interview by a psychiatrist.

- Diagnosis of any dementia (defined as meeting DSM-5 criteria for any dementia disorder) based on clinical interview by a psychiatrist.

- Personal history of a ≥ 1 suicide attempt in the past year requiring hospitalization, defined using the CSSRS (Q6 (past year) = "y") and clinical interview with a psychiatrist.

- Other personal circumstances and behaviour judged to be incompatible with establishment of rapport or safe exposure to psilocybin.

- Depression secondary to other medical conditions

- Medical diagnosis incompatible with psilocybin treatment (see Section 6.2.1)
- Inability to provide a screening blood sample, urine sample or electrocardiogram.
- Biochemical abnormalities (defined as falling outside the normal reference range) as evaluated by a full blood count, full biochemistry profile and thyroid function tests. Biochemical abnormalities must also be determined as clinically significant by a medical doctor to fulfil the criterion for exclusion.
- Electrocardiographic abnormalities, defined as any abnormality that is not normal sinus rhythm and determined as clinically significant by a medical doctor.
- Women of child bearing potential not using adequate contraception (see Section 6.2.2).
- Pregnant or breast-feeding women.
- Those unable to give informed consent.
- Non-registration with a GP or failure to consent to sharing of the GP summary care record and any psychiatric assessments held.
- Those enrolled in another drug trial
- Hypersensitivity to the IMP or to any of the excipients or placebo

Exclusions for Pre-Existing Medical Conditions

Participants will be excluded if they have a current diagnosis of ≥1 of:

- Uncontrolled diabetes
- Hypertension (defined as a systolic blood pressure ≥ 160mm/Hg or a diastolic blood pressure ≥ 100mm/Hg on three separate readings). All readings of systolic blood pressure ≥ 140mm/Hg or diastolic blood pressure ≥ 90mm/Hg will be reviewed by a clinician. Hypertension ascertained prior to dosing will be subject to clinical confirmation via collateral information from the GP or other source.
- Cardiac failure, defined as class IV of the New York Heart Association classification
- Renal failure, defined as ≥ stage 4 (GFR ≤ 29mL/min)
- Liver failure, defined as a clinical diagnosis of liver fibrosis, cirrhosis of the liver, liver failure or advanced liver disease.
- Any cardiac arrhythmia, except atrial fibrillation.
- Any form of epilepsy

Past diagnosis of ≥1 of:

- Cerebrovascular accident or intracerebral trauma.
- Myocardial infarction within 1 year prior to the screening visit.

Women of Child Bearing Potential

A woman is considered of childbearing potential (WOCBP), i.e. fertile, following menarche and until becoming post-menopausal, unless permanently sterile. Permanent sterilisation methods include

hysterectomy, bilateral salpingectomy and bilateral oophorectomy. A postmenopausal state is defined as no menses for 12 months without an alternative medical cause. A high follicle stimulating hormone (FSH) level in the postmenopausal range may be used to confirm a post-menopausal state in women not using hormonal contraception or hormonal replacement therapy. However, in the absence of 12 months of amenorrhea, more than one FSH measurement is required. A man is considered fertile after puberty unless permanently sterile by bilateral orchidectomy.

For females of childbearing potential who may participate in the study, the following methods of contraception, if used properly and used for the duration of the study, are considered sufficient:

- Oral, patch or injection combined or progestestogel only contraceptives. Hormonal contraception must be associated with inhibition of ovulation
- Intrauterine device
- Surgical sterilization
- Vasectomized partner provided that partner is the sole sexual partner of the WOCBP trial participant and that the vasectomised partner has received medical assessment of the surgical success.
- Sexual abstinence. Sexual abstinence is considered a highly effective method only if defined as refraining from heterosexual intercourse during the entire period of risk associated with the study treatments. The reliability of sexual abstinence needs to be evaluated in relation to the duration of the clinical trial and the preferred and usual lifestyle of the subject.

Periodic abstinence, i.e., calendar, symptothermal, or post-ovulation methods are not an acceptable form of contraception for this study.

Male participants will be required to use condoms. Male participants not willing to follow the contraception advice will be excluded.

The duration of contraception required is defined as until the end of the study.

The investigator and each participant will determine the appropriate method of contraception for the participant during the participation in the study. This will be documented at Screening (V1).

Recruitment of Participants

Trial participants will be recruited by a variety of methods, including secondary care referrals, primary care referrals, referrals from the IAPT (Improving Access to Psychological Therapies) service, established registries of patients who have consented to research contact and directly via word of mouth or from advertising in the community or on social media.

Randomisation Procedure

The randomisation service will be provided by the King's Clinical Trials Unit (KCTU). Randomisation will use the web-based service hosted at the KCTU in accordance with a standard operating procedure and held on a secure server. The allocation sequence will be generated using minimisation, with sex (male, female) age group (25 to 59, and 60 plus) and past psilocybin use (yes, no) as factors. The sequence will be concealed

from all researchers and the trial statistician. A patient identification number from the web-based randomisation system will be provided after the patient has been both consented and randomised.

Implementation

Each participant will be assigned a unique study patient identification number (PIN) once they have given informed consent and entered the study. An off-site provider with the appropriate Schedule 1 manufacturing license (Fisher Clinical Services, Ltd.) will package and label the treatment before sending it by secure courier to the Maudsley Hospital Pharmacy, who will hold the treatment until the Dosing Visit. Allocation and blinding will be performed by the Maudsley Hospital Pharmacy staff using information provided by Fisher Clinical Services that defines whether placebo or intervention is in each bottle. On-call pharmacist staff will receive an email with details of treatment allocation to enable emergency unblinding out of hours. Strict allocation concealment will be maintained. On randomisation, email confirmations will be generated automatically and sent to other relevant staff without the details of treatment allocation.

Emergency Code Break

The Maudsley pharmacy will hold electronic and paper records of treatment allocation information for each participant. The blind may be broken in an emergency by contacting the Maudsley Pharmacy Hospital staff. A 24 hour on-call system is in place, allowing the blind to be broken at any time.

In the case of emergency, the blind can be broken by the investigator at any moment by making use of the emergency code break procedure as detailed in the protocol, above. The responsibility to break the treatment code in emergency situations resides solely with the investigator.

In the event of a SUSAR in which the KHP-CTO, sponsor or other regulatory authority require knowledge of treatment allocation, the King's CTU or Fisher Clinical Services will access the treatment allocation database and inform the relevant authority about treatment allocation without unblinding the study team.

Planned unblinding will occur after database lock and approval from the independent DMEC/TSC.

Withdrawal of Subjects

Participants have the right to withdraw from the study at any time for any reason. The investigator also has the right to withdraw patients from the study drug in the event of inter-current illness, AEs, SAE's, SUSAR's, protocol violations, cure, administrative reasons or other reasons. It is understood by all concerned that an excessive rate of withdrawals can render the study uninterpretable; therefore, unnecessary withdrawal of participants should be avoided. Should a participant decide to withdraw from the study, all efforts will be made to report the reason for withdrawal as thoroughly as possible and efforts will be made to continue to obtain follow-up data, if the participant consents to this.

Since the treatment consists of a single dose of psilocybin, it is not practical for participants to withdraw during the Dosing Visit.

Expected Duration of Trial

The trial is expected to begin recruitment in September 2020 and finish recruitment in September 2023. The total duration of a participant's involvement in the trial will vary between 7 and 26 weeks, dependent on the length of the (clinically flexible) screening & preparation phase and whether they consent to the open label extension. The follow up of participants will last for 6 weeks after the Dosing Visit (V3). The open-label extension will last up to 12 weeks. Participants who undertake both the main trial and open label extension may be involved with the study team for up to 26 weeks. Ongoing care for participants will be handed over to primary or secondary care teams after this time.

Trial Procedures

Trial Measures & Procedures

Trial measures and procedures are listed in Table 4, whilst a schedule of activities is listed in Table 5. Note that the times listed in Table 5 denote the estimated time taken for participant completion AND the estimated time taken for the study team to administer subsequently (data input, cleaning etc.).

List of Measures & Procedures

**Measure/Procedure**	**Type**	**Reference**	**Primary Rationale**	**Administration Schedule**
Informed Consent	Study Team-administered	Protocol	Screening	V1, [OLE1]
Medical History	Study Team-administered	Protocol	Screening	V1, V2, V3
Inclusion/Exclusion Criteria	Study Team-administered	Protocol	Screening	V1, V2, [OLE1]
Prior & Concomitant Medications	Study Team-administered	Protocol	Safety	V1, V2, V3, V4, V5, V6, V7, [OLE1, OLE2, OLE3, OLE4, OLE5, OLE6, OLE7]
Physical Examination	Study Team-administered	Protocol	Safety	V1, (V2, V3, V4, V5, V6, V7, [OLE1, OLE2, OLE3, OLE4, OLE5, OLE6, OLE7] if indicated)
Collateral History	Study Team-administered	Protocol	Safety	V1, V2, [OLE1]
Adverse Events (UKU/HPPDS)	Study Team-administered	Protocol & [94]	Safety	V1, V2, V3, V4, V5, V6, V7, [OLE2, OLE3, OLE4, OLE5, OLE6, OLE7]
Clinical laboratory investigations	Study Team-administered	Protocol	Screening	V1, V2, V4, [OLE3]
Electrocardiogram	Study Team-administered	Protocol	Screening	V1, V4, [OLE3]
Vital signs (BP/HR/Temp)	Study Team-administered	Protocol	Safety	V1, V2, V3 V4, [OLE1, OLE2, OLE3]
Weight	Study Team-administered	Protocol	Safety	V1, V2, V7
Urinalysis	Study Team-administered	Protocol	Screening	V1
Urine Drug Screen	Study Team-administered	Protocol	Screening	V1, [OLE1]
Serum/Urine Pregnancy Test (females only)	Study Team-administered	Protocol	Safety	V1, V2, [OLE1]
Additional biological samples (optional)	Study Team-administered	Protocol	Mechanism	V2, V4, V5, V6, V7
Neuroimaging (optional)	Study Team-administered	Protocol	Mechanism	V2, V5
Fitness watch/phone data	Study Team-administered	Protocol	Outcome	V7, [OLE 6]
Psychological Support & SI	Study Team-administered	Protocol	Support	V2, V3, V4, V5, V6, V7, [OLE1, OLE2, OLE3, OLE4, OLE5, OLE6, OLE7]
Treatment	Study Team-administered	Protocol	Intervention	V2, [OLE2]
MINI	Study Team-administered	[95]	Screening	V1
HAM-D-17	Study Team-administered	[96]	Screening	V1, V2
Maudsley Staging Method	Study Team-administered	[38]	Screening	V1
MADRS	Study Team-administered	[97]	Outcome	V2, V5, V6, V7, [OLE4, OLE5, OLE6, OLE7]
CSSRS	Study Team-administered	[98]	Safety	V1, V2, V4, V5, V6, V7, [OLE3, OLE4, OLE5, OLE6, OLE7]

YMRS	Study Team-administered	[99]	Safety	V2, V4, V5, V6, V7, [OLE3, OLE4, OLE5, OLE6,]
STAR-C	Study Team-administered	[100]	Mechanism	V2, V5, V6, V7, [OLE4, OLE5, OLE6]
QIDS-SR-16	Participant-administered	[101]	Outcome	V1, V2, V5, V6, V7, [OLE4, OLE5, OLE6, OLE7]
GAD-7	Participant-administered	[102]	Outcome	V2, V5, V6, V7, [OLE4, OLE5, OLE6, OLE7]
CTS	Participant-administered	[103]	Mechanism	V2
STAI	Participant-administered	[104]	Mechanism	V2, V5, V6, V7, [OLE4, OLE5, OLE6]
STAR-P	Participant-administered	[100]	Mechanism	V2, V5, V6, V7, [OLE4, OLE5, OLE6]
DESS	Participant-administered	[105]	Safety	V2
5D-ASC	Participant-administered	[106]	Mechanism	V4, [OLE3]
ASRM	Participant-administered	[107]	Safety	V2, V5, V6, V7, [OLE4, OLE5, OLE6, OLE7]
SCL-90	Participant-administered	[108]	Safety	V2, V5
DePAS	Participant-administered	Protocol	Safety	V5, V6, V7, [OLE4, OLE5, OLE6]
EQ-5D-5L	Participant-administered	[109]	Outcome	V2, V6, V7, [OLE5, OLE6, OLE7]
WSAS	Participant-administered	[110]	Outcome	V1, V2, V6, V7, [OLE5, OLE6, OLE7]
TAS	Participant-administered	[111]	Mechanism	V2, V7, [OLE6]
PANAS	Participant-administered	[112]	Safety	V2, V4, V5, V6, V7, [OLE3, OLE4, OLE5, OLE6, OLE7]
SCSS	Participant-administered	[113]	Mechanism	V2, V4, V7, [OLE3]
SCST	Participant-administered	Protocol	Mechanism	V2, V6, V7, [OLE5, OLE6]
CES	Participant-administered	Protocol	Mechanism	V4. [OLE3]
TSDI	Participant-administered	Protocol	Mechanism	V2, V7
WEMWBS	Participant-administered	[114]	Mechanism	V2, V6, V7, [OLE5, OLE6]
CNS	Participant-administered	[115]	Mechanism	V2, V6, V7, [OLE5, OLE6]
SAPAS	Participant-administered	[116]	Mechanism	V1, V2, V7, [OLE6]
MTI	Study Team-administered	[37]	Screening	V1
THINC-it	Participant-administered	[117]	Mechanism	V1, V2, V5, V7, [OLE4], [OLE6]
ETB	Participant-administered	[118]	Mechanism	V2, V5, V7, [OLE4, OLE6]
MVAS	Participant-administered	Protocol	Mechanism	V1, V2, V4, V5, V6, V7, [OLE3, OLE4, OLE5, OLE6]

1379 Table 4. List of study related procedures and measures. [X] Refers to procedures that will only be
1380 undertaken if the participant gives informed consent to the Open Label Extension part of the protocol.

1381 ## Schedule of Measures & Procedures

Study Team Administered Measures and Procedures													
**Visit**	**V1**	**V2**	**V3**	**V4**	**V5**	**V6**	**V7/ OLE1**	**OLE2**	**OLE3**	**OLE4**	**OLE5**	**OLE6**	**OLE 7**
**Day**	**-56 - -7**	**-1**	**0**	**1**	**7**	**21**	**41**	**42#**	**43**	**49**	**63**	**84**	**126**
**Week**	**-8 - -1**	**0**	**0**	**0**	**1**	**3**	**6**	**6#**	**6**	**7**	**9**	**12**	**18**
**Location**	**KCH Clinical Research Facility/CMHRI**												
**Window**	**N/A**	**-3**	**N/A**	**+1**	**+/- 1**	**+/- 2**	**+7/- 3**	**NA#**	**+/- 1 ****	**+/- 1 ****	**+/- 2 ****	**+/- 3 ****	**+/- 7 ****
**Length of Visit (hrs)***	**4**	**6**	**8**	**4**	**6**	**4**	**4**	**8**	**4**	**2**	**4**	**2**	**2 r**
Informed Consent	15						(15)						
Medical History	15	10	10										
Inc/Exc criteria	15	10					(15)						
Prior/concom. meds	15	10	5	5	5	5	5	5	5	5	5	5	5
Physical examination	10	10 ***	10 ***	10 ***	10 ***	10 ***	10 ***	10 ***	10 ***	10 ***	10 ***	10 ***	10 ***
Collateral	30	30					30						
AE/SAE	15	15	15	15	15	15	15	15	15	15	15	15	15
Clinical lab. blood tests	15	15		15					15				
ECG	15			15					15				
Vitals	5	5	5	5			(5)	5	5				
Weight	5	5					5					5	
Urinalysis	5												
Urine drug screen	5						(5)						
Urine/serum pregnancy test	5	5					(5)						
Bloods (additional)		15		15	15	15	15						
Neuroimaging		90			90								
Mobile phone/fitness watch							15					15	
Psych. support & SI (flexible schedule +/- remote)		60	360	60	60	60	60	360	60	60	60	60	60
IMP			15					15					
MINI	60												
HAM-D-17	10	10											
MSM	5												
MADRS		10			10	10	10			10	10	10	10
CSSRS	5	5		5	5	5	5		5	5	5	5	5
YMRS		5		5	5	5	5		5	5	5	5	
MTI*	10												
STAR-C		5			5	5	5			5	5	5	
**Participant Administered Measures and Procedures**													
QIDS-SR-16	5	5			5	5	5			5	5	5	5
GAD-7		5			5	5	5			5	5	5	5
CTS		5											
STAI		5			5	5	5			5	5	5	
STAR-P		5			5	5	5			5	5	5	
DESS		10											
5D-ASC				15					15				
ASRM		5			5	5	5			5	5	5	5
SCL-90		10			10								
DePAS					5	5	5			5	5	5	
EQ-5D-5L		5				5	5				5	5	5
WSAS	5	5				5	5				5	5	5
TAS		5					5					5	

PANAS		5		5	5	5	5		5	5	5	5	5
SCSS		5		5			5		5				
SCST		5				5	5				5	5	
CES				5					5				
TSDI		5					5						
WEMWBS		5				5	5				5	5	
CNS		5				5	5				5	5	
SAPAS	5	5					5					5	
THINC-it	20	20			20		20			20		20	
ETB		20			20		20			20		20	
MVAS	5	5		5	5	5	5		5	5	5	5	
(X) = If consent to OLE given	r = remote visit available				SI = Structured Interview				*joint completion with clinician				
	#up to 5 weeks after V7, if logistically necessary				***if clinically indicated				**relative to date of OLE2				
Medic Task	Nurse/RA Task				Therapist Task				Participant Task				

Table 5. List of Study-Team and Participant-Administered Activities and Measures. Numbers indicate estimated times for completion of procedures and associated administration by the study team. Hence the 'length of visit' is less than the 'total time'.

All study visits will take place at the NIHR/Wellcome Trust Clinical Research Facility at King's College Hospital, Denmark Hill, London, UK or at the Centre For Mental Health Research and Innovation (CMHRI), 5 Windsor Walk, Camberwell, London, .

Allowable Windows

Allowable windows for study visits are stated in Table 5, which are implemented to allow a degree of logistical flexibility for participants who may have other commitments and for the study team, who will be sharing facilities with other trials. This reflects 'real-world' clinical conditions and may improve retention and adherence to study protocols. The long allowable window at V7 is to allow for scheduled public holidays over which the university and trial facility is closed (Christmas and Easter) to be accommodated. OLE2 may take up to 5 weeks after OLE1, if necessary, for logistical flexibility. If this occurs, an additional check of eligibility, vital signs, pregnancy test (women only) and urine drug screen will be undertaken prior to dosing.

Description of Study Team Administered Measures

Informed Consent

Informed consent will be gained by a member of the study team at V1. Participants will be provided with a participant information leaflet to read prior to informed consent being sought. As much time as necessary will be given to address any questions or concerns from participants. Final eligibility and consent will be determined by a clinician.

Medical History

A medical history will be taken by a member of the study team at V1 in liaison with a clinician if necessary and reconfirmed at V2 and V3, informed by collateral information gained from the participant's general practitioner or secondary care team.

Inclusion/Exclusion Criteria

Inclusion and exclusion criteria will be determined by a member of the study team at V1 and informed by collateral information gained from the participant's general practitioner or secondary care team during the screening and preparation phase. Final confirmation will occur at V2.

Prior & Concomitant Medications

Prior and concomitant medications will be determined by a member of the study team in liaison with a clinician if necessary and informed by collateral information gained from the participant's general practitioner or secondary care team. This information will be gained at V1 and then updated at all subsequent visits.

Physical Examination

A physical examination must be conducted at screening to confirm participant eligibility and at any other moment if clinically indicated.

Adverse Events (UKU/HPPDS)

Adverse events will be determined by a member of the study team in liaison with a clinician if necessary. Adverse events will be collected from consent. Adverse events occurring at V1 will be recorded in the medical history. Adverse events occurring after V1 but before dosing will be recorded as adverse events but the causality will be documented as '1' (not related). We will use open questions to enquire how participants have been since last seen by the study team and then document adverse events as per Section 9.2.2. Subsequent to dosing, we will use a modified version of the UKU schedule of side effects to psychotropic drugs to standardise closed questions of AE data [94] with an expansion on neuropsychiatric side effects as specified by the following items: 1.11 General anxiety, 1.12 Panic attacks, 1.13 Derealisation, 1.14 Depersonalisation, 1.15 Flashbacks, 1.16 Obsessions, 1.17 Compulsions, 1.18 Elation, 1.19 Irritability, 1.20 Aggression, 1.21 Paranoia / suspiciousness, 1.22 Thought disorder, 1.23 Delusions, 1.24 Visual disturbances, 1.25 Visual hallucinations, 1.26 Auditory disturbances, 1.27 Auditory hallucinations, 1.28 Gustatory disturbances, 1.29 Gustatory hallucinations, 1.30 Olfactory disturbances, 1.31 Olfactory hallucinations, 1.32 Somatic disturbances, 1.33 Somatic hallucinations, 1.34 Loss of insight. Symptoms of hallucinogen persisting perception disorder (HPPD) will also be probed using an operationalised checklist of HPPD criteria derived from DSM5. Adverse event data will be collected at all visits, except that the UKU will be collected only once at V5 to ease participant burden and the HPPDS will not be collected at V4.

Clinical Laboratory Investigations

Routine clinical laboratory investigations will be taken at V1, V2 and V4. Bloods will be processed at KHP's onsite laboratories. Parameters evaluated will be: Haematology (Haemoglobin, Red blood cell count, Platelet count, white blood cell count and differential count), Clinical chemistry (Sodium, Potassium, Creatinine, Urea); Liver function test (Total bilirubin, Alanine Aminotransferase, Aspartate Aminotransferase, Alkaline phosphatase); T3, T4 and thyroid stimulating hormone. Results will be reviewed by a clinician.

Electrocardiogram

An electrocardiogram will be taken at V1 & V4. ECGs will be reviewed by a clinician.

Vital Signs (RR/SaO2/BP/HR/Temp)

Vital signs (respiratory rate, blood oxygen saturations on air, blood pressure, heart rate and temperature) will be taken at V1, V2, V3 & V4. Abnormal readings will be reviewed by a clinician in line with KHP policy.

Weight

Weight will be taken at V1, V2 and V7.

Urinalysis

Urinalysis will be taken at V1. Abnormal findings will be reviewed by a clinician.

Urine Drug Screen

A urine drug screen will be taken at V1. Positive findings will be reviewed by a clinician.

Serum/Urine Pregnancy Test (females only)

A serum pregnancy test will be taken at screening and a urine pregnancy test at V2 for pre-menopausal females only. For those pre-menopausal female participants choosing to take part in the open label extension, an additional urine pregnancy test will be undertaken at V7. Positive tests will be reviewed by a clinician and women confirmed to be pregnant will be withdrawn from the study.

Additional Bloods (optional)

An optional blood sample for processing and storage will be taken at V2, V4, V5, V6 and V7. Up to 50 mls of whole blood will be drawn.

Neuroimaging (optional)

Optional neuroimaging scans (MRI) will be offered at V2 and V5. Abnormal findings will be reviewed by a clinician. Each scan will last 1 hour.

Fitness Watch/Mobile Activity Data

If participants already have a fitness watch, or if they are collecting data via their mobile phone, or another device, then we will ask for their consent to share step counts per hour per day and sleep data (time in bed, time in light sleep, time in deep sleep) with us. They will be under no obligation to do so. The analysis of this data is not an intended outcome of this study. Data will only be collected from time points subsequent to the participant signing the ICF.

Psychological Support

Psychological support will be offered at V2, V3, V4, V5, V6 & V7. Additional psychological support will be available to participants on request. An element of flexibility in the schedule and provision of psychological support is necessary in a vulnerable population and trial such as this. As far as possible, participants will be supported by the same therapist throughout the trial. Participants will be able to access their therapist via a secure remote video or audio link if a face to face visit is not possible at the actual study visit, or if it is preferred by the participant.

The psychological model used in this trial will be similar to a broader model that has been developed and authorised separately for the Compass Pathways multicentre RCTs in depression. The centre delivering this trial is also a training centre for the delivery of therapists for the Compass multicentre RCTs (as well as being

a centre for the trial itself), therefore the therapist trainers in this trial have been trained as part of our existing trials and have unique and experience of working with this patient group in this setting. Training consists of didactic teaching, role play and, if feasible, practical experience in a clinical trial setting supervised by senior psychological therapists with extensive experience of delivering psilocybin therapy within trials performed both here at King's College London and at Johns Hopkins University Hospital in Maryland, USA. The therapist's training manual was reviewed and authorised separately as part of the NREC and MHRA's review of Compass Pathways' multicentre TRD trial with psilocybin.

The psychological model is divided into three phases that reflect preparation, dosing and integration respectively.

Preparation focusses on building mutual trust, active listening, psychoeducation about the drug experience and teaching participants methods of coping with difficult or unusual experiences that might arise. We will require participants to undergo a minimum of three hours of preparation, which may be delivered over a number of separate sessions. This will be assessed prior to dosing and the amount of preparation recorded in the case record form.

Dosing focusses on practical support, breathing and relaxation techniques, interpersonal grounding and fostering an attitude of non-judgemental acceptance and openness around any arising experiences.

Integration focusses on active listening and awareness of non-verbal cues to focus and maintain participant's attention on their current emotional state as it evolves. The purpose is to focus the participant on emotional and experiential reprocessing of material relevant to depression that may have arisen in the treatment session. In this sense, integration is generalisable to both treatment conditions (psilocybin or placebo), because relevant material may arise in either. The relational model (that is, the model of the relationship between therapist and participant) builds on acceptance and commitment therapy (ACT) and compassion focussed therapy (CFT). This allows a flexible approach that responds to evolving content and dynamics of each session. All therapists in this trial will have a primary qualification in a related discipline and will have attended a three-day training programme developed by our lead therapist trainers. This programme comprises didactic teaching, group work and role play. The first iteration of this programme was delivered in July 2019.

To further assess the psychological model, we may undertake a structured interview (SI) with participants before or after one or more of the psychological therapy sessions. This structured interview will be recorded and transcribed. We will collect objective data on the therapeutic relationship via the STAR-P and STAR-C scales as described in the schedule of assessments. The quantitative and qualitative analysis of this data is not an intended outcome in this protocol.

Treatment

The treatment (psilocybin or placebo) will be delivered at V3.

Mini Neuropsychiatric Inventory v7.0 (MINI).

The MINI was designed as a brief, structured interview for the major Axis I psychiatric disorders in DSM-5 and International Classification of Diseases, version 10. Validation and reliability studies have been

performed comparing the MINI to the Structured Clinical Interview for DSM-5 Patient Edition and the Composite International Diagnostic Interview (a structured interview developed by the World Health Organization). Version 7.0 of the MINI will be used for this study. The results of these studies show that the MINI has similar reliability and validity properties but can be administered in a much shorter period (mean 18.7 ± 11.6 min, median 15 min) than the above referenced instruments. It can be used by clinicians after a brief training session [95].

Relevant sections of the MINI will be administered at V1 for the purpose of confirming the diagnosis of MDD and ruling out psychiatric diagnoses that are exclusions for this trial. Diagnoses made using the MINI will be subject to clinical confirmation by a full psychiatric assessment.

17 Item Hamilton Depression Rating Scale (HAM-D-17)

The HAM-D-17 is a 17-item scale used to measure the degree of symptom severity in depressed patients [96].

The HAM-D-17 will be administered at V1 and V2. The total score from this assessment will be used as eligibility criteria at screening and baseline (minimum total score of 14, in keeping with other similar TRD trials such as STAR*D and our own LQD trial). The structure of the interview will be standardised with the Structured Interview Guide for the HAMD (SIGH-D).

The Maudsley Staging Method (MSM)

The MSM implements a 15 point scale that scores various aspects that define treatment resistance [38]. Participants will score at least 5/15 on this scale to be eligible for the trial, with no upper limit on the degree of treatment resistance.

The MSM will be administered at V1.

Montgomery Asberg Depression Rating Scale (MADRS)

The MADRS is a clinician-rated scale measuring depression severity designed to be sensitive to change in clinical status. It consists of 10 items, each scored from 0 (normal) to 6 (severe), for a total possible score of 60. Higher scores denote greater severity.

The MADRS will be administered at V2, V5, V6 and V7. The structure of the interview will be standardised through the use of The Structured Interview Guide for the MADRS (SIGMA) [119]. The MADRS interview will be performed either by a member of the study team who will be blind to treatment allocation and will not have attended the dosing session, or by a remote rater who is blind to treatment allocation and independent of the study team (funding dependent element). Regardless, before the interview commences, participants will be reminded not to discuss their beliefs about allocation with the interviewer and to concentrate purely on answers required for objective data collection.

The Columbia Suicide Severity Rating Scale (CSSRS)

The CSSRS is a clinician- and participant-rated scale that includes definitions of suicidal behaviour adapted from the Columbia Suicide History Form [98]. The definitions of ideation and behaviour were also used in the Columbia Classification Algorithm for Suicide Assessment, commissioned by the U.S. Food and Drug

Administration to classify retrospective reports of potentially suicidal adverse events and to provide interpretable data to inform pivotal drug safety questions.

The CSSRS will be administered at V1, V2, V4, V5, V6 and V7.

Young Mania Rating Scale (YMRS)

The YMRS is an eleven-item multiple choice diagnostic questionnaire used to measure the severity of manic episodes [99]. The purpose of including this scale is to provide an objective measure in the event of a participant developing mania, either incidentally or as an adverse reaction.

The YMRS will be administered at V2, V4, V5, V6, and V7.

Scale to Assess the Therapeutic Relationship (Clinician) (STAR-C)

The STAR-C (and its participant-rated companion, the STAR-P) is a 12-item measure assessing the therapeutic relationship between patient and clinician on three components: Collaboration, Positive Clinician Input, and Emotional Difficulties (clinician version)/Non-Supportive Clinician (patient version) [100]. The Collaboration subtest reflects a good rapport and a shared understanding of goals, mutual understanding, openness, and trust. Positive Clinician Input reflects the perception (by the participant) of the clinician to encourage, support, and listen to the participant. Emotional Difficulties/Non-Supportive Clinician Input reflect problems in the relationship. The range of total scores for both versions is 0–48, with a higher score suggesting better therapeutic relationships. Each version of the scale takes approximately 5 minutes to complete. Total scores and subscale totals can be obtained.

We have added two further questions to this scale for therapists to indicate whether they have personal experience of the psychedelic state and whether they are open about this experience with participants. These items are optional and are intended to inform upon an analysis of whether prior personal experience of the psychedelic state in the therapist and their openness about this with the participant is a salient variable in determining therapeutic outcome. This is based upon observations in pre-prohibition literature that prior personal experience of the altered state of consciousness elicited by classical psychedelics was a prerequisite to be a therapist utilising them.

The STAR-C will be administered at V2, V5, V6 and V7.

Description of Participant Administered Measures

Quick Inventory of Depressive Symptomatology (QIDS-SR-16)

The QIDS-SR-16 is a 16-item self-rated scale designed to assess depression severity over time on nine diagnostic symptom domains [101]. The total score ranges from 0 to 27 with 0 representing no depression and 27 representing severe depression. The total score is the sum of the 9 symptom domains. The QIDS-SR-16 will be collected at V1, V2, V5, V6 and V7.

Generalised Anxiety Disorder Scale (GAD-7)

The GAD-7 is a self-rated screening and symptom severity measure covering 7 of the most commonly occurring anxiety symptoms, which are often comorbid with depressive symptoms [102]. Participants choose

one of four severity scores and also indicate the degree to which these problems caused functional and/or social difficulties. Scores are derived from the values for each of the 7 domains.

The GAD-7 will be collected at V2, V5, V6 and V7. The GAD-7 takes less than 5 minutes to complete.

Childhood / Recent Traumatic Events Scale (CTS)

The CTS is a 7 item participant administered measure that collects data about childhood and recent traumatic life events and asks completers the extent to which the event was felt to be traumatic and the extent to which they confided in others about the event [103].

The CTS takes less than 5 minutes to complete and will be collected at V2 only.

State Trait Anxiety Inventory (STAI)

The STAI [104] is a 20 item likert scale designed to measure both state and trait facets of anxiety. It complements the GAD-7 by collecting data pertaining to longer term anxiety traits. Scores are derived from the state and trait question domains respectively.

The STAI takes less than 5 minutes to complete and will be collected at V2, V5, V6 and V7.

Schedule to Assess the Therapeutic Relationship (Participant) (STAR-P)

The STAR-P (and its clinician-rated companion, the STAR-C) is a 12-item measure assessing the therapeutic relationship between patient and clinician on three components: Collaboration, Positive Clinician Input, and Emotional Difficulties (clinician version)/Non-Supportive Clinician (patient version) [100]. The Collaboration subtest reflects a good rapport and a shared understanding of goals, mutual understanding, openness, and trust. Positive Clinician Input reflects the perception (by the participant) of the clinician to encourage, support, and listen to the participant. Emotional Difficulties/Non-Supportive Clinician Input reflect problems in the relationship. The range of total scores for both versions is 0–48, with a higher score suggesting better therapeutic relationships. Each version of the scale takes approximately 5 minutes to complete. Total scores and subscale totals can be obtained.

The STAR-P will be administered at V2, V5, V6 and V7. The STAR-P takes less than 5 minutes to complete.

Discontinuation Emergent Signs & Symptoms (DESS) Scale

The DESS is a scale designed to be sensitive to the signs and symptoms that develop on discontinuation of antidepressants, particularly those with monoaminergic reuptake inhibitor properties [105]. It is comprised of 43 items denoting various symptoms observed clinically in this scenario. Participants indicate whether each symptom is, since the last visit, a new symptom, an old symptom that has become worse, an old symptom that has improved, an old symptom that has not changed, or if the symptom is not present. The purpose of administering this scale is to objectively measure the degree and variability of antidepressant withdrawal symptoms in our study population, as this may predict drop out. The DESS will be collected at V2, only in participants who have been withdrawn from antidepressants. The DESS takes approximately 10 minutes to complete.

5 Dimension Altered States of Consciousness Questionnaire (5D-ASC)

The 5D-ASC is a 94 item self-report measure of the acute effects of psychoactive drugs using 5 primary dimensions. Participants are given a statement pertaining to an element of conscious experience then asked to mark a point on a line between ‘No, not more than usually’ and ‘Yes, much more than usually’. The 5 dimensions are ‘oceanic boundlessness’, ‘anxious ego dissolution’, ‘visionary restructuralization’, ‘auditory alterations’, and ‘reduction of vigilance’ [106, 120].

The 5D-ASC will be administered at V4. The 5D-ASC takes approximately 15 minutes to complete.

Altman Self-Rating Mania Scale (ASRM)

The ASRM is a 5-item self-report measure designed to probe the classical symptoms of mania with good test-retest reliability [107]. Participants are asked to choose one of four statements for each item.

The ASRM will be administered at V2, V5, V6 & V7. The ASRM takes less than 5 minutes to complete.

Symptom Check List (SCL-90)

The SCL-90 is a 90 item self-report measure [108], yielding nine scores along primary symptom dimensions and three scores among global distress indices. The primary symptom dimensions that are assessed are somatization, obsessive-compulsive, interpersonal sensitivity, depression, anxiety, hostility, phobic anxiety, paranoid ideation, psychoticism, and a category of "additional items" which helps clinicians assess other aspect of the participant’s symptoms (e.g. item 19, "poor appetite"). The three indices are global wellness index, hardiness, and symptom free. Numerous studies have been conducted demonstrating the reliability, validity, and utility of the instrument. It is one of the most widely used measures of psychological distress in clinical practice and research. The SCL-90 takes 12-15 minutes to complete and will be administered on V2 & V5.

Delayed Psychedelic After Effects Scale (DePAS)

The DePAS is an exploratory 20 item visual analogue scale measure designed for this trial that probes the presence of relative symptomatology across 10 domains that recreational users of psychedelics often report changes within. 2 statements per domain (that are phrased to be conceptually and phenomenologically antithetical) are rated on a 10cm visual analogue scale with 5cm referring to ‘about the same’, 0cm ‘much less’ and 10cm ‘much more’. The DePAS indirectly probes for symptoms of HPPD as well as both positive and negative experiences associated with psychedelic treatment. The DePAS takes less than 5 minutes to complete and will be collected on V4, V5, V6 and V7.

EuroQoL-5D-5L Scale (EQ-5D-5L)

The EQ-5D-5L was introduced by the EuroQoL Group in 1990. The EQ-5D-5L consists of a descriptive system and the EQ visual analogue scale with 5 levels (EQ VAS).

The descriptive system comprises five dimensions: mobility, self-care, usual activities, pain/discomfort and anxiety/depression. Each dimension has 5 levels: no problems, slight problems, moderate problems, severe problems and extreme problems. The participant is asked to indicate his/her health state by ticking the box next to the most appropriate statement in each of the five dimensions. This decision results in a 1-digit

number that expresses the level selected for that dimension. The digits for the five dimensions can be combined into a 5-digit number that describes the participant's health state.

The EQ VAS records the participant's self-rated health on a vertical VAS, where the endpoints are labelled 'The best imaginable health state' and 'The worst imaginable health state'. The VAS can be used as a quantitative measure of health outcome that reflect the participant's own judgement.

The EQ-5D-5L will be obtained at V2, V6, and V7. The EQ-5D-5L takes less than 5 minutes to complete.

The Work and Social Adjustment Scale (WSAS)

The WSAS is a validated and accepted scale to assess the degree of functional impairment that directly results from a condition [110] The scale consists of 5 questions covering different functional domains that participants rate on an 8 point scale. The WSAS will be collected at V1, V2, V6 & V7 and takes approximately 1 minute to complete.

Tellegen Absorption Scale (TAS)

The TAS is a 34-item scale that assesses imaginative involvement and the tendency towards becoming mentally absorbed in everyday activities, which may predict response to the IMP(115). Participants rate the 34 items as either 'true' or 'false'. The total score is the number of 'true' ratings.

The TAS will be collected V2 and V7. The TAS takes approximately 5 minutes to complete.

Positive and Negative Affect Scale (PANAS)

The PANAS is a 20-item measure comprising 2 mood scales that measure different facets of positive and negative affect respectively [112]. Each item is rated on a 5 point Likert scale that is summed to deduce an overall score reflecting the tendency to experience positive or negative affective states.

The PANAS will be collected at V2, V4, V5, V6 and V7. We will collect the PANAS twice on the dosing day, before and after treatment. The PANAS takes less than 5 minutes to complete.

Self Compassion Scale - State (SCSS)

The self compassion scale is 26 item scale comprising 6 subsections that tend to load together. Self-compassion entails being kind and understanding toward oneself in instances of pain or failure rather than being harshly self-critical; perceiving one's experiences as part of the larger human experience rather than seeing them as isolating; and holding painful thoughts and feelings in mindful awareness rather than over-identifying with them. Results from the development of the scale indicate that self- compassion is significantly correlated with positive mental health outcomes such as less depression and anxiety and greater life satisfaction, thus this scale may provide important information about psychological mechanism [113]. We have modified this scale to reflect the need to collect state information about self compassion. The SCSS will be collected at V2, V4 and V7 and takes about 5 minutes to complete.

Self Compassion Scale - Trait (SCST)

The self compassion scale - trait is 26 item scale comprising 6 subsections that tend to load together. Self-compassion entails being kind and understanding toward oneself in instances of pain or failure rather than being harshly self-critical; perceiving one's experiences as part of the larger human experience rather than

seeing them as isolating; and holding painful thoughts and feelings in mindful awareness rather than over-identifying with them. Results from the development of the scale indicate that self-compassion is significantly correlated with positive mental health outcomes such as less depression and anxiety and greater life satisfaction, thus this scale may provide important information about psychological mechanism [113]. The SCST will be collected at V2, V6 and V7 and takes about 5 minutes to complete.

Compassion Experience Scale (CES)

The CES is a 12-item likert scale developed in pilot form for this trial that explores the psychological theme of self-compassion experienced during treatment. Twelve items exploring elements of this are presented to participants, who are asked to indicate the degree of agreement on a line. These items are hypothesised to load onto 3 separate but related sub-themes: 1) self-kindness, 2) common humanity and 3) mindful experiencing. The scale is designed to capture data that may indicate whether experience of self-compassion is aetiologically relevant in understanding the putative mechanism of action of therapeutic change with psilocybin in depression. The CES will be collected at V4 only and takes less than 5 minutes to complete.

Trait Self-Description Inventory (TSDI)

The TSDI is a 50-item measure of the 'big five' personality traits [121]. Participants are provided with 50 statements and asked for their degree of agreement on a scale from 1 to 7 for stage 1 and 1 to 9 for stage 2.

The TSDI will be administered at V2 and V7. The TSDI takes approximately 10 minutes to complete.

Warwick Edinburgh Mental Wellbeing Scale (WEMBWS)

The WEMWBS is a 14 item inventory that measures mental wellbeing by asking participants to rate statements associated with different facets of wellbeing on a 5 point scale [114]. The scale has been validated for use in the UK population and takes less than 5 minutes to complete. The WEMBWS will be collected at V2, V6 and V7.

Connectedness to Nature Scale (CNS)

The CNS is a 14 item likert scale that measures facets of how connected raters feel to the natural world around them [115]. The scale has been validated for use in the UK populations and takes less than 5 minutes to complete. The CNS will be collected at V2, V6 and V7.

Short Assessment of Personality Scale (SAPAS)

The SAPAS is a very short assessment of personality [116] designed to capture indicative elements of personality that may further indicate personality structures captured within the ICD-10 and DSM-IV constructs of personality disorder. Eight items are rated 'yes' or 'no'. We will collect the SAPAS at screening in order to assess whether answers to any particular question are indicative of positive or negative response to psilocybin. The SAPAS will be collected at V1, V2 and V7 and takes 1 minute to complete.

Maudsley Treatment Inventory (MTI)

The MTI is a self-administered list of antidepressant and antidepressant augmentation treatments as defined by NICE Guidelines, the British National Formulary and the Maudsley Prescribing Guidelines (April 2018)

along with space for dose, duration, tolerability, adherence and response [37]. It is a natural companion to the Maudsley Staging Method in classifying the degree of treatment resistance.

The MTI will be administered at V1 and takes approximately 10 minutes to complete.

THINC-it Cognitive Battery

THINC-it is a freely available, participant-administered, computerized tool integrating subjective and objective measures of cognitive function salient in adults with major depressive disorder (MDD). It is delivered as a computer program with simple instructions.

The THINC-it battery will be administered at V1, V2, V5 and V7 and takes approximately 10 minutes to complete.

Emotional Test Battery (ETB)

An ETB comprises a freely-available, participant-administered, computerized battery of tests for different facets of emotional processing. For example, the facial expression recognition test features six basic emotions (happiness, surprise, sadness, fear, anger and disgust) taken from the Pictures of Affect Series. The facial stimuli are presented on a computer screen (random order) for 500 msec and then replaced by a blank screen. Participants make their responses by pressing a labeled key on a computer keyboard. Each participant is asked to respond as quickly and as accurately as possible. In the emotional categorization task, sixty personality characteristics selected to be extremely disagreeable (e.g., domineering, untidy, hostile) or agreeable (cheerful, honest, optimistic) are presented on the computer screen for 500 msec. These words are matched in terms of word length and ratings of frequency and meaningfulness. Participants are asked to categorize these personality traits as likable or dislikable as quickly and as accurately as possible. In the emotional memory task, immediately after completion of the emotional categorization task, participants are asked to recall as many of the personality traits as possible. This task therefore allows the assessment of incidental memory for positive and negative characteristics.

An ETB will be administered at V2, V5 and V7 and takes approximately 20 minutes to complete.

Maudsley Visual Analogue Scales (MVAS)

The Maudsley Visual Analogue scales indicate the quality of mood, experience of pleasure and experience of suicidal thoughts or feelings during a preceding interval of time that can be varied according to study need. These three items have been developed specifically to measure the three core symptoms that patients with TRD report most frequently. The MVAS may be administered at V1, V2, V3, V4, V5, V6 and V7 and will take less than 1 minute to complete.

Expectancy and Blinding VAS Measures

As part of the Visual Analogue Scales we will probe elements related to blinding adequacy and expectancy effects at V1, V2, V4, V6 and V7.

Open Label Extension (OLE)

Participants who complete follow up at 6 weeks (V7) will be offered an optional 12-week OLE to the trial, as previously described and discussed in Section 3.3.13.

The schedule of assessments and procedures is detailed in Table 5. All study related procedures in the OLE are equivalent to those in the main clinical trial, with no additional separate tests or procedures that have not already been described. Analysis of data from the OLE will be exploratory in nature only, however adverse event data will be reported as per the criteria in Section 9.

Protocol Deviations

All protocol deviations will be assessed and documented on a case-by-case basis before database lock, and deviations considered having a serious impact on the efficacy results will lead to the relevant participant being excluded from the analysis.

Assessment of Efficacy

Assessment of efficacy is not a primary objective in this trial.

Primary Efficacy Parameters

1) The change in the Montgomery Asberg Depression Rating scale (MADRS) measured from Baseline (V2) to 3 weeks after Treatment (V6).

Secondary Efficacy Parameters

1) The Montgomery Asberg Depression Rating scale (MADRS), used to determine:
   a) The proportion of participants who demonstrate a response to treatment, where response to treatment is defined as a ≥50% decrease in MADRS total score from Baseline to Week 3. This will also be assessed at Weeks 1 and 6.

   b) The proportion of participants in remission, where remission is defined as a MADRS total score ≤10 at Week 3. This will also be assessed at Weeks 1 and 6.

   c) The proportion of participants who sustain a response up to Week 6, where response is defined as those with ≥50% decrease in MADRS total score on or before Week 3 and remaining at Week 6.

2) The Quick Inventory of Depressive Symptoms (QIDS-SR-16), used to determine:

   a) The proportion of participants who demonstrate a response to treatment, where response to treatment is defined as a ≥50% decrease in QIDS-SR-16 total score from Baseline (V2) to Week 3 (V6).

   b) The proportion of participants in remission, where remission is defined as a QIDS-SR-16 total score ≤5 at Week 3. This will also be assessed at Weeks 1 and 6.

   c) The proportion of participants who sustain a response up to Week 6, where response is defined as those with ≥50% decrease in QIDS-SR-16 total score on or before Week 3 and remaining at Week 6.

3) The change in the Maudsley Visual Analogue Scale (MVAS) measured from Baseline (V2) to 1 day (V4), 1 week (V5), 3 weeks (V6) and 6 weeks (V7).

Assessment of Safety

Specification, Timing and Recording of Safety Parameters.

Patients with treatment resistant depression are considered a vulnerable population and therefore we will ensure monitoring and safety during the trial, as per GCP guidelines. Whilst the IMP is a Schedule 1, Class A substance, and therefore legally defined as having a high potential for harm and dependence, the summation of evidence reported here does not support this designation and we will be giving a single dose of the IMP only. We anticipate, therefore, that the risks associated with the IMP itself will be relatively low, however we comprehensively review the evidence for this in the Investigator's Brochure, and above in Section 5.3.

We do, however, consider that the risks associated with clinical depression itself, the preparations to receive the IMP, and with the consequences of randomisation, may be more pronounced than the risks of the IMP. These can be summarised under the risks consequent on the need to withdraw participants from their antidepressants prior to randomisation and the risk of further deterioration in mental state during the trial.

In the pilot trial we found that participants for the study were content to discontinue their current antidepressant medications because, by definition, they were inefficacious +/- had burdensome side effects. There was no instance in which a participant, as a consequence of medication withdrawal, demonstrated a deterioration in mental state severe enough to warrant clinical concern about risk to self or others. However, this may in part have been because all participants knew that they would be receiving the full dose of the IMP, whereas in this study participants will be aware that they will be randomised. This has two potential effects:

1) Affecting recruitment
2) Affecting a participant's mental state during and after the Dosing Visit (V3)

Assessing these parameters is a necessary part of the measurement of feasibility in this study, so we will record drop outs and reasons in the CRF and we will also arrange for participants to restart antidepressant therapy in liaison with their primary or secondary care teams and/or refer them for psychological therapy if their mental state worsens. One participant dropped out immediately after receiving psilocybin in the pilot trial, citing a lack of efficacy and refusing to engage in further follow up. This participant did subsequently provide follow up data six months later.

The screening process will exclude participants who, because of their prior history, may react more unfavourably to the IMP. A list of exclusion criteria is contained in Section 6.2. Medical comorbidities will be assessed by a medical history, physical examination, routine clinical laboratory investigations and an electrocardiogram. Final eligibility will be determined by an experienced clinician. The medical history, physical examination, clinical laboratory investigations and an electrocardiogram are performed with the primary purpose of excluding participants with medical conditions that may be incompatible with treatment with psilocybin, rather than because of concerns about the toxicity of the IMP itself. For example, it would be inappropriate to include a participant with uncontrolled diabetes in the trial, because the effect of the drug may include appetite suppression, which may adversely affect glucose levels. Further physical examination

and/or clinical laboratory investigations may be initiated by the study clinician at their discretion at any point during the follow up period, and would be particularly appropriate if, for example, an adverse event is reported where the aetiology may be clarified by further such investigation.

The SP phase will, for a proportion of participants, involve stopping established antidepressant regimens. Participants will be supported by a psychiatrist and the study team during this period. Discontinuation syndrome signs and symptoms will be captured with the DESS scale. Drop outs due to intolerance of antidepressant withdrawal and/or worsening of mental state will be recorded and reported in line with this protocol. We have specified a variable period for the SP phase, particularly in view of the need for clinical flexibility during this time. We will allow prescription of symptomatic medications as described above to manage the discontinuation syndrome and the insomnia that can result from withdrawal of antidepressants. However, participants will need to have refrained from using these (aside from short acting hypnotics) for 24 hours prior to the morning of the Dosing Visit (V3).

We will specifically enquire about adverse events at each of the follow up visits. Adverse events will be enquired about with open ended questions initially, however we will also use the UKU schedule for side effects to psychotropics to structure collection of data with closed questions enquiring for specific adverse events. Adverse events of special interest are further described below. All adverse events will be recorded.

Participants will be asked to contact the study team about any adverse events they experience in-between face to face visits. A member of the team will speak to the patient and trial clinicians will advise and assess the patient's health and wellbeing. Adverse events will be reported as outlined in Section 9.2.

Safety data will be made available to the DMEC and TSC, who will then advise the Sponsor whether the trial should continue or be terminated on the basis of the data provided.

Procedures for Recording and Reporting Adverse Events

We will follow the relevant definitions provided by The Medicines for Human Use (Clinical Trials) Regulations 2004 and Amended Regulations 2006 with regards to adverse events:

Adverse Event (AE): Any untoward medical occurrence in a subject to whom a medicinal product has been administered including occurrences which are not necessarily caused by or related to that product.

Adverse Reaction (AR): Any untoward and unintended response in a subject to an investigational medicinal product which is related to any dose administered to that subject.

Unexpected Adverse Reaction (UAR): An adverse reaction the nature and severity of which is not consistent with the information about the medicinal product in question set out in the Investigator's Brochure

Serious Adverse Event (SAE), Serious Adverse Reaction (SAR) or **(Suspected) Unexpected Serious Adverse Reaction ((S)USAR)**: Any adverse event, adverse reaction or unexpected adverse reaction, respectively, that

- Results in death;
- Is life-threatening;

- Required hospitalisation or prolongation of existing hospitalisation;
- Results in persistent or significant disability or incapacity;
- Consists of a congenital anomaly or birth defect.

Although not a serious adverse event, any unplanned pregnancy will also be reported via the SAE reporting system.

Reporting Responsibilities

King's College London and South London and Maudsley NHS Foundation Trust (the co-sponsors) have delegated the delivery of the Sponsor's responsibility for Pharmacovigilance (as defined in Regulation 5 of the Medicines for Human Use (Clinical Trials) Regulations 2004 to the King's Health Partners Clinical Trials Office (KHP-CTO).

All SAEs, SARs and SUSARs will be reported immediately (and certainly no later than 24 hours) by the Investigator to the Chief Investigator who will review and report to the KHP-CTO in accordance with the current Pharmacovigilance Policy.

The KHP-CTO will report SUSARs to the relevant ethics committee and to the regulatory authorities (MHRA, competent authorities of other EEA (European Economic Area) states in which the trial is taking place.

Reporting timelines are as follows:

- SUSARs which are fatal or life-threatening must be reported not later than 7 days after the sponsor is first aware of the reaction. Any additional relevant information must be reported within a further 8 days.
- SUSARs that are not fatal or life-threatening must be reported within 15 days of the sponsor first becoming aware of the reaction.
- The Chief Investigator and KHP-CTO (on behalf of the co-sponsors), will submit a Development Safety Update Report (DSUR) relating to this trial IMP, to the MHRA and REC annually.

Documentation, Classification & Follow Up of Adverse Events

AEs occurring after the participant signs the consent form until the last study visit will be recorded. AEs that occur prior to the Dosing Visit (V3) and after the screening visit (V1) will be recorded in the adverse event log, with the causality set to '1' (not related). We will not consider diseases or conditions present before the treatment period to be AEs unless they worsen after the Dosing Visit (V3). All significant illnesses in the 3 months prior to the screening visit (V1) as well as all illnesses ongoing at the screening visit (V1) should be recorded in the case record form.

All clinical laboratory results, vital signs, and ECG results or findings will appraised by the investigator to determine their clinical significance. Isolated abnormal clinical laboratory test results, vital sign findings, or ECG findings (i.e., not part of a reported diagnosis) should be reported as AEs if they are symptomatic, require corrective treatment, or constitute an AE in the investigator's clinical judgment.

At each time point, the investigator will determine whether any AEs have occurred by evaluating the participant. AEs may be directly observed, reported spontaneously by the participant or by questioning the

participant at each time point. The investigator must assess all AEs to determine intensity, causality and seriousness, in accordance with the definitions in Sections 9.1 and 9.2. The investigator's assessment must be clearly documented in the study site's source documentation. Diagnosis should, in preference, be specified as the AE or SAE term. If a diagnosis is unavailable, then the primary sign or symptoms should be reported with additional narrative details.

Each AE will be classified according to the following criteria:

- Mild. The AE does not interfere in a significant manner with the participant's normal level of functioning.
- Moderate. The AE produces some impairment of functioning but is not hazardous to the participant's health.
- Severe. The AE produces significant impairment of functioning or incapacitation and is a definite hazard to the participant's health.

Severity versus Seriousness: Severity is used to describe the intensity of a specific event. However, the event itself may be of relatively minor medical significance (such as severe headache), and therefore not serious, which reflects the overall impact on the participant.

When changes in the intensity of an AE occur more frequently than once a day, the maximum intensity for the experience should be noted. If the intensity category changes over several days, those changes should be recorded separately (with distinct onset dates).

Each AE will be assessed as to its relationship to the IMP, based on the following criteria. Although the attribution by the investigator will be collected for reported events, for analytic purposes a temporal association with the use of the IMP will be assumed sufficient for at least plausible association.

- Not related. No causal relationship exists between the IMP and the AE, but an obvious alternative cause exists, for example the participant's underlying medical condition or concomitant therapy.
- Possibly related. A connection with the administration of the IMP appears unlikely but cannot be ruled out with certainty. An AE may be considered possibly related if or when it meets 2 of the following criteria:
    - (1) it follows a reasonable temporal sequence from administration of the IMP;
    - (2) it could not readily have been produced by the participant's clinical state, environmental or toxic factors, or other modes of therapy administered to the participant; or
    - (3) it follows a known pattern of response to the IMP.
- Related. It is reasonable and plausible that the AE has been caused by the IMP.

The relationship of the AE to the IMP will be further considered according to the following criteria:

- Known class effect
- Biological plausibility
- Lack of alternative explanation

The **action** taken regarding the IMP in relation to the AE can generally be divided into two categories

1. Dose not changed. No change in the study drug dosage was made.

2. Not applicable. Participant died, study treatment had been completed prior to reaction/event, or reaction/event occurred prior to start of treatment

Other actions taken for an AE may be classified as follows

1. None. No treatment was required.
2. Medication required. Prescription and/or over-the-counter medication was required to treat the AE.
3. Hospitalisation or prolongation of hospitalisation required. That is, hospitalisation was required or prolonged because of the AE, whether medication was required or not.
4. Other

The **outcome** of an AE may be classified thus:

1. Recovered/resolved. That is, the participant fully recovered from the AE with no residual effect observed.
2. Recovering/resolving. That is, the AE improved but has not fully resolved.
3. Not recovered/not resolved. That is, the AE itself is still present and observable.
4. Recovered/resolved with sequelae. That is, the residual effects of the AE are still present and observable, including sequelae/residual effects.
5. Fatal. That is, when death is a direct outcome of the AE.
6. Unknown

All AEs will be followed up until resolved or stable. The outcome will be documented on the CRF.

Other Procedures for Reporting and Recording Adverse Events

Due to the limited safety information available for psilocybin, in addition to reporting all adverse events in line with the requirements of this protocol, the study team will be recording adverse events separately. These AEs will be transferred to ICON for adding into a safety database, managed by both ICON and COMPASS Pathways, allowing for the continued development of safety data around psilocybin. Patients will be asked to consent for this aspect of the research; patients who do not consent will not be excluded from study participation.

For the purposes of the safety database maintained by Compass Pathways and ICON, they will be informed of the unblinding results at the end of the trial (the time point of database lock).

Adverse Events of Special Interest (AESI)

An adverse event of special interest (AESI) is an AE (serious or non-serious) of scientific and medical concern specific to the IMP. Such AEs may require further investigation to characterise and understand them.

The following events, not occurring during the Dosing Visit (V3) or open label extension dosing (OLE2), are considered to be AESIs

- Mania
- Psychosis
- Suicide attempt or self-harm
- Hallucinogen persisting perception disorder

AESIs will be specifically enquired about during follow up visits and captured both narratively, as AEs and by formal scales, for example the HPPDS, DEPAS, AMRS and YMRS.

Adverse Events That Do Not Require Reporting

Since the IMP does not have a license or SmPC, all adverse events will be reported. Adverse events will be reported from the start of Dosing Visit (V3) until completion of follow up or until the end of the open label extension if the participant has consented to this, whichever is longer.

Treatment Stopping Rules

The trial may be prematurely discontinued by the Sponsor, Chief Investigator or Regulatory Authority on the basis of new safety information or for other reasons given by the Data Monitoring & Ethics Committee / Trial Steering Committee regulatory authority or ethics committee concerned.

If the trial is prematurely discontinued, active participants will be informed and no further participant data will be collected. The Competent Authority and Research Ethics Committee will be informed within 15 days of the early termination of the trial.

Statistics

Sample Size

We will recruit up to 60 participants in this study. The primary aim of the trial is to assess feasibility parameters for treatment and the trial (recruitment rates, loss to follow up) and estimate a variance parameter to inform a sample size calculation for a larger efficacy trial in the future. The expected rate of loss to follow-up is 20%. With 60 participants we would be able to estimate this target rate with a 95% confidence interval of ± 10.1% (9.9% to 30.1%). For the estimate of effect size, the modest sample sizes in pilot trials mean that this is sensitive to inflation [122]. To remedy this an increase of 1.15 should be applied to the variance for a sample size of 60.

Randomisation

Randomisation will be via an online, web-based system with strict allocation concealment that will email allocated treatment arm to Fisher Clinical Services, who will then label and package the treatment according to trial regulations with participant identification details. This package will then be sent by secure transport to the Maudsley Hospital Pharmacy, who will store the treatment prior to administration.

Analysis

The main purpose of the statistical analyses is to estimate various intervention and trial feasibility parameters and estimate the treatment effect size. The analysis will largely use descriptive statistics and effect sizes with inferential statistics used to generate respective confidence intervals and model effect sizes as appropriate.

The analyses can be split into two aims:

1. Estimation of parameters that evaluate intervention and trial feasibility (recruitment rates, randomisation rates, attrition rates, treatment satisfaction) and
2. Estimates of intervention effect sizes (mean differences, standard deviations) in terms of potential primary outcomes of future trials.

All statistical analyses will adopt the intention-to-treat (ITT) principle and will be carried out by the trial statistician, who is blind to treatment allocation. Participant flow will be presented in a CONSORT diagram. Analysis for the trial objectives will only be conducted after the completion of data collection.

Trial and Intervention Feasibility Parameters

Feasibility parameters will be assessed using descriptive statistics and confidence intervals. We will assess the rate of consent of eligible patients, study retention, loss to follow-up, the acceptability of psilocybin and deviations from study protocol. Confidence intervals will be estimated where appropriate. Participant demographic and clinical characteristics will be summarised at baseline. Patterns of missing data or nonattendance will be described for the outcome at measurement time-points and reasons for drop-out. Statistics derived will be means and standard deviations or medians, minimum, maximum and interquartile range for continuous measures and proportions for ordinal or multinomial categorical measures and binary coded measures.

Estimates for Effect Sizes for Psilocybin 25mg vs. Placebo.

To assess improvement in mood the primary analysis will estimate treatment effect at 3-week follow-up using ANCOVA with treatment group and baseline MADRS scores as fixed effects. Estimates of treatment effect sizes with 95% CI will be obtained using standardized regression coefficients with baseline standard deviation as the measure of variance. In addition, we will provide treatment effect scores from the raw data using between-group differences in the change from baseline. Using this we will calculate Cohen's D and U3, which gives the proportion of the active treatment group above the mean score of the control.

For the secondary outcomes at 3 weeks' follow-up and at other time-points (1 and 6 weeks), linear and non-linear mixed models will be used to take account of the statistical dependencies in the data due to repeated measures. The models will contain post-treatment measures of the outcome at the 4 follow up time points, i.e. 1, 3 and 6 weeks as the dependent variable with fixed effects of: trial arm (treatment vs placebo); baseline measure of outcome, time and a time-point by trial arm interaction to allow treatment effects to differ at 3 and 6 weeks. Including the baseline outcome measure as a covariate should gain precision for the treatment effect. A random effect for participant will be entered in to the model to account for correlations between the two repeated measures per participant. In addition, to account for the correlation of level-1 residuals across time, an exchangeable residual covariance structure will be included in the model. Effect sizes for within arm change will also be derived as providing useful information on group change within the trial setting. Linear mixed models will be used for continuous outcomes and logistic models for binary outcomes (e.g. 50% recovery on MADRS, yes or no).

With respect to missing data, linear mixed models will be estimated using maximum likelihood (ML). Such an approach provides valid inferences under the assumption that the missing data mechanism is ignorable (or missing at random, MAR). Here, this means that explanatory variables included in the model can predict missingness. We will include baseline predictors of drop-out as explanatory variables in the linear mixed model to make the MAR assumption more realistic. However, given the small dataset the number of explanatory variables to be included will be necessarily small. Model assumptions and sensitivity checks will be performed to assess the robustness of the statistical inference.

The frequency of serious adverse events and side effects and the number of participants having adverse events and side effects will be tabulated by treatment arm.

A full and detailed Statistical Analysis Plan will be drafted and signed off by the study team and DMEC.

Trial Steering Committee

We will follow NIHR guidance in setting up the TSC. A Trial Steering Committee (TSC) will meet twice a year and will be set up to monitor, review and supervise the progress of the trial. This will have an independent chair. We will invite up to five service user representatives onto the TSC. The TSC will set up a Data Monitoring and Ethics Committee (DMEC, see Section 12). Alongside our statistical expertise, the Steering Committee review will ensure that conduct and reporting standards (e.g. stipulated by the Nature Publishing Group) are adhered to. This will include use of appropriate methodology and subsequent reporting (i.e. CONSORT checklist items, design, recruitment, allocation, blinding, ethics, trial registration), and statistical procedures (i.e. stating assumptions, comprehensive reporting of results and providing clearly labelled descriptive statistics and figures, along with a CONSORT flowchart).

The role of the TSC will be to provide overall supervision for the trial on behalf of the Trial Sponsor and Trial Funder and to ensure that the trial is conducted to the rigorous standards set out in the Department of Health's Research Governance Framework for Health and Social Care and the Guidelines for Good Clinical Practice. We will carefully monitor adverse events and patient safety, and any concerns in this regard will be promptly taken to the Lead PI and TSC. The main features of the TSC are as follows:

- To provide advice, through its Chair, to the Chief Investigator(s), the Trial Sponsor, the Trial Funder and the Host Institution on all appropriate aspects of the trial.
- To concentrate on progress of the trial, adherence to the protocol, patient safety and the consideration of new information of relevance to the research question.
- The rights, safety and well-being of the trial participants are the most important considerations and should prevail over the interests of science and society.
- To ensure appropriate ethical and other approvals are obtained in line with the project plan.
- To agree proposals for substantial protocol amendments and provide advice to the sponsor and funder regarding approvals of such amendments.
- To provide advice to the investigators on all aspects of the trial.

Data Monitoring Committee

The Data Monitoring (and Ethics) Committee (DM(E)C) will be set up by the TSC and in practice the roles and meetings of these two committees will overlap given the size of this study. The DM(E)Cs main roles will be as follows:

- It will have access to the full data set.
- The role of its members will be to monitor these data and make recommendations to the TSC on whether there are any ethical or safety reasons why the trial should not continue. The safety, rights and well-being of the trial participants are paramount.
- The DM(E)C may be asked by the TSC, Trial Sponsor or Trial Funder to consider data emerging from other related studies.

If funding is required above the level originally requested, the DM(E)C may be asked by the Chief Investigator, TSC, Trial Sponsor or Trial Funder to provide advice and, where appropriate, information on the data gathered to date in a way that will not compromise the trial.

We do not plan an interim analysis given that the study is primarily aimed at feasibility.

Membership of the DM(E)C will be completely independent, small (3-4 members) and comprise experts in the field, e.g. a clinician with experience in the relevant area and expert trial statistician.

Responsibility for calling and organising DM(E)C meetings lies with the Chief Investigator, in association with the Chair of the DM(E)C. We will follow NIHR HTA guidance in setting up the TSC. The project team will provide the DM(E)C with a comprehensive report, the content of which should be agreed in advance by the Chair of the DM(E)C.

The DM(E)C will meet at least annually, or more often as appropriate, with meetings timed so that reports can be fed into the TSC.

The statisticians will draft a DMC DAMOCLES charter delineating roles and timings of meetings to be agreed at the first DMC meeting. This charter will sit with the KCTU and will be included in the trial master file.

Minutes of meetings will be sent to all members, the sponsor, the funder, the TSC and the trial master file.

Direct Access to Source Data and Documents

The Investigator(s) will permit trial-related monitoring, audits, REC review, and regulatory inspections by providing the Sponsor(s), Regulators and REC direct access to source data and other documents (e.g. patients' case sheets, blood test reports, electrocardiograms etc.).

Ethics & Regulatory Approvals

The trial will be conducted in compliance with the principles of the Declaration of Helsinki (1996), the principles of GCP and in accordance with all applicable regulatory requirements including (but not limited to) the Research Governance Framework and the Medicines for Human Use (Clinical Trial) Regulations 2004, as amended in 2006 and any subsequent amendments.

This protocol and related documents will be submitted for review to the London - Brent (Northwick Park Hospital, Boardroom, Level 7, Maternity Block, Harrow, Middlesex HA1 3UJ) Research Ethics Committee (REC), and to the Medicines and Healthcare products Regulatory Agency (MHRA) for Clinical Trial Authorisation. Any subsequent protocol amendments will submitted to the REC and Regulatory Authorities for approval

The Chief Investigator will submit a final report at conclusion of the trial to the KHP-CTO (on behalf of the Sponsor), the REC and the MHRA within the timelines defined in the Regulations.

Quality Assurance

To ensure compliance with Good Clinical Practice and scientific integrity, monitoring of this trial will be managed with oversight retained by the KHP-CTO Quality Team.

We will ensure that quality is optimally maintained throughout the trial, using methods such as: Trial Steering Committee monitoring, which includes individuals independent from the study team, ensuring regular and experienced supervision for team members, ensuring ongoing support and training for those undertaking research assessments and handling data.

The King’s CTU will be responsible for managing the following aspects of the trial: randomisation and statistics (in liaison with the trial statisticians).

The processes of eCRF creation and database design have been approved by the KHP Clinical Trials Office.

The Chief Investigator will maintain overall responsibility for the trial working closely with the Trial Manager to ensure that the trial is run in accordance with the protocol. A Trial Management Group (TMG) consisting of the Chief Investigator, co-investigators, the trial manager and the trial statistician will meet quarterly. The purpose of the TMG will be to oversee the smooth running of the trial and quality assurance. A full and detailed Data Management Plan will be drafted and signed off by the study team.

During the planning of this research project, we sought informal review by experienced academics, statisticians, clinicians and service users with lived experience of the condition and treatment under investigation. This was followed by a stringent peer review process for the funding application by the National Institute for Health Research (NIHR) Clinician Scientist Fellowship funding stream, where the project was judged sufficiently well designed to be funded, and to be worthwhile in terms of future implications and benefits to patients and the NHS. This protocol has been approved by the internal R&D department review processes of King’s College London and the South London and Maudsley NHS Foundation Trust.

Data Handling

The Chief Investigator will act as custodian for the trial data. The following guidelines will be strictly adhered to:

- Participant data will be anonymised
- All anonymised data will be stored on a password protected, encrypted computer.
- All trial data will be stored in line with the Medicines for Human Use (Clinical Trials) Amended Regulations 2006 and the General Data Protection Regulations and archived in line with the Medicines for Human Use (Clinical Trials) Amended Regulations 2006 as defined in the Kings Health Partners Clinical Trials Office Archiving SOP.

Data Management

Source data will be a variety of paper and electronic scales and an electronic case report form that can be printed and filled out as a fall-back method. Participants will rate scales during face to face visits at the trial facility, with a member of the study on hand to ensure data quality. Research data will be identified by a unique code and participant initials with restricted access. Electronically captured data will be time-stamped and changes to individual data points will be recorded. Electronic data will be backed up regularly via an encrypted connection to a secure server with second site resilience. We will adhere to NHS confidentiality practice, and to the Research Governance Framework in monitoring and managing the research. As CI, Professor Young will undertake overall responsibility for management of the project. The Data Management Plan details measures taken to promote data quality, for example, range checks for data values input into the eCRF.

Publication Policy

It is intended that the results of the study will be published in open access, peer-reviewed journals. The primary report will be attributed to the PsiDeR Investigators and Collaborators. The names of all members of the trial management team will be listed at the end of the primary publication. The primary publication will include all primary outcomes as listed in the protocol. Secondary and exploratory outcomes and ancillary analyses will be reported in additional publications.

In addition, we will present the findings at National and International conferences to reach as wide an audience as possible. We will use the university and NHS press offices to publicise findings as widely as possible to the public. The protocol will be publicly available but we will not grant public access to the participant level dataset and statistical code. Individual participants will not be identifiable from any publication. We have a PPI programme surrounding this research and close involvement with the IoPPN press office, given the tendency of this research to attract media interest. Service users with lived experience of depression will be involved in the PPI dissemination strategy planning and execution.

Study participants will be asked routinely whether they would like to be informed of the study findings and a summary will be sent to all those who request it. We will provide plain English reports for patient literature, linking with relevant patient organisations to do this, and aim to present the findings at relevant patient and user group meetings.

Insurance / Indemnity

The trial is co-sponsored by King's College London and South London and Maudsley NHS Foundation Trust. The sponsors will at all times maintain adequate insurance in relation to the study independently. King's College London, through its own professional indemnity (Clinical Trials) and no fault compensation and the Trust having duty of care to patients via NHS indemnity cover, in respect of any claims arising as a result of clinical negligence by its employees, brought by or on behalf of a study patient.

Financial Aspects

Funding to conduct this trial is provided by the National Institute for Health Research (NIHR) Clinician Scientist Fellowship Programme (CS-2017-17-007). Psilocybin 5mg and placebo capsules (or tablets) are provided by Compass Pathways, Ltd.

Signatures

The undersigned confirm that the following protocol has been agreed and accepted and that the Chief Investigator agrees to conduct the trial in compliance with the approved protocol and will adhere to the principles outlined in the Medicines for Human Use (Clinical Trials) Regulations 2004 (SI 2004/1031), amended regulations (SI 2006/1928) and any subsequent amendments of the clinical trial regulations, GCP guidelines, the Sponsor's (and any other relevant) SOPs, and other regulatory requirements as amended.

I agree to ensure that the confidential information contained in this document will not be used for any other purpose other than the evaluation or conduct of the clinical investigation without the prior written consent of the Sponsor

I also confirm that I will make the findings of the trial publicly available through publication or other dissemination tools without any unnecessary delay and that an honest accurate and transparent account of the trial will be given; and that any discrepancies and serious breaches of GCP from the trial as planned in this protocol will be explained.

**Statistician**		
Signature:		Date: 05-MAY-2022
Name (please print): Dr Ben Carter		
Position: Senior Lecturer in Biostatistics		
**Chief Investigator:**		
Signature:		Date: 05-MAY-2022
Name: (please print): Professor Allan H Young		
**Principal Investigator**		
Signature:		Date: 05-MAY-2022
Name: (please print): Dr James J Rucker		

References

1. McCrone, P., et al., *PAYING THE PRICE: The cost of mental health care in England to 2026*. 2008.
2. Health, D.o. *No health without mental health: A cross-Government mental health outcomes strategy for people of all ages. The economic case for improving efficiency and quality in mental health.* 2016 [cited 2016 28/11/2016].
3. Nemeroff, C.B., *Prevalence and management of treatment-resistant depression.* Journal of Clinical Psychiatry, 2007.
4. Crown, W.H., et al., *The Impact of Treatment-Resistant Depression on Health Care Utilization and Costs.* Journal of Clinical Psychiatry, 2002. **63**(11): p. 963–971.
5. Hyman, S.E., *Psychiatric drug development: diagnosing a crisis.* Cerebrum: the Dana Forum on Brain Science, 2013. **2013**: p. 5.
6. Grinspoon, L. and J. Bakalar, *Psychedelic Drugs Reconsidered*. 1997: The Lindesmith Center.
7. Cohen, S., *Lysergic acid diethylamide: side effects and complications.* The Journal of Nervous and Mental Disease, 1960. **130**(1): p. 30–40.
8. Strassman, R.J., *Adverse reactions to psychedelic drugs. A review of the literature.* The Journal of Nervous and Mental Disease, 1984. **172**(10): p. 577–595.
9. Johnson, M., W. Richards, and R. Griffiths, *Human hallucinogen research: guidelines for safety.* Journal of Psychopharmacology, 2008. **22**(6): p. 603–620.
10. Nichols, D.E., *Psychedelics.* Pharmacological Reviews, 2016. **68**(2): p. 264–355.
11. Grinspoon, L. and J. Bakalar, *The psychedelic drug therapies.* Current psychiatric therapies, 1981. **20**: p. 275–283.
12. Cohen, S. and K. Ditman, *Prolonged Adverse Reactions to Lysergic Acid Diethylamide.* Archives of General Psychiatry, 1963. **8**(5): p. 475.
13. Carhart-Harris, R.L. and D.J. Nutt, *User perceptions of the benefits and harms of hallucinogenic drug use: A web-based questionnaire study.* Journal of Substance Use, 2010. **15**(4): p. 283–300.
14. Johansen, P.O. and T.S. Krebs, *Psychedelics not linked to mental health problems or suicidal behavior: A population study.* Journal of Psychopharmacology, 2015. **29**(3): p. 270–279.
15. Hendricks, P.S., et al., *Classic psychedelic use is associated with reduced psychological distress and suicidality in the United States adult population.* Journal of Psychopharmacology, 2015. **29**(3): p. 280–288.
16. Krebs, T.S. and P.a.a.-Ø. Johansen, *Psychedelics and Mental Health: A Population Study.* PLoS One, 2013. **8**(8): p. e63972.
17. Rucker, J.J.H., et al., *Psychedelics in the treatment of unipolar mood disorders: A systematic review.* Journal of Psychopharmacology, 2016. **30**(12).
18. Rucker, J.J.H., J. Iliff, and D.J. Nutt, *Psychiatry & the psychedelic drugs. Past, present & future.* Neuropharmacology, 2017.
19. Hofmann, A., et al., *Psilocybin und Psilocin, zwei psychotrope Wirkstoffe aus mexikanischen Rauschpilzen.* Helvetica Chimica Acta, 1959. **42**(5): p. 1557–1572.
20. Madsen, M.K., et al., *Psychedelic effects of psilocybin correlate with serotonin 2A receptor occupancy and plasma psilocin levels.* Neuropsychopharmacology, 2019. **14**: p. 1.
21. Ray, T.S., *Psychedelics and the Human Receptorome.* PLoS One, 2010. **5**(2): p. e9019.
22. Gable, R.S., *Comparison of acute lethal toxicity of commonly abused psychoactive substances.* Addiction, 2004. **99**(6): p. 686–696.
23. Usdin, E. and D. Efron, *Psychotropic Drugs and Related Compounds*. Washington DC: Public Health Service, U.S. Dept of HEW.
24. Passie, T., et al., *The pharmacology of psilocybin.* Addiction biology, 2002. **7**(4): p. 357–364.

25. Studerus, E., et al., *Acute, subacute and long-term subjective effects of psilocybin in healthy humans: a pooled analysis of experimental studies.* Journal of Psychopharmacology, 2011. **25**(11): p. 1434–1452.

26. Fantegrossi, W.E., K.S. Murnane, and C.J. Reissig, *The behavioral pharmacology of hallucinogens.* Biochemical Pharmacology, 2008. **75**(1): p. 17–33.

27. Grob, C.S., et al., *Pilot Study of Psilocybin Treatment for Anxiety in Patients With Advanced-Stage Cancer.* Archives of General Psychiatry, 2011. **68**(1): p. 71–78.

28. Gasser, P., et al., *Safety and efficacy of lysergic acid diethylamide-assisted psychotherapy for anxiety associated with life-threatening diseases.* Journal of Nervous and Mental Disorders, 2014. **202**(7): p. 513–520.

29. Moreno, F.A., et al., *Safety, tolerability, and efficacy of psilocybin in 9 patients with obsessive-compulsive disorder.* Journal of Clinical Psychiatry, 2006. **67**(11): p. 1735–1740.

30. Johnson, M.W., et al., *Pilot study of the 5-HT2AR agonist psilocybin in the treatment of tobacco addiction.* Journal of Psychopharmacology, 2014. **28**(11): p. 983–992.

31. Bogenschutz, M.P., et al., *Psilocybin-assisted treatment for alcohol dependence: A proof-of-concept study.* Journal of Psychopharmacology, 2015. **29**(3): p. 289–299.

32. Sewell, R.A., J.H. Halpern, and H.G. Pope, *Response of cluster headache to psilocybin and LSD.* Neurology, 2006. **66**(12): p. 1920–1922.

33. Griffiths, R.R., et al., *Psilocybin produces substantial and sustained decreases in depression and anxiety in patients with life-threatening cancer: A randomized double-blind trial.* Journal of Psychopharmacology, 2016. **30**(12): p. 1181–1197.

34. Ross, S., et al., *Rapid and sustained symptom reduction following psilocybin treatment for anxiety and depression in patients with life-threatening cancer: a randomized controlled trial.* Journal of Psychopharmacology, 2016. **30**(12): p. 1165–1180.

35. Carhart-Harris, R.L., et al., *Psilocybin with psychological support for treatment-resistant depression: six-month follow-up.* Psychopharmacology, 2017. **235**(2): p. 399-408.

36. Carhart-Harris, R.L., et al., *Psilocybin with psychological support for treatment-resistant depression: an open-label feasibility study.* The Lancet Psychiatry, 2016. **3**(7).

37. Fekadu, A., J.G. Donocik, and A.J. Cleare, *Standardisation framework for the Maudsley staging method for treatment resistance in depression.* BMC Psychiatry, 2018. **18**(1): p. 100.

38. Fekadu, A., et al., *A Multidimensional Tool to Quantify Treatment Resistance in Depression.* Journal of Clinical Psychiatry, 2009. **70**(2): p. 177–184.

39. Nau Jr, F., et al., *Serotonin 5-HT2A Receptor Activation Blocks TNF-α Mediated Inflammation In Vivo.* PLoS One, 2013. **8**(10): p. e75426.

40. Yu, B., et al., *Serotonin 5-Hydroxytryptamine 2A Receptor Activation Suppresses Tumor Necrosis Factor-α-Induced Inflammation with Extraordinary Potency.* The Journal of pharmacology and experimental therapeutics, 2008. **327**(2): p. 316–323.

41. Zunszain, P.A., et al., *Glucocorticoids, cytokines and brain abnormalities in depression.* Progress in neuro-psychopharmacology & biological psychiatry, 2011. **35**(3): p. 722–729.

42. Hasler, F., et al., *Determination of psilocin and 4-hydroxyindole-3-acetic acid in plasma by HPLC-ECD and pharmacokinetic profiles of oral and intravenous psilocybin in man.* Pharmaceutica acta Helvetiae, 1997. **72**(3): p. 175–184.

43. Lindenblatt, H., et al., *Quantitation of psilocin in human plasma by high-performance liquid chromatography and electrochemical detection: comparison of liquid-liquid extraction with automated on-line solid-phase extraction.* Journal of chromatography. B, Biomedical sciences and applications, 1998. **709**(2): p. 255–263.

44. Hasler, F., et al., *Renal excretion profiles of psilocin following oral administration of psilocybin: a controlled study in man.* Journal of pharmaceutical and biomedical analysis, 2002. **30**(2): p. 331–339.

45. Wolbach, A.B., E.J. Miner, and H. Isbell, *Comparison of psilocin with psilocybin, mescaline and LSD-25.* Psychopharmacology, 1962. **3**(3): p. 219–223.

46. Leuner, H., *Die Experimentalle Psychose*. Vol. 95. 1962, Berlin, Heidelberg: Springer Berlin Heidelberg.

47. Leuner, H., *Halluzinogene in der Psychotherapie.* Pharmacopsychiatry, 1971. **4**(06): p. 333–351.

48. Isbell, H., *Comparison of the reactions induced by psilocybin and LSD-25 in man.* Psychopharmacology, 1959. **1**(1): p. 29–38.

49. Hill, R.M., R. Fischer, and D. Warshay, *Effects of excitatory and tranquilizing drugs on visual perception. Spatial distortion thresholds.* Experientia, 1969. **25**(2): p. 171–172.

50. Fischer, R., et al., *Psilocybin-induced contraction of nearby visual space.* Agents and Actions, 1970. **1**(4): p. 190–197.

51. Vollenweider, F., *Positron Emission Tomography and Fluorodeoxyglucose Studies of Metabolic Hyperfrontality and Psychopathology in the Psilocybin Model of Psychosis.* Neuropsychopharmacology, 1997. **16**(5): p. 357–372.

52. Hasler, F., et al., *Acute psychological and physiological effects of psilocybin in healthy humans: a double-blind, placebo-controlled dose-effect study.* Psychopharmacology, 2004. **172**(2): p. 145–156.

53. Griffiths, R.R., et al., *Psilocybin can occasion mystical-type experiences having substantial and sustained personal meaning and spiritual significance.* Psychopharmacology, 2006. **187**(3): p. 268–283.

54. Gouzoulis-Mayfrank, E., et al., *Psychopathological, neuroendocrine and autonomic effects of 3,4-methylenedioxyethylamphetamine (MDE), psilocybin and d-methamphetamine in healthy volunteers. Results of an experimental double-blind placebo-controlled study.* Psychopharmacology, 1999. **142**(1): p. 41–50.

55. Carter, O.L., et al., *Psilocybin links binocular rivalry switch rate to attention and subjective arousal levels in humans.* Psychopharmacology, 2007. **195**(3): p. 415–424.

56. Carter, O.L., et al., *Psilocybin impairs high-level but not low-level motion perception.* NeuroReport, 2004. **15**(12): p. 1947–1951.

57. Carhart-Harris, R.L., et al., *The administration of psilocybin to healthy, hallucinogen-experienced volunteers in a mock-functional magnetic resonance imaging environment: a preliminary investigation of tolerability.* Journal of Psychopharmacology, 2011. **25**(11): p. 1562–1567.

58. Pahnke, W.N., *The Psychedelic Mystical Experience in the Human Encounter with Death*. 1969.

59. Gouzoulis-Mayfrank, E., et al., *Psychological Effects of (S)-Ketamine and N,N-Dimethyltryptamine (DMT): A Double-Blind, Cross-Over Study in Healthy Volunteers.* Pharmacopsychiatry, 2005. **38**(6): p. 301–311.

60. Griffiths, R.R., et al., *Psilocybin occasioned mystical-type experiences: immediate and persisting dose-related effects.* Psychopharmacology, 2011. **218**(4): p. 649–665.

61. *GDS 2018 | Global Drug Survey*. 2020.

62. Hollister, L.E. and B.M. Sjoberg, *Clinical syndromes and biochemical alterations following mescaline, lysergic acid diethylamide, psilocybin and a combination of the three psychotomimetic drugs.* Comprehensive psychiatry, 1964. **5**(3): p. 170–178.

63. Wittmann, M., et al., *Effects of psilocybin on time perception and temporal control of behaviour in humans.* Journal of psychopharmacology (Oxford, England), 2007. **21**(1): p. 50–64.

64. Musha, M., et al., *Poisoning by hallucinogenic mushroom hikageshibiretake (Psilocybe argentipes K. Yokoyama) indigenous to Japan.* The Tohoku journal of experimental medicine, 1986. **148**(1): p. 73–78.

65. Peden, N.R. and S.D. Pringle, *Hallucinogenic fungi.* The Lancet, 1982. **1**(8268): p. 396–397.

66. Hyde, C., et al., *Abuse of indigenous psilocybin mushrooms: a new fashion and some psychiatric complications.* The British Journal of Psychiatry, 1978. **132**: p. 602–604.

67. Strassman, R.J. and C.R. Qualls, *Dose-response study of N,N-dimethyltryptamine in humans. I. Neuroendocrine, autonomic, and cardiovascular effects.* Archives of General Psychiatry, 1994. **51**(2): p. 85–97.

68. van Amsterdam, J. and A. Opperhuizen, *Harm potential of magic mushroom use: a review.* Regulatory Toxicology \ldots, 2011. **59**(3): p. 423–429.

69. Johnson, M.W., R. Andrew Sewell, and R.R. Griffiths, *Psilocybin dose-dependently causes delayed, transient headaches in healthy volunteers.* 2012. **123**(1-3): p. 132–140.

70. Hollister, L.E., *Clinical, biochemical and psychologic effects of psilocybin.* Archives internationales de pharmacodynamie et de therapie, 1961. **130**: p. 42–52.

71. Muthukumaraswamy, S.D., et al., *Broadband Cortical Desynchronization Underlies the Human Psychedelic State.* Journal of Neuroscience, 2013. **33**(38): p. 15171–15183.

72. Carhart-Harris, R.L., et al., *Neural correlates of the psychedelic state as determined by fMRI studies with psilocybin.* Proceedings of the National Academy of Sciences of the United States of America, 2012. **109**(6): p. 2138–2143.

73. Halpern, J.H. and H.G. Pope, *Do hallucinogens cause residual neuropsychological toxicity?* 1999. **53**(3): p. 247–256.

74. Benjamin, C., *Psychiatric symptoms and hallucinogenic compounds.* British Medical Journal, 1979. **2**(6188): p. 500.

75. Halpern, J.H. and H.G. Pope, *Hallucinogen persisting perception disorder: what do we know after 50 years?* 2003. **69**(2): p. 109–119.

76. Studerus, E., et al., *Prediction of psilocybin response in healthy volunteers.* PLoS One, 2012. **7**(2): p. e30800.

77. Carter, O.L., et al., *Modulating the rate and rhythmicity of perceptual rivalry alternations with the mixed 5-HT2A and 5-HT1A agonist psilocybin.* Neuropsychopharmacology, 2005. **30**(6): p. 1154–1162.

78. Vollenweider, F.X., et al., *The effects of the preferential 5-HT2A agonist psilocybin on prepulse inhibition of startle in healthy human volunteers depend on interstimulus interval.* Neuropsychopharmacology, 2007. **32**(9): p. 1876–1887.

79. Quednow, B.B., et al., *Psilocybin-Induced Deficits in Automatic and Controlled Inhibition are Attenuated by Ketanserin in Healthy Human Volunteers.* Neuropsychopharmacology, 2012. **37**(3): p. 630–640.

80. Vollenweider, F.X., et al., *Psilocybin induces schizophrenia-like psychosis in humans via a serotonin-2 agonist action.* NeuroReport, 1998. **9**(17): p. 3897–3902.

81. Blacker, K.H., et al., *Chronic users of LSD: the "acidheads".* American Journal of Psychiatry, 1968. **125**(3): p. 97–107.

82. MacLean, K.A., M.W. Johnson, and R.R. Griffiths, *Mystical experiences occasioned by the hallucinogen psilocybin lead to increases in the personality domain of openness.* Journal of Psychopharmacology, 2011. **25**(11): p. 1453–1461.

83. DeYoung, C.G., J.B. Peterson, and D.M. Higgins, *Sources of openness/intellect: cognitive and neuropsychological correlates of the fifth factor of personality.* Journal of personality, 2005. **73**(4): p. 825–858.

84. Hendricks, P.S., et al., *Hallucinogen use predicts reduced recidivism among substance-involved offenders under community corrections supervision.* Journal of Psychopharmacology, 2014. **28**(1): p. 62–66.

85. Klock, J.C., U. Boerner, and C.E. Becker, *Coma, Hyperthermia, and Bleeding Associated with Massive LSD Overdose a Report of Eight Cases.* Clinical toxicology, 1975.

86. Fantegrossi, W.E., J.H. Woods, and G. Winger, *Transient reinforcing effects of phenylisopropylamine and indolealkylamine hallucinogens in rhesus monkeys.* Behavioural pharmacology, 2004. **15**(2): p. 149–157.

87. Yanagita, T., *Intravenous self-administration of (-)-cathinone and 2-amino-1-(2,5-dimethoxy-4-methyl)phenylpropane in rhesus monkeys.* 1986. **17**(2-3): p. 135–141.

88. Nichols, D.E., *Hallucinogens.* Pharmacology & Therapeutics, 2004. **101**(2): p. 131–181.

89. Winstock, A.R., S. Kaar, and R. Borschmann, *Dimethyltryptamine (DMT): Prevalence, user characteristics and abuse liability in a large global sample.* Journal of Psychopharmacology, 2013. **28**(1): p. 49–54.

90. Franz, M., et al., *Magic mushrooms: hope for a 'cheap high' resulting in end-stage renal failure.* Nephrology, dialysis, transplantation : official publication of the European Dialysis and Transplant Association - European Renal Association, 1996. **11**(11): p. 2324–2327.

91. Lim, T.H., C.A. Wasywich, and P.N. Ruygrok, *A fatal case of 'magic mushroom' ingestion in a heart transplant recipient.* Internal medicine journal, 2012. **42**(11): p. 1268–1269.

92. Cameron, L.P., et al., *Effects of N, N-Dimethyltryptamine on Rat Behaviors Relevant to Anxiety and Depression.* ACS chemical neuroscience, 2018. **9**(7): p. 1582–1590.

93. Nutt, D.J., L.A. King, and L.D. Phillips, *Drug harms in the UK: a multicriteria decision analysis.* The Lancet, 2010. **376**(9752): p. 1558–1565.

94. Lingjaerde, O., et al., *The UKU side effect rating scale. A new comprehensive rating scale for psychotropic drugs and a cross-sectional study of side effects in neuroleptic-treated patients.* Acta Psychiatrica Scandinavica. Supplementum, 1987. **334**: p. 1–100.

95. Sheehan, D.V., et al., *The Mini-International Neuropsychiatric Interview (M.I.N.I.): the development and validation of a structured diagnostic psychiatric interview for DSM-IV and ICD-10.* Journal of Clinical Psychiatry, 1998. **59 Suppl 2**: p. 22–57.

96. Hamilton, M., *A RATING SCALE FOR DEPRESSION.* Journal of neurology, neurosurgery, and psychiatry, 1960. **23**(1): p. 56–62.

97. Montgomery, S.A. and M. \AAsberg, *A New Depression Scale Designed to be Sensitive to Change.* The British Journal of Psychiatry, 1979. **134**(4): p. 382–389.

98. Posner, K., et al., *The Columbia–Suicide Severity Rating Scale: Initial Validity and Internal Consistency Findings From Three Multisite Studies With Adolescents and Adults.* The American journal of psychiatry, 2011. **168**(12): p. 1266–1277.

99. Young, R.C., et al., *A Rating Scale for Mania: Reliability, Validity and Sensitivity.* The British Journal of Psychiatry, 1978. **133**(5): p. 429–435.

100. McGuire-Snieckus, R., et al., *A new scale to assess the therapeutic relationship in community mental health care: STAR.* Psychological Medicine, 2007. **37**(1): p. 85–95.

101. Rush, J.A., et al., *The 16-Item Quick Inventory of Depressive Symptomatology (QIDS), clinician rating (QIDS-C), and self-report (QIDS-SR): a psychometric evaluation in patients with chronic major depression.* Biological Psychiatry, 2003. **54**(5): p. 573–583.

102. Spitzer, R.L., et al., *A brief measure for assessing generalized anxiety disorder: the GAD-7.* Arch. Intern. Med., 2006. **166**(10): p. 1092–1097.

103. Pennebaker, J.W. and J.R. Susman, *Childhood Trauma Questionnaire. Measurement Instrument Database for the Social Sciences. Retrieved from www.midss.ie*. 2013.

104. Spielberger, C., et al., *Manual for the State Trait Anxiety Inventory*. 1983, Palo Alto: Consulting Psychologists Press.

105. Rosenbaum, J.F., et al., *Selective serotonin reuptake inhibitor discontinuation syndrome: a randomized clinical trial.* Biological Psychiatry, 1998. **44**(2): p. 77–87.

106. Studerus, E., A. Gamma, and F.X. Vollenweider, *Psychometric Evaluation of the Altered States of Consciousness Rating Scale (OAV).* PLoS One, 2010. **5**(8): p. e12412.

107. Altman, E.G., et al., *The Altman Self-Rating Mania Scale.* Biological Psychiatry, 1997. **42**(10): p. 948–955.

108. Derogatis, L.R., R.S. Lipman, and L. Covi, *SCL-90: an outpatient psychiatric rating scale–preliminary report.* Psychopharmacological Bulletin, 1973. **9**(1): p. 13–28.

109. Herdman, M., et al., *Development and preliminary testing of the new five-level version of EQ-5D (EQ-5D-5L).* Quality of life research : an international journal of quality of life aspects of treatment, care and rehabilitation, 2011. **20**(10): p. 1727–1736.

110. Mundt, J.C., et al., *The Work and Social Adjustment Scale: a simple measure of impairment in functioning.* Br. J. Psychiatry, 2002. **180**: p. 461–4.

111. Tellegen, A. and G. Atkinson, *Openness to absorbing and self-altering experiences ("absorption"), a trait related to hypnotic susceptibility.* Journal of abnormal psychology, 1974. **83**(3): p. 268–277.

112. Watson, D., L.A. Clark, and A. Tellegen, *Development and validation of brief measures of positive and negative affect: the PANAS scales.* Journal of personality and social psychology, 1988. **54**(6): p. 1063–1070.

113. Neff, K.D., *The Development and Validation of a Scale to Measure Self-Compassion.* Self and Identity, 2003. **2**(3): p. 223–250.

114. Tennant, R., et al., *The Warwick-Edinburgh Mental Well-being Scale (WEMWBS): development and UK validation.* Health and quality of life outcomes, 2007. **5**(1): p. 63.

115. Mayer, F.S. and C.M. Frantz, *The connectedness to nature scale: A measure of individuals' feeling in community with nature.* Journal of Environmental Psychology, 2004. **24**(4): p. 503–515.

116. Moran, P., et al., *Standardised Assessment of Personality – Abbreviated Scale (SAPAS): Preliminary validation of a brief screen for personality disorder.* The British Journal of Psychiatry, 2003. **183**(3): p. 228–232.

117. McIntyre, R.S., et al., *The THINC-Integrated Tool (THINC-it) Screening Assessment for Cognitive Dysfunction.* Journal of Clinical Psychiatry, 2017. **78**(7): p. 873–881.

118. Bland, A.R., et al., *EMOTICOM: A Neuropsychological Test Battery to Evaluate Emotion, Motivation, Impulsivity, and Social Cognition.* Frontiers in Behavioral Neuroscience, 2016. **10**(339): p. 17–25.

119. Williams, J.B.W. and K.A. Kobak, *Development and reliability of a structured interview guide for the Montgomery-\AAsberg Depression Rating Scale (SIGMA).* The British Journal of Psychiatry, 2008. **192**(1): p. 52–58.

120. Dittrich, A., *The standardized psychometric assessment of altered states of consciousness (ASCs) in humans.* Pharmacopsychiatry, 1998. **31 Suppl 2**(S 2): p. 80–84.

121. Patrick, F., et al., *Prescreening clinical trial volunteers using an online personality questionnaire.* Neuropsychiatric Disease and Treatment, 2018. **Volume 14**: p. 2297-2303.

122. Sim, J. and M. Lewis, *The size of a pilot study for a clinical trial should be calculated in relation to considerations of precision and efficiency.* Journal of clinical epidemiology, 2012. **65**(3): p. 301–308.
