# Supplementary material for: Psilocybin-assisted therapy for treatment-resistant major depressive disorder in a public healthcare setting: a randomized controlled trial
Source: Nat Med. 2026 Aug 6;32(9):3430–7. doi: 10.1038/s41591-026-04541-0 (PMC13577891; doi:10.1038/s41591-026-04541-0)
Supplement: Supplementary file 2 [file 41591_2026_4541_MOESM2_ESM.pdf]

Corresponding author(s): James Rucker

Last updated by author(s): Mar 6, 2026

Reporting Summary

Nature Portfolio wishes to improve the reproducibility of the work that we publish. This form provides structure for consistency and transparency in reporting. For further information on Nature Portfolio policies, see our Editorial Policies and the Editorial Policy Checklist.

Statistics

For all statistical analyses, confirm that the following items are present in the figure legend, table legend, main text, or Methods section.

n/a	Confirmed	
☐	☒	The exact sample size (*n*) for each experimental group/condition, given as a discrete number and unit of measurement
☐	☒	A statement on whether measurements were taken from distinct samples or whether the same sample was measured repeatedly
☐	☒	The statistical test(s) used AND whether they are one- or two-sided<br>*Only common tests should be described solely by name; describe more complex techniques in the Methods section.*
☐	☒	A description of all covariates tested
☐	☒	A description of any assumptions or corrections, such as tests of normality and adjustment for multiple comparisons
☐	☒	A full description of the statistical parameters including central tendency (e.g. means) or other basic estimates (e.g. regression coefficient) AND variation (e.g. standard deviation) or associated estimates of uncertainty (e.g. confidence intervals)
☐	☒	For null hypothesis testing, the test statistic (e.g. *F*, *t*, *r*) with confidence intervals, effect sizes, degrees of freedom and *P* value noted<br>*Give P values as exact values whenever suitable.*
☒	☐	For Bayesian analysis, information on the choice of priors and Markov chain Monte Carlo settings
☐	☒	For hierarchical and complex designs, identification of the appropriate level for tests and full reporting of outcomes
☐	☒	Estimates of effect sizes (e.g. Cohen's *d*, Pearson's *r*), indicating how they were calculated

Our web collection on statistics for biologists contains articles on many of the points above.

Software and code

Policy information about availability of computer code

Data collection	No software was used
Data analysis	Code used for analyses can be found at https://github.com/petrinachu/psider

For manuscripts utilizing custom algorithms or software that are central to the research but not yet described in published literature, software must be made available to editors and reviewers. We strongly encourage code deposition in a community repository (e.g. GitHub). See the Nature Portfolio guidelines for submitting code & software for further information.

Data

Policy information about availability of data

All manuscripts must include a data availability statement. This statement should provide the following information, where applicable:

- Accession codes, unique identifiers, or web links for publicly available datasets
- A description of any restrictions on data availability
- For clinical datasets or third party data, please ensure that the statement adheres to our policy

Access to deidentified participant data may be available to researchers who provide a methodologically sound proposal to the corresponding author, beginning 9 months after publication, for 36 months.

Research involving human participants, their data, or biological material

Policy information about studies with human participants or human data. See also policy information about sex, gender (identity/presentation), and sexual orientation and race, ethnicity and racism.

Reporting on sex and gender	Participants' sex is reported in the manuscript; equal numbers of males and females were included in the study. Sex was a considered in the study design; it was a minimisation factor in the randomisation sequence. No sex- or gender-based subgroup analyses were performed as they would be severely underpowered in this feasibility sample.
Reporting on race, ethnicity, or other socially relevant groupings	Participants self-reported the following information which has been used to describe the sample:<br>Ethnicity<br>Highest level of completed education<br>Monthly take-home household income<br>Employment category<br>Current occupational status<br>Sexual orientation
Population characteristics	See below.
Recruitment	Participants were recruited via primary and secondary care services, established registries of research-interested patients, and advertising in the community and social media. We aimed to recruit a representative sample with regards to ethnicity and sexual orientation, using UK census data to create targets for participant recruitment. Because there are differences in demographics between London and the rest of the UK and we expected participants from both within and outside of London, targets were based on the average of the London population and the rest of the UK.
Ethics oversight	London, Brent Research Ethics Committee (reference: 20/LO/0206)

Note that full information on the approval of the study protocol must also be provided in the manuscript.

Field-specific reporting

Please select the one below that is the best fit for your research. If you are not sure, read the appropriate sections before making your selection.

☐ Life sciences ☒ Behavioural & social sciences ☐ Ecological, evolutionary & environmental sciences

For a reference copy of the document with all sections, see nature.com/documents/nr-reporting-summary-flat.pdf

Behavioural & social sciences study design

All studies must disclose on these points even when the disclosure is negative.

Study description	Quantitative - randomised controlled trial
Research sample	60 adults with treatment resistant depression. 30 male, 30 female. Median (IQR) age = 40.8 (35.2 - 52.3). 75% White, 10% Mixed or multiple ethnic groups, 12% Asian or Asian British, 2% Black, African, Caribbean, or Black British, 2% other ethnic group.
Sampling strategy	The target sample size was 60 participants. The study was designed as a feasibility study to estimate the drop-out rate (20%) with a 95% confidence interval (CI) of ± 10.1% (9.9% to 30.1%).
Data collection	Patient rated scales were completed on a computer with a researcher present. Both participants and researchers were blinded. The future primary outcome (MADRS) was administered by blinded assessors, who were independent of the trial team, but not the institution undertaking the trial.
Timing	Start: 21 June 2021, end of RCT data collection: 28 Aug 2024
Data exclusions	No data were excluded
Non-participation	One participant withdrew prior to week 1 follow-up; the participant no longer wished to participate for personal reasons.
Randomization	Participants were randomised (1:1) to receive 25mg psilocybin or placebo, with the sequence generated using minimisation by sex (male, female), age (25 to 59, ≥60), and past psilocybin use (yes, no), by a web-based service hosted by King's Clinical Trials Unit (KCTU) and concealed from trial team.

Reporting for specific materials, systems and methods

We require information from authors about some types of materials, experimental systems and methods used in many studies. Here, indicate whether each material, system or method listed is relevant to your study. If you are not sure if a list item applies to your research, read the appropriate section before selecting a response.

Materials & experimental systems

n/a	Involved in the study
☒	☐ Antibodies
☒	☐ Eukaryotic cell lines
☒	☐ Palaeontology and archaeology
☒	☐ Animals and other organisms
☐	☒ Clinical data
☒	☐ Dual use research of concern
☒	☐ Plants

Methods

n/a	Involved in the study
☒	☐ ChIP-seq
☒	☐ Flow cytometry
☒	☐ MRI-based neuroimaging

Clinical data

Policy information about clinical studies

All manuscripts should comply with the ICMJE guidelines for publication of clinical research and a completed CONSORT checklist must be included with all submissions.

Clinical trial registration	Medicines and Healthcare Products Regulatory Agency (EudraCT no: 2018-003573-97); clinicaltrials.gov (NCT04959253)
Study protocol	Enclosed with the submission, and published version is Rucker J, Jafari H, Mantingh T, et al. Psilocybin-assisted therapy for the treatment of resistant major depressive disorder (PsiDeR): protocol for a randomised, placebo-controlled feasibility trial. BMJ Open 2021; 11: e056091
Data collection	60 participants were recruited between 21 June 2021 and 03 July 2024 at one NHS site in the UK (South London and Maudsley NHS Foundation Trust).
Outcomes	All outcome measures and analyses can be found in the Statistical Analysis Plan (supplementary material).

Plants

Seed stocks	*Report on the source of all seed stocks or other plant material used. If applicable, state the seed stock centre and catalogue number. If plant specimens were collected from the field, describe the collection location, date and sampling procedures.*
Novel plant genotypes	*Describe the methods by which all novel plant genotypes were produced. This includes those generated by transgenic approaches, gene editing, chemical/radiation-based mutagenesis and hybridization. For transgenic lines, describe the transformation method, the number of independent lines analyzed and the generation upon which experiments were performed. For gene-edited lines, describe the editor used, the endogenous sequence targeted for editing, the targeting guide RNA sequence (if applicable) and how the editor was applied.*
Authentication	*Describe any authentication procedures for each seed stock used or novel genotype generated. Describe any experiments used to assess the effect of a mutation and, where applicable, how potential secondary effects (e.g. second site T-DNA insertions, mosiacism, off-target gene editing) were examined.*
